# Chiral Sodium Glycerophosphate Catalyst for Enantioselective Michael Reactions of Chalcones

**DOI:** 10.3390/molecules29194763

**Published:** 2024-10-08

**Authors:** Giovanni Ghigo, Julia Rivella, Alessio Robiolio Bose, Stefano Dughera

**Affiliations:** Department of Chemistry, University of Torino, Via Pietro Giuria 7, 10125 Torino, Italy; julia.rivella@edu.unito.it (J.R.); alessio.robioliobose@edu.unito.it (A.R.B.)

**Keywords:** conjugate addition, organocatalyst, chiral catalyst

## Abstract

A chiral sodium glycerophosphate is successfully exploited as a catalyst in the Michael addition of methyl malonate to a number of chalcones. The reactions supplied the target adducts in satisfactory yields and good enantiomeric excesses. A tentative computational study is presented, aiming to understand the reaction mechanism.

## 1. Introduction

**Figure 1:**
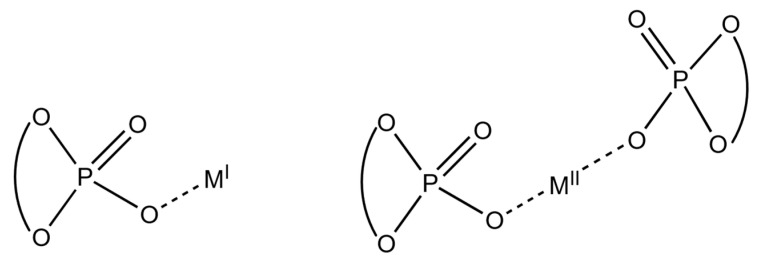
Metal phosphates.

The employment of alkali and alkaline earth metal phosphates (Figure 1) in asymmetric catalysis [1,2,3], even though strictly not classifiable as asymmetric organocatalysts, displays many of their advantages, namely the handiness of the catalyst, the absence of costly and toxic heavy transition metals, and the ease of preparation from the corresponding phosphoric acids [4,5].

**Figure 2:**
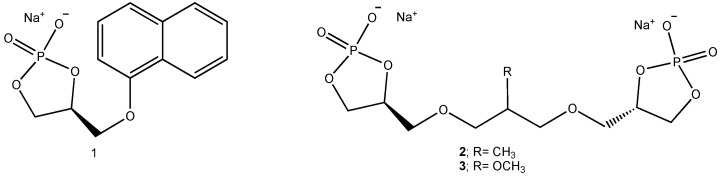
Chiral cycloglycerophosphates.

On this ground, we recently proposed a new class of sodium phosphate catalysts, namely cycloglycerophosphates (Figure 2) prepared from enantiopure solketal (the acetonide of glycerol) [6]. One of these adducts, namely (S)-4-((naphthalen-1-yloxy)methyl)-1,3,2-dioxaphospholan-2-olate 2-oxide sodium (Figure 2; **1**), was employed as a catalyst in the addition of TMSCN to aldehydes at room temperature, to provide *gem*-cyanohydrins [6]. Unfortunately, no enantioselectivity was observed. This behavior can be explained with an intrinsically low asymmetric induction exerted by the five-membered phosphatidic acid scaffold, having a stereogenic center far from the catalyst’s active site and not supported by steric hindrance effects, because of the free rotation around the exocyclic bond bearing the bulky α-naphthyloxy group. On the other hand, in order to introduce C2-symmetry we prepared adducts **2** and **3** (Figure 2) [7]. They resulted into an outstanding enantiocontrol of the reaction, namely the addition of TMSCN to aldehydes and ketones. The reaction mechanism was investigated on a minimal model of the catalyst with a DFT method. Instead, a calculation of the most stable conformation of the model catalyst for both C2-symmetric cycloglycerodiphosphates accounts for the enantioselectivity, since the two cyclic phosphatidic moieties are *syn*, creating a confined pocket for the reactants through electrostatic interactions involving the sodium cations [7].

In light of the interesting results previously obtained and with the aim of expanding the synthetic uses of this class of catalysts, we studied 1,4 addition reactions of methyl malonate (**4**) to several chalcones **5** in the presence of **2** as a chiral catalyst (Table 1 and Table 2). It must be stressed that the catalytic asymmetric conjugate addition of α,ß,-unsaturated carbonyl compounds is one of the most powerful carbon–carbon bond-forming reactions [8,9]. In fact, its complete atom economy, wide substrate scope, susceptibility to many classes of catalysts, and easily accessible starting materials render it one of the most modern out of all classical reactions. However, on the other hand, these reactions remain difficult to execute via either stoichiometric or catalytic approaches, despite recent advances [10].

The chiral catalysts used for this reaction are most varied [11]. However, the literature shows few examples catalyzed by chiral phosphates, three of them being the most significant. In particular, in 2010, Chen reported the enantioselective addition of TMSCN to aromatic enones catalyzed by the sodium salt of (*R*)-3,3′-di(1-adamantyl)-1,1′-binaphthyl-2,2′-diylphosphoric acid [12], and Antilla, in 2011, described a chiral vapol phosphate-catalyzed Michael reaction of 3-substituted indoles with methyl vinyl ketone [13]. Finally, in 2019, Zhu and Niemeyer reported that heterobifunctional rotaxanes featuring an amine-based thread and a chiral 1,1′-binaphthylphosphoric acid-based macrocycle efficiently catalyzed the addition of malonates to Michael acceptors [14].

## 2. Results and Discussion

As a model reaction, we decided to begin the study by reacting methyl malonate (**4**) with *E*-1,3-diphenylpropen-3-one (**5a**) in the presence of catalyst **2** at room temperature and with a number of solvents (Table 1; Entries 8, 9, 10, 11). Only traces of target compound dimethyl 2-(3-oxo-1,3-diphenylpropyl)malonate) **6a** were detected using MeOH (Table 1; Entry 2) or 2-MeTHF (Table 1; Entry 5) as solvents. Also increasing the amount of catalyst to 10 mol% (Table 1; Entries 3,6) and carrying out the reaction at 60 °C (Table 1; Entries 4,7), no appreciable results were obtained. Since catalyst **2** is not basic enough to deprotonate the malonate and, therefore, allow a nucleophilic attack against **5a**, in order to transform the malonate into the corresponding more nucleophilic anion, we added an equimolar amount of sodium methoxide to **4**, dissolved in methanol.

Under these conditions, with both 5 and 10 mol% of catalyst **2** (Table 1; Entries 12,13), a fairly good yield of target **6a** was obtained (52%), although with a low enantiomeric excess (34%). On the contrary, in toluene, no appreciable results were obtained (Table 1; Entry 14). Even using a stronger base (NaH), there was no improvement (Table 1; Entry 15). Therefore, we decided to change the solvent and use 2-THF. No substantial improvement occurred with sodium methoxide as the base (Table 1; Entry 16). After changing the base and using a stronger NaH (Table 1; Entry 17), the yield of target **6a** significantly increased up to 90%, but the enantiomeric excess was similar (37%) to that previously obtained.

By lowering the temperature to 0°, an enantiomeric excess of 57% (Table 1; Entry 18) was obtained. By further lowering the temperature to −20 °C, to our delight, the enantiomeric excess was 91.5% (Table 1; Entry 19). A further lowering of the temperature to −45 °C did not lead to changes (Table 1; Entry 20). Moreover, this reaction was not completed after 8 h. On the other hand, it is necessary to underline that the reaction occurred between the anion of **4** and **5a** even in the absence of catalyst **2** (Table 1; Entries 21,22).

In order to explore the applicability of this optimized procedure, we tested other reactions between aromatic or heteroaromatic chalcones, variously substituted with electron-withdrawing or electron-donating substituents (Table 2; Entries 1–20). The yields were good to excellent, and good enantiomeric excesses were obtained, always between 77 and 92%. The average enantiomeric excess for 20 examples was 90.2%.

Moving on to examine the reactivity of aliphatic chalcones (or similar compounds), quite surprisingly, no reactions occurred with methyl vinyl ketone, methyl acrylate, or acrylaldehyde. We believe that, due to the lack of an electron-donating and activating group linked to the C atom in ß of CO, the nucleophilic attack of malonate could not occur. Instead, compounds **5u**, **5v**, and **5w** showed the same reactivity as the corresponding aromatic ones (Table 2; Entries 21–23), even if the enantiomeric excesses obtained were lower (the average enantiomeric excess for tree examples was about 83.2%).

**Figure 3:**
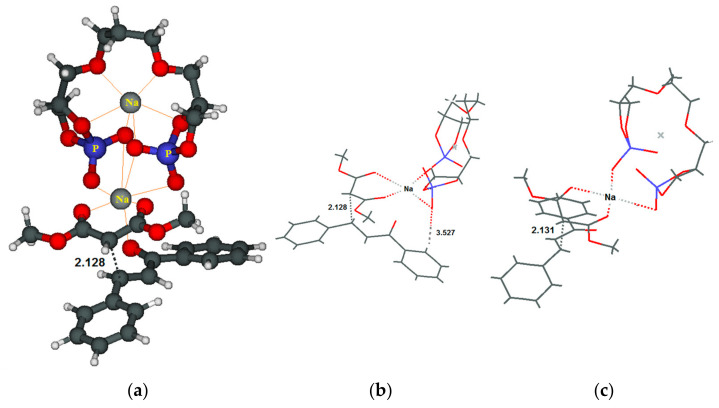
Transition structures for nucleophilic addition: (**a**) ball-and-stick view of **TS(R)** yielding the R product; (**b**) skeleton view of **TS(R)** yielding the R product; and (**c**) skeleton view of **TS(S)** yielding the S product.

In an attempt to rationalize the experimental outcomes, we performed a computational study on the reaction with chalcone **5a** (see Supplementary Materials for details on the method and a more extended discussion). In the structure of the transition state (**TS(R)**) for the nucleophilic addition yielding the R product (Figure 3a), the catalyst is depicted on the upper part of the figure; the upper sodium cation is fully solvated by the oxygen atoms (in red) of the catalyst, while the second, on the lower part, interacts also with the oxygen atoms of the malonate (in the middle) and the chalcone (in the lower part).

In the skeleton view (Figure 3b) of **TS(R)**, we can observe an interaction between an oxygen of the catalyst atom carrying a negative charge and the phenyl ring bound to the electron-withdrawing carbonyl group, which is absent in the TS yielding the S product (TS(S), Figure 3c). This extra interaction in TS(R) seems to be responsible for the slightly lower energy (E + ZPE = 7.7 vs. 8.2 kcal mol^−1^) on this TS with respect to TS(S).

We believe that, thanks to these interactions in **TS(R)**, the nucleophilic attack of the anion of **4** against **5** can occur mainly in a single direction, allowing **6** to be obtained with a good enantiomeric excess, qualitatively reproduced by the calculation. As already mentioned previously (Table 1, Entry 21), the reaction occurs even in the absence of catalyst **2**. Therefore, its essential function is to create a chiral fashion.

We also tried to model the effect of the catalyst on the kinetics of the reaction. A small increase in the catalyzed reaction rate was observed, but the computations failed to fully reproduce it. However, the computed energy difference between the non-catalyzed and catalyzed reactions was very small (0.5 kcal mol^−1^) and strongly dependent on the solvent effects, which are very difficult to fully reproduce.

## 3. Materials and Methods

### 3.1. General

Catalyst **2** [7] and chalcone **5** [15] were prepared as reported in the literature. Analytical-grade reagents and solvents (purchased from Merck, Carlo Erba, Alfa Aesar, and Fluorochem) were used, and the reactions were monitored by GC and GC-MS. Column chromatography and TLC were performed on Merck silica gel 60 (70-230 mesh ASTM) and GF 254, respectively. Petroleum ether (PE) refers to the fraction boiling in the range of 40–70 °C. GC analyses were performed on a PerkinElmer AutoSystem XL GC with a methylsilicone capillary column. Mass spectra were recorded on an HP5989B mass-selective detector connected to an HP 5890 GC with a cross-linked methyl silicone capillary column. Chiral analyses were performed on an HPLC using a Daicel CHIRALPAK-IG (250 × 4.6 mm, 5 mm) on Essential LC- 16 series, eluting with i-PrOH and n-hexane. For the determination of the optical rotation, a Jasco P-2000 polarimeter was used. ^1^H NMR and ^13^C NMR spectra were recorded on a Brucker spectrometer at 400 and 100 MHz, respectively. IR spectra were recorded on an IR Perkin-Elmer UATR-two spectrometer. The structures and purity of all the adduct **6** obtained in this research were confirmed by the spectral (NMR, GC-MS, IR) data, reported in Supplementary Materials. Satisfactory microanalyses were obtained for all new compounds.

### 3.2. Catalyst 2 in Michael Reactions: Typical Procedure and Synthesis of (R)(-)-dimethyl 2-(3-oxo-1,3-diphenylpropyl)malonate (***6a***)

NaH (60% dispersion in paraffin liquid; 40 mg, 1 mmol) was added to a stirring solution of methyl malonate (**4**; 132 mg, 1 mmol) in 2-MeTHF (5 mL). A white suspension was immediately formed. After 10 min of further stirring, the mixture was cooled to −20 °C. At this point, a chiral catalyst (**2**; 5 mol%, 21 mg, 0.05 mmol) and *E*-1,3-diphenylprop-2-en-1-one (**5a**; 208 mg, 1 mmol) were added. The mixture was stirred for 8 h, until the TLC (eluent PE/EE 4:1) GC and GC-MS analyses showed the complete disappearance of **4** and **5a** and the complete formation of dimethyl 2-(3-oxo-1,3-diphenylpropyl)malonate (**6a**; MS (70 eV) *m*/*z*: 340 (37) [M]^+^). Then, H_2_O (5 mL) was added to the reaction mixture, which was extracted with Et_2_O (10 mL). The organic phase was washed with H_2_O (5 mL) and dried over sodium sulfate. The solvent was evaporated under reduced pressure. The crude residue was purified in a short chromatographic column (eluent: PE/EE 4:1). The resulting white solid was a virtually pure title compound (**6a**; 305 mg; 90% yield). After the above work-up, the *Ee* was measured.

Under the same conditions, a Gram-scale reaction was carried out using 5 mmol of **5a** (1.04 g), **4** (0.66 g), and NaH (0.20 g) in the presence of catalyst **2** (5 mol%, 105 mg, 0.25 mmol).

After the above work-up, **6a** was obtained (1.48 g, 87%), and the *Ee* was 91.2%.

## 4. Conclusions

We have hereby reported the enantioselective conjugate addition of methyl malonate (**4**) to chalcone **5** catalyzed by C2-symmetric cycloglycerodiphosphate **2**. The target adduct **6** was obtained in satisfactory yields, with good enantiomeric excess. In light of this and its ease of preparation and purification, this catalyst is very competitive for this type of asymmetric protocol, since the results are comparable to those obtained with other catalytic systems dedicated to this purpose and already cited in the literature.

## Figures and Tables

**Table 1:** Trial reactions.

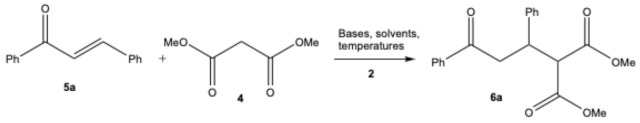							
Entry	Catalyst 2 (mmol%)	Base	Solvent	T (°C)	Time (h)	Yield (%) ^1,2^	*Ee* (%) ^3^
1	-	-	MeOH	rt	24	traces	-
2	5	-	MeOH	rt	24	traces	-
3	10	-	MeOH	rt	24	traces	-
4	10		MeOH	60	24	traces	-
5	5	-	2-MeTHF	rt	24	traces	-
6	10	-	2-MeTHF	rt	24	traces	-
7	10	-	2-MeTHF	60	24	traces	-
8	5	-	Toluene	rt	24	-	-
9	5	-	DCM	rt	24	-	-
10	5	-	DMSO	rt	24	-	-
11	5	-	MeCN	rt	24	-	-
12	5	CH_3_ONa	MeOH	rt	8	52	34.3
13	10	CH_3_ONa	MeOH	rt	6	51	32.8
14	5	CH_3_ONa	Toluene	rt	24	-	-
15	5	NaH	MeOH	rt	6	55	33.1
16	5	CH_3_ONa	2-MeTHF	rt	8	45	34.5
17	5	NaH	2-MeTHF	rt	3	92	37.5
18	5	NaH	2-MeTHF	0	5	90	57.2
19	5	NaH	2-MeTHF	−20	8	90	91.5
20	5	NaH	2-MeTHF	−45	8	21	90.8
21	-	NaH	2-MeTHF	rt	3	91	-
22	-	NaH	2-MeTHF	−20	8	90	-

^1^ Reactants **5a**, **4** and MeONa/NaH are in a ratio of 1:1:1. ^2^ Yields refer to pure and isolated **6a**, purified in a short chromatographic column (eluent petroleum ether/diethyl ether 3:2). ^3^ *Ee* was determined by HPLC analyses on a chiral stationary phase.

**Table 2:** Synthesis of adduct **6**.

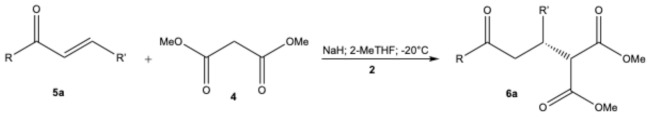					
Entry	R in 5,6	R’ in 5,6	Time (h)	Yield (%) of 6 ^1,2^	*Ee* (%) ^3^
1	C_6_H_5_	C_6_H_5_	8	**6a**; 90	91.5
2	C_6_H_5_	4-NO_2_C_6_H_4_	10	**6b**; 87	88.9
3	C_6_H_5_	4-ClC_6_H_4_	6	**6c**; 89	92.9
4	C_6_H_5_	4-MeC_6_H_4_	4	**6d**; 92	90.0
5	C_6_H_5_	4-CNC_6_H_4_	10	**6e**; 92	90.3
6	C_6_H_5_	2-NO_2_C_6_H_4_	12	**6f**; 77	90.5
7	C_6_H_5_	3-NO_2_C_6_H_4_	8	**6g**; 90	89.9
8	C_6_H_5_	Thiophen-2-yl	6	**6h**; 82	89.6
9	C_6_H_5_	Furan-2-yl	6	**6i**; 77	89.3
10	Me	C_6_H_5_	6	**6j**; 82	92.5
11	Me	2-MeC_6_H_4_	8	**6k**; 90	94.4
12	Me	3-MeOC_6_H_4_	6	**6l**; 85	88.8
13	C_6_H_5_	Me	4	**6m**; 87	89.5
14	4-CF_3_C_6_H_4_	C_6_H_5_	6	**6n**; 92	89.7
15	2-MeOC_6_H_4_	C_6_H_5_	6	**6o**; 79	87.0
16	3-MeC_6_H_4_	4-ClC_6_H_4_	8	**6p**; 82	89.9
17	3-MeC_6_H_4_	4-NO_2_C_6_H_4_	10	**6q**; 91	87.0
18	4-CF_3_C_6_H_4_	4-MeC_6_H_4_	6	**6r**; 90	92.4
19	4-ClC_6_H_4_	C_6_H_5_	6	**6s**; 85	89.0
20	4-NO_2_C_6_H_4_	C_6_H_5_	6	**6t**; 85	90.5
21	Et	Me	4	**6u**; 85	81.8
22	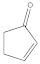		6	**6v**; 83	87.5
23	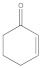		6	**6w**; 91	70.2

^1^ Reactants **5**, **4** and NaH are in a ratio of 1:1:1. The amount of catalyst **2** is 5 mol%. ^2^ Yields refer to pure and isolated **6**, purified in a short chromatographic column (eluent petroleum ether/diethyl ether 3:2). ^3^ *Ee* was determined by HPLC analyses on a chiral stationary phase.

## Data Availability

The data reported in this article can be obtained from the authors upon reasonable request. Samples of compound **6** are available from the authors.

## Supplementary Materials

The following supporting information can be downloaded at https://www.mdpi.com/article/10.3390/molecules29194763/s1: physical data of malonate **6**, on page S-2; NMR spectra and chiral analyses of malonate **6**, on page S-7; computational method, on page S-54; schemes, tables, pictures, and Cartesian coordinates for the uncatalyzed reaction, on page S-56; and schemes, tables, pictures, and Cartesian coordinates for the catalyzed reaction, on page S-67 [16,17,18,19,20,21,22,23,24,25,26,27,28,29,30,31,32,33,34].
