# Supplementary material for: Chiral Sodium Glycerophosphate Catalyst for Enantioselective Michael Reactions of Chalcones
Source: Molecules. 2024 Oct 8;29(19):4763. doi: 10.3390/molecules29194763 (PMC11477885; doi:10.3390/molecules29194763)
Supplement: Supplementary file 1 [file molecules-29-04763-s001.zip › molecules-3238205-supplementary.pdf]

Chiral sodium glicerophosphate catalyst for enantioselective Michael reactions of chalcones.

Giovanni Ghigo*, Julia Rivella, Alessio Robiolio Bose, Stefano Dughera*

Index.

Physical data of malonates **6**. Pag. S-2

NMR Spectra and chiral analyses of malonates **6.** Pag. S-7

Computational method. Pag. S-54

Schemes, tables, pictures and Cartesian coordinates:

The uncatalyzed reaction. Pag. S-56

The catalyzed reaction. Pag. S-67

Physical data of malonates 6

(*R*)(-)-Dimethyl 2-(3-oxo-1,3-diphenylpropyl)malonate (6a). White solid (307 mg, 90% yield); m.p 104–106°C (Lit.[1] 103–105 °C); [α]D_{20}= -11.13 (c=0.15 in $CHCl_3$; Lit.[2] -13.10 for R enantiomer); ^{1}H NMR (400 MHz, $CDCl_3$): δ = 7.91 (d, $^1J_{H-H}$=8.0Hz, 2H), 7.57–7.53 (m, 1H), 7.46–7.42 (m, 2H), 7.28–7.27 (m, 4H), 7.23–7.17 (m, 1H), 4.25–4.18 (m, 1H), 3.88 (d, $^1J_{H-H}$=9.2Hz, 1H), 3.60 (s, 3H), 3.58–3.47 (m, 5H); ^{13}C NMR (100 MHz, $CDCl_3$): δ=197.5, 168.7, 168.1, 140.4, 136.8, 133.1, 128.6, 128.5, 128.1, 127.2, 57.2, 52.6, 52.4, 42.3, 40.8; IR (neat) ν=1715 (CO), 1667 cm^{-1} (CO). MS (70 eV) *m/z* (%): 340 (5) [M]$^+$,249 (14), 209 (57), 105 (100). 91.5% ee, t_R=9.126 min (minor), t_R =11.839 min (major).

(*R*)(-)-Dimethyl 2-(1-(4-nitrophenyl)-3-oxo-3-phenylpropyl)malonate (6b). White solid (337 mg, 87% yield); m.p 77–78°C (Lit.[3] 74–76); [α]D_{20}= -23.15 (c=0.15 in $CHCl_3$; Lit.[3] 33.5 for S enantiomer); ^{1}H NMR (400 MHz, $CDCl_3$): δ = 8.01 (d, $^1J_{H-H}$=8.8Hz, 2H), 7.79 (d, $^1J_{H-H}$=7.2Hz, 2H), 7.47–7.31 (m, 5H), 4.26–4.19 (m, 1H), 3.82 (d, $^1J_{H-H}$=9.2Hz, 1H), 3.65 (s, 3H), 3.51–3.48 (m, 2H), 3.45 (s, 3H); ^{13}C NMR (100 MHz, $CDCl_3$): δ=198.7, 168.2, 167.7, 148.4, 146.9, 136.5, 133.4, 129.3, 128.7, 127.9, 123.6, 56.5, 52.8, 52.6, 41.8, 40.3; IR (neat) ν=1727 (CO), 1679 cm^{-1} (CO). MS (70 eV) *m/z* (%): 385 (9) [M]$^+$,294 (12), 254 (47), 105 (100). 88.9% ee, t_R=27.994 min (minor), t_R =46.776 min (major).

(*R*)(-)-Dimethyl 2-(1-(4-chlorophenyl)-3-oxo-3-phenylpropyl)malonate (6c). Grey solid (335 mg, 89% yield); m.p 83–84°C (Lit.[3] 84–86°C); [α]D_{20}= -26.76 (c=0.15 in $CHCl_3$; Lit.[3] 23.9 for S enantiomer); ^{1}H NMR (400 MHz, $CDCl_3$): δ = 7.81 (d, $^1J_{H-H}$=8.0Hz, 2H), 7.48–7.44 (m, 1H), 7.37–7.33 (m, 2H), 7.18–7.14 (m, 4H), 4.12–4.06 (m, 1H), 3.74 (d, $^1J_{H-H}$=9.2Hz, 1H), 3.65 (s, 3H), 3.46 (s, 3H), 3.44–3.33 (m, 2H); ^{13}C NMR (100 MHz, $CDCl_3$): δ=197.2, 168.5, 167.9, 139.0, 136.6, 133.2, 133.0, 129.6, 128.66, 128.63, 57.0, 52.7, 52.5, 42.1, 40.1; IR (neat) ν=1701 (CO), 1664 cm^{-1} (CO). MS (70 eV) *m/z* (%): 374 (4) [M]$^+$,283 (18), 243 (27), 105 (100). 92.9% ee, t_R=10.587 min (minor), t_R =15.164 min (major).

(*R*)(-)-Dimethyl 2-(3-oxo-3-phenylpropyl-1-(4-tolyl))malonate (6d). Pale yellow solid (325 mg, 92% yield); m.p 71–72°C (Lit.[3] 72–73°C); [α]D_{20}= -22.56 (c=0.15 in $CHCl_3$; Lit.[4] -21.0 for R enantiomer); ^{1}H NMR (400 MHz, $CDCl_3$): δ = 7.81 (d, $^1J_{H-H}$=7.2Hz, 2H), 7.46–7.42 (m, 1H), 7.35–7.31 (m, 2H), 7.05 (d, $^1J_{H-H}$=8.4Hz, 2H), 6.96 (d, $^1J_{H-H}$=8.4Hz, 2H), 4.11–4.05 (m, 1H), 3.76 (d, $^1J_{H-H}$=9.2Hz, 1H), 3.64 (s, 3H), 3.48–3.31 (m, 5H), 2.18 (s, 3H); ^{13}C NMR (100 MHz, $CDCl_3$): δ=197.6, 168.8, 168.2, 137.4, 136.8, 136.7, 133.0, 129.2, 128.5, 128.1, 127.9, 57.4, 52.6, 52.4, 42.4, 40.4, 21.0; IR (neat) ν=1743 (CO), 1676 cm^{-1} (CO). MS (70 eV) *m/z* (%): 354 (2) [M]$^+$,261 (19), 243 (47), 105 (100). 90.0% ee, t_R=9.303 min (minor), t_R =16.338 min (major).

(*R*)(-)-Dimethyl 2-(1-(4-cyanophenyl)-3-oxo-3-phenylpropyl)malonate (6e). Pale brown solid (335 mg, 92% yield); m.p 97–98°C; [α]D_{20}= -29.88 (c=0.15 in $CHCl_3$; Lit.[3] 31.3 for S enantiomer); ^{1}H NMR (400 MHz, $CDCl_3$): δ = 7.80 (d, $^1J_{H-H}$=7.2Hz, 2H), 7.49–7.46 (m, 3H), 7.38–7.33 (m, 4H), 4.20–4.14 (m, 1H), 3.78 (d, $^1J_{H-H}$=9.2Hz, 1H), 3.66 (s, 3H), 3.52–3.40 (m, 5H); ^{13}C NMR (100 MHz, $CDCl_3$): δ=196.7, 168.2, 167.7, 146.2, 136.3, 133.4, 132.2, 129.1, 128.7, 128.0, 118.6, 111.2, 56.5, 52.8, 52.6, 41.7, 40.6; IR (neat) ν=2140 (CN), 1739 (CO), 1693 cm^{-1} (CO). MS (70 eV) *m/z* (%): 365 (10) [M]$^+$,274 (12), 234 (45), 105 (100). 90.3% ee, t_R=14.240 min (minor), t_R =20.785 min (major).

(-)-Dimethyl 2-(1-(2-nitrophenyl)-3-oxo-3-phenylpropyl)malonate (6f). Grey solid (298 mg, 77% yield); m.p 93–95°C; [α]D_{20}= -26.24 (c=0.15 in $CHCl_3$); ^{1}H NMR (400 MHz, $CDCl_3$): δ = 7.81 (d, $^1J_{H-H}$=9.2Hz, 2H), 7.72–7.71 (m, 1H), 7.47–7.42 (m, 1H), 7.40–7.39 (m, 4H), 7.35–7.23 (m, 1H), 4.62–4.57 (m, 1H), 4.07 (d, $^1J_{H-H}$=9.2Hz, 1H), 3.68–3.62 (m, 2H), 3.64 (s, 3H), 3.53 (s, 3H); ^{13}C NMR (100 MHz, $CDCl_3$): δ=197.1, 168.6, 168.0, 150.4, 136.4, 135.3, 133.3, 132.6, 129.1, 128.6,

128.1, 128.0, 124.7, 55.4, 52.7, 52.6, 41.1, 40.7; IR (neat) ν=1734 (CO), 1682 cm^{-1} (CO). MS (70 eV) *m/z* (%): 385 (4) $[M]^+$,294 (25), 254 (65), 105 (100). 90.5% ee, t_R=38.603 min (major), t_R =44.249 min (minor). Elemental analysis calcd (%) for $C_{20}H_{19}NO_7$: C 62.33; H 4.97; N 3.63; found: C 62.67; H 5.12; N 3.41.

(-)-Dimethyl 2-(1-(3-nitrophenyl)-3-oxo-3-phenylpropyl)malonate (6g). Grey solid (348 mg, 90% yield); m.p 104–105°C (Lit.[5] 102°C); $[\alpha]D_{20}$= -28.99 (c=0.15 in $CHCl_3$); ^1H NMR (400 MHz, $CDCl_3$): δ = 8. 09 (s, 1H), 7.98 (d, $^1J_{H-H}$=8.4Hz, 1H), 7.82 (d, $^1J_{H-H}$=7.2Hz, 2H), 7.62 (d, $^1J_{H-H}$=9.2Hz, 1H),7.49–7.45 (m, 1H), 7.38–7.34 (m, 3H), 4.27–4.21 (m, 1H), 3.82 (d, $^1J_{H-H}$=9.2Hz, 1H), 3.67 (s, 3H), 3.53–3.47 (m, 5H); ^{13}C NMR (100 MHz, $CDCl_3$): δ=196.7, 168.2, 167.7, 148.3, 142.9, 136.4, 135.2, 133.5, 129.4, 128.7, 128.0, 122.8, 122.4, 56.6, 52.8, 52.6, 41.7, 40.1; IR (neat) ν=1759 (CO), 1671 cm^{-1} (CO). MS (70 eV) *m/z* (%): 385 (12) $[M]^+$,294 (33), 254 (78), 105 (100). 89.9% ee, t_R=41.261 min (minor), t_R =45.663 min (major).

(*R*)(-)-Dimethyl 2-(3-oxo-3-phenyl-1-(thiophen-2-yl)propyl)malonate (6h). Pale orange solid (285 mg, 82% yield); m.p 75–76°C; $[\alpha]D_{20}$= -25.66 (c=0.15 in $CHCl_3$; Lit.[3] 30.5 for S enantiomer); ^1H NMR (400 MHz, $CDCl_3$): δ = 7.83 (d, $^1J_{H-H}$=5.2Hz, 2H), 7.39–7.33 (m, 3H), 7.01 (d, $^1J_{H-H}$=5.2Hz, 1H), 6.83–6.82 (m, 1H), 6.77–6.75 (m,1H), 4.48–4.42 (m, 1H), 3.83 (d, $^1J_{H-H}$=9.2Hz, 1H), 3.66 (s, 3H), 3.50–3.47 (m, 5H); ^{13}C NMR (100 MHz, $CDCl_3$): δ=195.6, 166.8, 166.5, 142.1, 135.1, 131.6, 127.0, 126.9, 126.6, 125.1, 124.3. 55.9, 51.1, 51.0, 41.4, 34.4; IR (neat) ν=1748 (CO), 1667 cm^{-1} (CO). MS (70 eV) *m/z* (%): 346 (10) $[M]^+$,255 (8), 215 (52), 105 (100). 89.6% ee, t_R=12.566 min (minor), t_R =15.389 min (major).

(*R*)(-)-Dimethyl 2-(1-(furan-2-yl)-3-oxo-3-phenylpropyl)malonate (6i). White solid (253 mg, 77% yield); m.p 68–69°C; $[\alpha]D_{20}$= -21.98 (c=0.15 in $CHCl_3$; Lit.[4] -13.0 for R enantiomer); ^1H NMR (400 MHz, $CDCl_3$): δ = 7.85 (d, $^1J_{H-H}$=7.2Hz, 2H) 7.48–7.33 (m, 4H), 6.13–6.12 (m, 1H), 6.02 (d, $^1J_{H-H}$=3.2Hz, 1H), 4.28–4.23 (m, 1H), 3.83 (d, $^1J_{H-H}$=9.2Hz, 1H), 3.63 (s, 3H), 3.54 (s, 3H), 3.52–3.36 (m, 2H); ^{13}C NMR (100 MHz, $CDCl_3$): δ=194.8, 166.0, 165.7, 151.1, 142.5, 139.2, 130.7, 126.2, 125.7, 107.9, 104.6, 52.4, 50.2, 50.1, 37.3, 31.9; IR (neat) ν=1772 (CO), 1645 cm^{-1} (CO). MS (70 eV) *m/z* (%): 330 (4) $[M]^+$,239 (5), 199 (58), 105 (100). 89.3% ee, t_R=11.463 min (minor), t_R =15.750 min (major).

(*R*)(-)-Dimethyl 2-(3-oxo-1-phenylbutyl)malonate (6j). White solid (229 mg, 82% yield); m.p 44–45°C (Lit.[6] 44–46°C); $[\alpha]D_{20}$= -10.25 (c=0.15 in $CHCl_3$; Lit.[7] -13.6 for R enantiomer); ^1H NMR (400 MHz, $CDCl_3$): δ = 7.20–7.09 (m, 5H), 3.92–3.87 (m, 1H), 3.63 (s, 3H), 3.41 (s, 3H), 2.92–2.81 (m, 2H), 1.94 (s,3H); ^{13}C NMR (100 MHz, $CDCl_3$): δ=205.9, 168.5, 168.0, 140.4, 128.5, 127.9, 127.2, 57.1, 52.5, 52.3, 46.9, 40.4, 30.2; IR (neat) ν=1719 (CO), 1631 cm^{-1} (CO). MS (70 eV) *m/z* (%): 278 (10) $[M]^+$,187 (24), 147 (59), 43 (100). 92.5% ee, t_R=8.439 min (minor), t_R =12.477 min (major).

(-)-Dimethyl 2-(3-oxo-1-(*o*-tolyl)butyl)malonate (6k). Waxy solid (262 mg, 90% yield); $[\alpha]D_{20}$= -12.47 (c=0.15 in $CHCl_3$); ^1H NMR (400 MHz, $CDCl_3$): δ = 7.04–6.97 (m, 4H), 4.21–4.15 (m, 1H), 3.65–3.63 (m, 4H), 3.38 (s, 3H), 2.86–2.82 (m, 2H), 2.38 (s, 3H), 1.90 (s,3H); ^{13}C NMR (100 MHz, $CDCl_3$): δ=205.9, 168.7, 168.1, 140.8, 139.0, 136.8, 130.7, 126.8, 126.1, 56.7, 52.6, 52.2, 47.4, 35.2, 30.4, 19.7; IR (neat) ν=1751 (CO), 1625 cm^{-1} (CO). MS (70 eV) *m/z* (%): 292 (4) $[M]^+$,201 (16), 161 (34), 43 (100). 94.4% ee, t_R=10.085 min (minor), t_R =13.935 min (major).

(-)-Dimethyl 2-(1-(3-methoxyphenyl)-3-oxo-butyl)malonate (6l). Waxy solid (262 mg, 85% yield); $[\alpha]D_{20}$= -18.91 (c=0.15 in $CHCl_3$); ^1H NMR (400 MHz, $CDCl_3$): δ = 7.12–7.10 (m, 2H), 6.74–6.65 (m, 2H), 3.90–3.84 (m, 1H), 3.69 (s, 3H), 3.67–3.66 (m, 1H), 3.64 (s, 3H), 3.45 (s, 3H), 2.91–2.79 (m, 2H), 1.96 (s,3H); ^{13}C NMR (100 MHz, $CDCl_3$): δ=205.9, 168.5, 168.0, 159.6, 142.0, 129.5, 120.0, 113.9, 112.5, 57.0, 55.1, 52.6, 52.3, 47.0, 40.4, 30.3; IR (neat) ν=1720 (CO), 1648 cm^{-1} (CO). MS

(70 eV) *m/z* (%): 308 (11) $[M]^+$,217 (19), 177 (49), 43 (100). 88.8% ee, t_R=11.127 min (minor), t_R =14.863 min (major).

(*R*)(-)-Dimethyl 2-(4-oxo-4-phenylbutan-2-yl)malonate (6m). Waxy solid (241 mg, 87% yield); [α]D_{20}= -5.87 (c=0.15 in $CHCl_3$; Lit.[3] 4.8 for S enantiomer); ^{1}H NMR (400 MHz, $CDCl_3$): δ = 7.88 (d, $^1J_{H-H}$=8.0Hz, 2H) 7.49–7.46 (m, 1H), 7.39–7.36 (m, 1H), 3.66 (s, 3H), 3.64 (s, 3H), 3.45 (d, $^1J_{H-H}$=6.4Hz, 1H), 3.21–3.16 (m, 1H), 2.93–2.84 (m, 2H), 1.00 (d, $^1J_{H-H}$=6.4Hz, 3H); ^{13}C NMR (100 MHz, $CDCl_3$): δ=198.7, 169.1,169.0, 136.9, 133.1, 128.6, 128.1, 56.1, 52.4, 52.3, 42.6, 29.6, 17.8; IR (neat) ν=1735 (CO), 1651 cm^{-1} (CO). MS (70 eV) *m/z* (%): 278 (5) $[M]^+$,147 (35), 105 (100), 89.5% ee, t_R=8.946 min (minor), t_R =10.423 min (major).

(-)-Dimethyl 2-(3-oxo-1-phenyl-3-(4-(trifluoromethyl)phenyl)propyl)malonate (6n). Pale yellow solid (375 mg, 92% yield); m.p. 114–115°C; [α]D_{20}= -16.78 (c=0.15 in $CHCl_3$); ^{1}H NMR (400 MHz, $CDCl_3$): δ = 7.94–7.81 (m, 2H), 7.61–7.57 (m, 2H), 7.19–7.07 (m, 5H), 4.12–4.06 (m, 1H), 3.77 (d, $^1J_{H-H}$=9.2Hz, 1H), 3.69 (s, 3H), 3.64 (s, 3H), 3.55–3.41 (m, 2H); ^{13}C NMR (100 MHz, $CDCl_3$): δ=195.3, 167.3, 166.7, 141.8, 138.7, 132.9 (q, $^2J_{C-F}$=32.5 Hz), 127.3, 127.1, 126.6, 124.3 (q, $^3J_{C-F}$=3.6 Hz), 122.1 (q, $^1J_{C-F}$=271,2 Hz), 55.7, 51.2, 41.3, 39.4; IR (neat) ν=1754 (CO), 1656 cm^{-1} (CO). MS (70 eV) *m/z* (%): 408 (7) $[M]^+$,317 (23), 277 (67), 173 (100). 89.7% ee, t_R=10.657 min (minor), t_R =14.139 min (major). Elemental analysis calcd (%) for $C_{21}H_{19}F_3O_5$: C 61.76; H 4.69; found: C 61.02; H 4.77.

(-)-Dimethyl 2-(3-(2-methoxyphenyl)-3-oxo-1-phenylpropyl)malonate (6o). Waxy solid (291 mg, 79% yield); [α]D_{20}= -22.69 (c=0.15 in $CHCl_3$); ^{1}H NMR (400 MHz, $CDCl_3$): δ = 7.38–7.31 (m, 3H), 7.29–7.27 (m, 4H), 7.13–6.77 (m, 2H), 4.06–4.00 (m, 1H), 3.78–3.71 (m, 5H), 3.63 (s, 3H), 3.42–3.37 (m, 4H); ^{13}C NMR (100 MHz, $CDCl_3$): δ=199.8, 168.6, 168.2, 158.3, 140.8, 133.4, 130.2, 128.9, 128.3, 126.9, 125.5 120.5, 111.4, 57.6, 55.4, 52.5, 52.3, 47.5, 40.9; IR (neat) ν=1781 (CO), 1675 cm^{-1} (CO). MS (70 eV) *m/z* (%): 370 (12) $[M]^+$,279 (13), 239 (67), 151 (100). 87.0% ee, t_R=8.743 min (minor), t_R =10.620 min (major). Elemental analysis calcd (%) for $C_{21}H_{22}O_6$: C 68.10; H 5.99; found: C 68.67; H 5.81.

(-)-Dimethyl 2-(1-(4-chlorophenyl)-3-oxo-3-(*m*-tolyl)propyl)malonate (6p). Grey solid (320 mg, 82% yield); m.p 159–160°C; [α]D_{20}= -15.88 (c=0.15 in $CHCl_3$); ^{1}H NMR (400 MHz, $CDCl_3$): δ = 7.59–7.57 (m, 2H), 7.23–7.17 (m, 2H), 7.13–7.09 (m, 4H), 4.11–4.06 (m, 1H), 3.77 (d, $^1J_{H-H}$=9.2Hz, 1H), 3.62 (s, 3H), 3.44–3.32 (m, 5H), 2.25 (s,3H); ^{13}C NMR (100 MHz, $CDCl_3$): δ=197.3, 168.5, 167.9, 139.1, 138.4, 136.7, 133.9, 132.9, 129.5, 128.6, 128.5 128.4, 125.3, 57.0, 52.7, 52.4, 42.1, 41.1, 40.1; IR (neat) ν=1741 (CO), 1665 cm^{-1} (CO). MS (70 eV) *m/z* (%): 388 (16) $[M]^+$,297 (21), 257 (42), 135 (100). 89.9% ee, t_R=41.261 min (minor), t_R =45.504 min (major). Elemental analysis calcd (%) for $C_{21}H_{21}ClO_5$: C 64.87; H 5.44; found: C 65.11; H 5.77.

(-)-Dimethyl 2-(1-(4-nitrophenyl)-3-oxo-3-(*m*-tolyl)propyl)malonate (6q). Pale brown waxy solid (364 mg, 91% yield); [α]D_{20}= -17.12 (c=0.15 in $CHCl_3$); ^{1}H NMR (400 MHz, $CDCl_3$): δ = 8.03 (d, $^1J_{H-H}$=8.4Hz, 2H), 7.61–7.59 (m, 2H), 7.41 (d, $^1J_{H-H}$=8.4Hz, 2H), 7.28–7.20 (m, 2H), 4.26–4.20 (m, 1H), 3.82 (d, $^1J_{H-H}$=9.2Hz, 1H), 3.67 (s, 3H), 3.49–3.36 (m, 5H), 2.29 (s, 3H); ^{13}C NMR (100 MHz, $CDCl_3$): δ=196.8, 168.1, 167.7, 148.4, 146.9, 138.5, 136.3, 134.2, 129.3, 128.6, 128.5, 125.2, 123.6, 56.5, 52.8, 52.7, 41.8, 40.3, 21.2; IR (neat) ν=1727 (CO), 1676 cm^{-1} (CO). MS (70 eV) *m/z* (%): 399 (12) $[M]^+$,308 (33), 268 (55), 120 (100). 89.7% ee, t_R=39.208 min (minor), t_R =50.359 min (major). Elemental analysis calcd (%) for $C_{21}H_{21}NO_7$: C 63.15; H 5.30; found: C 63.87; H 5.82.

(-)-Dimethyl 2-(3-oxo-1-(*p*-tolyl)-3-(4-trifluoromethyl)phenyl)propyl)malonate (6r). Grey waxy solid (380 mg, 90% yield); [α]D_{20}= -23.18 (c=0.15 in $CHCl_3$); ^{1}H NMR (400 MHz, $CDCl_3$): δ =

7.91 (d, $^1J_{H\text{-}H}$=8.4Hz, 2H), 7.60 (d, $^1J_{H\text{-}H}$=8.4Hz, 2H), 7.03 (d, $^1J_{H\text{-}H}$=8.4Hz, 2H), 6.97 (d, $^1J_{H\text{-}H}$=8.4Hz, 2H) 4.08–4.02 (m, 1H), 3.75 (d, $^1J_{H\text{-}H}$=9.2Hz, 1H), 3.66 (s, 3H), 3.53–3.31 (m, 5H), 2.18 (s, 3H); ^{13}C NMR (100 MHz, $CDCl_3$): δ=196.9, 168.7, 168.1, 139.5, 136.9, 134.2 (q, $^2J_{C\text{-}F}$=32.5 Hz), 129.2, 128.4, 127.8, 125.5 (q, $^3J_{C\text{-}F}$=3.6 Hz), 122.4 (q, $^1J_{C\text{-}F}$=271.2 Hz), 57.2, 52.6, 52.4, 42.7, 40.4, 21.0; IR (neat) ν=1766 (CO), 1615 cm^{-1} (CO). 92.4% ee, t_R=10.004 min (minor), t_R =12.814 min (major). Elemental analysis calcd (%) for $C_{22}H_{21}F_3O_5$: C 62.56; H 5.01; found: C 63.04; H 4.77.

(*R*)(-)-Dimethyl 2-(3-(4-chlorophenyl)-3-oxo-1-phenylpropyl)malonate (6s). White solid (322 mg, 85% yield); m.p. 65–66°C; (Lit.[3] 67–68°C); [α]D_{20}= -21.23 (c=0.15 in $CHCl_3$; Lit.[3] 20.9 for S enantiomer); ^{1}H NMR (400 MHz, $CDCl_3$): δ =7.75 (d, $^1J_{H\text{-}H}$=8.8Hz, 2H), 7.31 (d, $^1J_{H\text{-}H}$=8.8Hz, 2H), 7.18–7.07 (m, 5H), 4.11–4.05 (m, 1H), 3.77 (d, $^1J_{H\text{-}H}$=9.2Hz, 1H), 3.64 (s, 3H), 3.48–3.31 (m, 5H); ^{13}C NMR (100 MHz, $CDCl_3$): δ=196.4, 168.7, 168.0, 140.2, 139.5, 135.1, 129.5, 128.9, 128.5, 128.0, 127.3, 57.2, 52.6, 52.4, 42.3, 40.8; IR (neat) ν=1767 (CO), 1656 cm^{-1} (CO). MS (70 eV) *m/z* (%): 376 (5) $[M]^+$,284 (42), 244 (67), 140 (100). 89.0% ee, t_R=8.338 min (major), t_R =14.911 min (minor).

(*R*)(-)-Dimethyl 2-(3-(4-nitrophenyl)-3-oxo-1-phenylpropyl)malonate (6t). White solid (328 mg, 85% yield); m.p. 70–71°C; [α]D_{20}= -20.77 (c=0.15 in $CHCl_3$) (Lit.[3] 19.5 for S enantiomer D); ^{1}H NMR (400 MHz, $CDCl_3$): δ = 8.18 (d, $^1J_{H\text{-}H}$=9.2Hz, 2H), 7.95 (d, $^1J_{H\text{-}H}$=8.8Hz, 2H), 7.19–7.09 (m, 5H), 4.10–4.05 (m, 1H), 3.77 (d, $^1J_{H\text{-}H}$=9.2Hz, 1H), 3.65 (s, 3H), 3.45–3.35 (m, 5H); ^{13}C NMR (100 MHz, $CDCl_3$): δ=196.3, 168.7, 168.0, 150.3, 141.1, 139.8, 129.1, 128.6, 127.9, 127.5, 123.8, 57.0, 52.7, 52.5, 42.8, 40.7; IR (neat) ν=1754 (CO), 1666 cm^{-1} (CO). MS (70 eV) *m/z* (%): 386 (2) $[M]^+$,294 (33), 254 (48), 150 (100). 90.5% ee, t_R=38.303 min (major), t_R =44.249 min (minor).

(-)-Dimethyl 2-(4-oxohexan-2-yl)malonate (6u). Viscous pale yellow liquid (195 mg, 85% yield); [α]D_{20}= -6.35 (c=0.15 in $CHCl_3$); ^{1}H NMR (400 MHz, $CDCl_3$): δ = 3.66 (s, 3H), 3.65 (s, 3H), 3.35 (d, $^1J_{H\text{-}H}$=6.8Hz, 1H), 2.77–2.67 (m, 1H), 2.57 (dd, $^1J_{H\text{-}H}$=17.2Hz, $^2J_{H\text{-}H}$=4.8Hz, 1H), 2.37–2.31 (m, 3H), 0.99–0.94 (m, 6H) ; ^{13}C NMR (100 MHz, $CDCl_3$): δ=209.8, 169.0, 168.9, 55.8, 52.3, 52.2, 46.1, 36.2, 29.0, 17.7, 7.7; IR (neat) ν=1732 (CO), 1669 cm^{-1} (CO). MS (70 eV) *m/z* (%): 230 (2) $[M]^+$, 57 (100). 81.5% ee, t_R=10.546 min (major), t_R =11.717 min (minor). Elemental analysis calcd (%) for $C_{11}H_{18}O_5$: C 57.38; H 7.88; found: C 57.61; H 7.25.

***(R)(+)*-Dimethyl 2-(3-oxocyclopentyl)malonate (6v).** Viscous oil (177 mg, 83% yield); [α]D_{20}= 67.5 (c=0.15 in $CHCl_3$; Lit.[8] 81.7 for R enantiomer); ^{1}H NMR (400 MHz, $CDCl_3$): δ = 3.70 (s, 3H), 3.66 (s, 3H), 3.32 (d, $^1J_{H\text{-}H}$=9.2 Hz, 1H), 2.83–2.73 (m, 1H), 2.46–2.40 (m, 1H), 2.31–2.11 (m, 3H), 1.98–1.90 (m,1H), 1.64–1.57 (m,1H); ^{13}C NMR (100 MHz, $CDCl_3$): δ=209.4, 168.2, 168.1, 56.0, 52.7, 52.6, 42.8, 38.1, 36.3, 27.4 ; IR (neat) ν=1729 (CO), 1656 cm^{-1} (CO). MS (70 eV) *m/z* (%): 214 (5) $[M]^+$, 96 (100). 87.5% ee, t_R=8.884 min (minor), t_R =10.370 min (major).

***(R)(+)*-Dimethyl 2-(3-oxocyclohexyl)malonate (6w).** White solid (208 mg, 91% yield); m.p 56–57°C (Lit.[9] 56°C); [α]D_{20}= 1.10 (c=0.15 in $CHCl_3$; Lit.[8] 2.6 for R enantiomer) K; ^{1}H NMR (400 MHz, $CDCl_3$): δ = 3.68 (s, 3H), 3.67 (s, 3H), 3.30 (d, $^1J_{H\text{-}H}$=8.0 Hz, 1H), 2.49–2.41 (m, 1H), 2.38–2.31 (m, 2H), 2.23–2.15 (m, 2H), 2.05–1.97 (m,1H), 1.90–1.84 (m,1H), 1.67–1.55 (m,1H), 1.48–1.38 (m,1H) ; ^{13}C NMR (100 MHz, $CDCl_3$): δ=216.8, 168.5, 168.4, 56.5, 52.5, 45.0, 41.0, 40.9, 38.0, 28.7, 24.4 ; IR (neat) ν=1738 (CO), 1664 cm^{-1} (CO). MS (70 eV) *m/z* (%): 228 (5) $[M]^+$, 97 (100). 70.2% ee, t_R=8.994 min (major), t_R =9.495 min (minor).

References

1. Capreti, N. M. R.; Jurberg, I.D. Michael addition of soft carbon nucleophiles to alkylidene isoxazol-5-ones: a divergent entry to β-branched carbonyl compounds. *Org.Lett.*, **2015**, *17*, 2490−2493. DOI: 10.1021/acs.orglett.5b01004
2. Espinosa, M.; Blay, G.; Cardona, L.; Pedro, J. R. Asymmetric conjugate addition of malonate esters to α,β-unsaturated *N*-sulfonyl imines: an expeditious route to chiral δ-aminoesters and piperidones. *Chem. Eur. J.*, **2013**,*19*, 14861–14866. DOI: 10.1002/chem.201302687.
3. Cao, D.; Fang, G.; Zhang, J.; Wang, H.; Zheng, C.; Zhao, G. Enantioselective Michael addition of malonates to chalcone derivatives catalyzed by dipeptide-derived multifunctional phosphonium salts. *J. Org. Chem.*, **2016**, *81*, 9973–9982. DOI: 10.1021/acs.joc.6b01752
4. Mao, Z.; Jia, Y.; Li, W.; Wang, R. Water-compatible iminium activation: highly enantioselective organocatalytic Michael addition of malonates to α,β-unsaturated enones. *J. Org. Chem*., **2010**, *75*, 7428–7430. DOI: 10.1021/jo101188m.
5. Kohler, E. P.; Hill, G. A.; Bigelow, L. A. Studies in the cyclopropane series. Third paper. *J. Am. Chem. Soc*., **1917**, *39*, 2405–2418. DOI: 10.1021/ja02256a020.
6. De Simone, N. A.; Meninno, S.; Talotta, C.; Gaeta, C.; Neri, P.; Lattanzi, A. Solvent-free enantioselective Michael reactions catalyzed by a calixarene-based primary amine thiourea. *J. Org. Chem*., **2018**, *83*, 10318–10325. DOI: 10.1021/acs.joc.8b01454.
7. Ueda, A.; Umeno, T.; Doi, M.; Agakawa, K.; Kudo, K.; Tanaka, M. Helical-peptide-catalyzed enantioselective Michael addition reactions and their mechanistic insights. *J. Org. Chem.* **2016**, *81*, 6343–6356. DOI: 10.1021/acs.joc.6b00982.
8. Dudzinski, K.; Pakulska, A. M.; Kwiatkowski, P. An efficient organocatalytic method for highly enantioselective Michael addition of malonates to enones catalyzed by readily accessible primary amine-thiourea. *Org. Lett.* **2012**, *14*, 4222–4225. DOI: 10.1021/ol3019055.
9. Jiricek, J.; Blechert, S. Enantioselective synthesis of (--)-gilbertine via a cationic cascade cyclization. *J. Am. Chem. Soc*. **2004**, *126*, 3534–3538. DOI: 10.1021/ja0399021

NMR Spectra and chiral analyses of malonates 6

(*R*)-Dimethyl 2-(3-oxo-1,3-diphenylpropyl)malonate (6a)

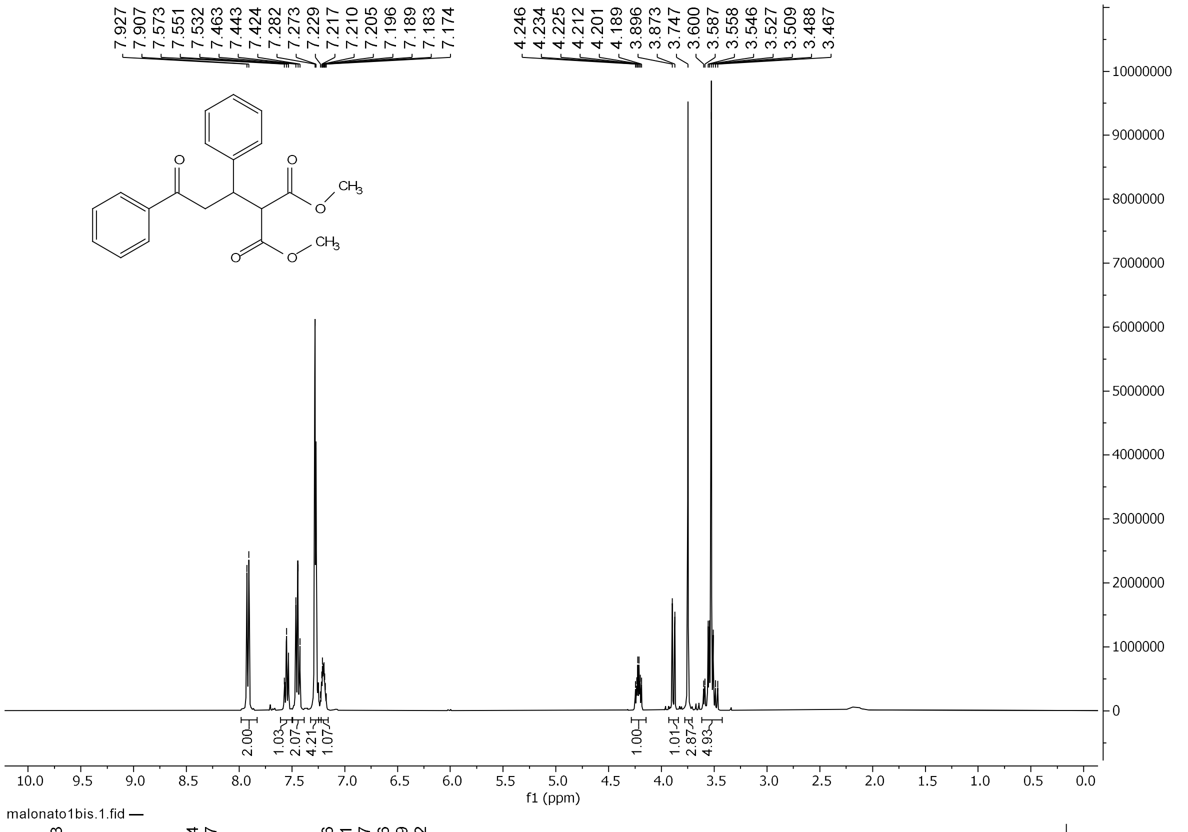

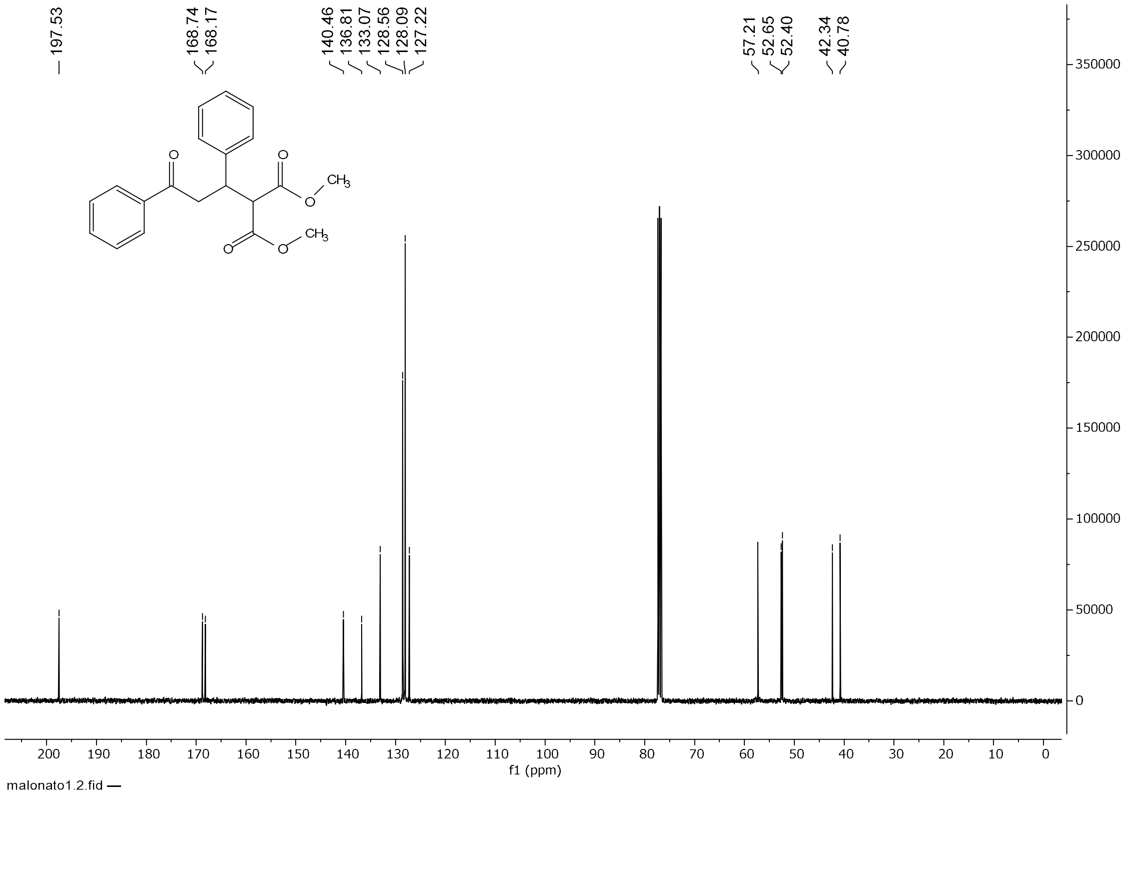

(*rac*)-Dimethyl 2-(3-oxo-1,3-diphenylpropyl)malonate (6a)

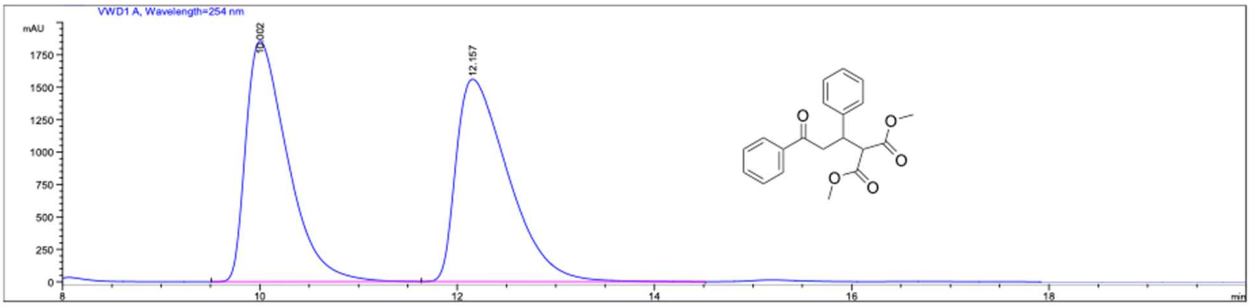

	Ret. Time (min)	Peak Area (mAU *min)	Peak Height (mAu)	Area %
1	10.002	25770.3	1855.5	49.273
2	12.157	26530.4	1551.7	50.727

(*R*)-Dimethyl 2-(3-oxo-1,3-diphenylpropyl)malonate (6a)

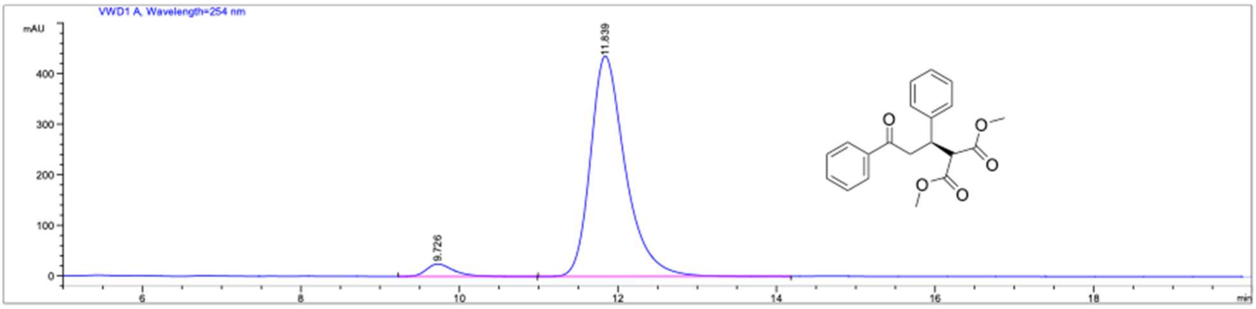

	Ret. Time (min)	Peak Area (mAU *min)	Peak Height (mAu)	Area %
1	9.726	591.1	24.6	4.247
2	11.839	13327.1	435.7	95.753

Eluent: Hexane/ 2-propanol 3:2; 1 mL/min

(*R*)-Dimethyl 2-(1-(4-nitrophenyl)-3-oxo-3-phenylpropyl)malonate (6b)

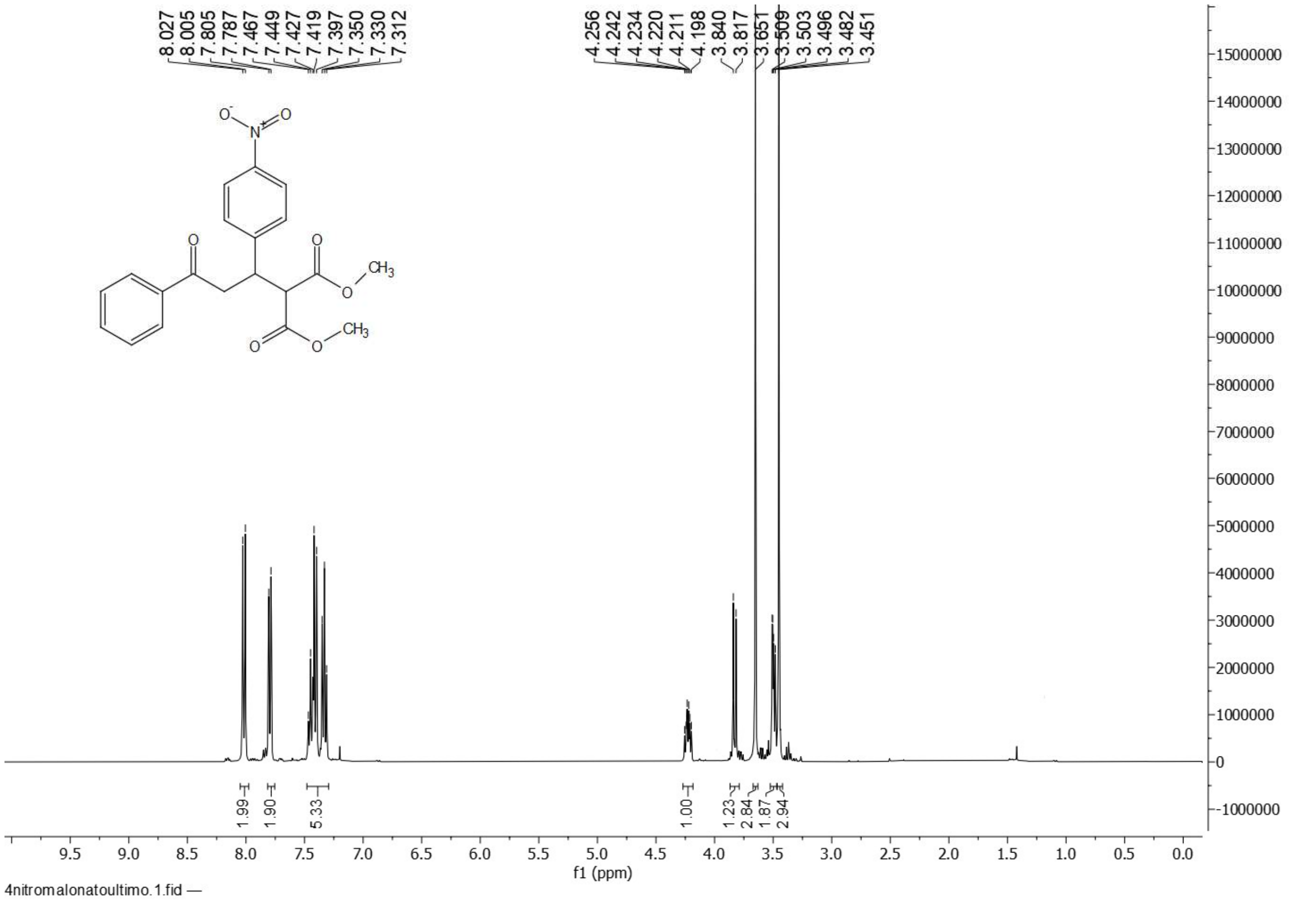

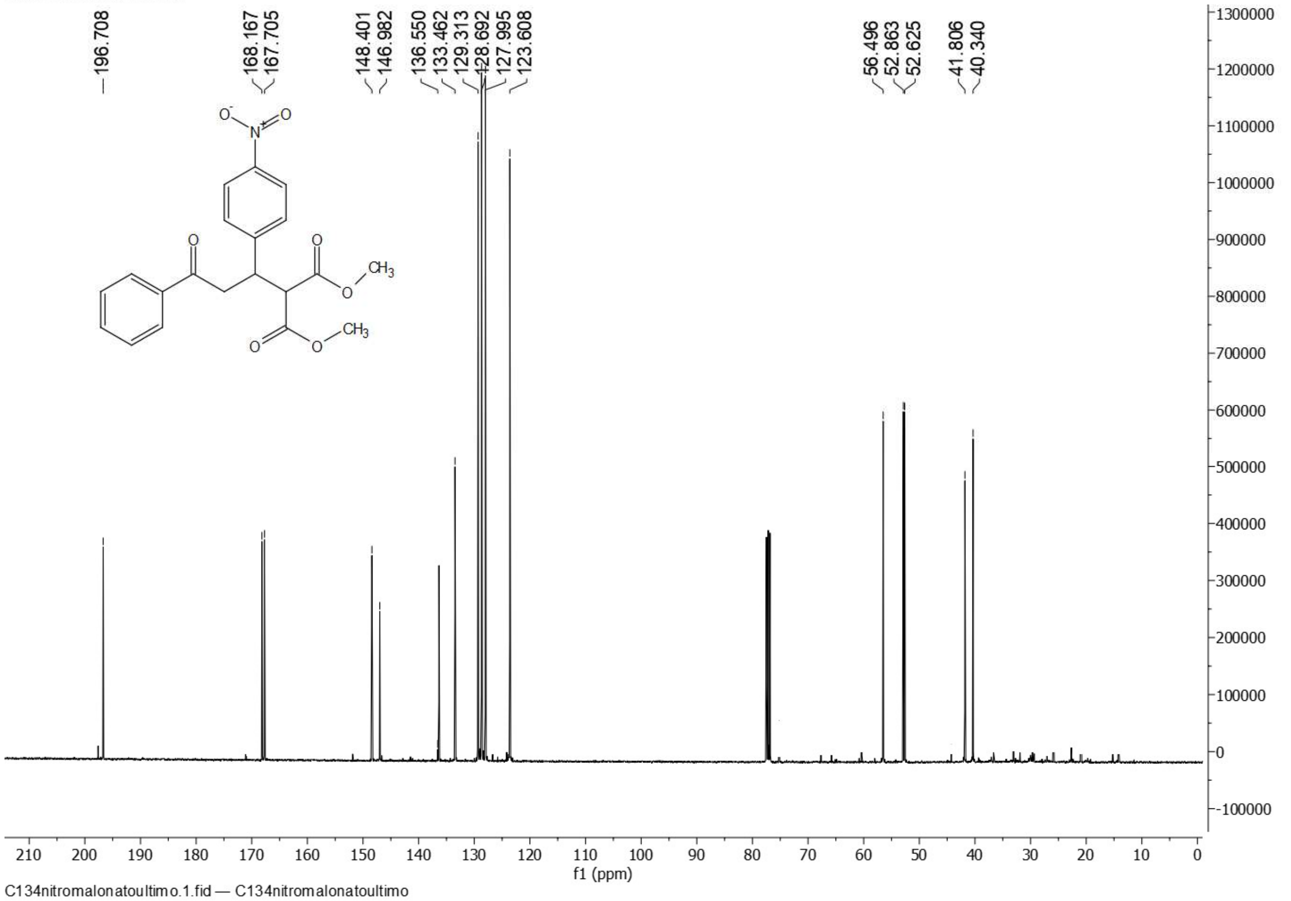

(*rac*)-Dimethyl 2-(1-(4-nitrophenyl)-3-oxo-3-phenylpropyl)malonate (6b)

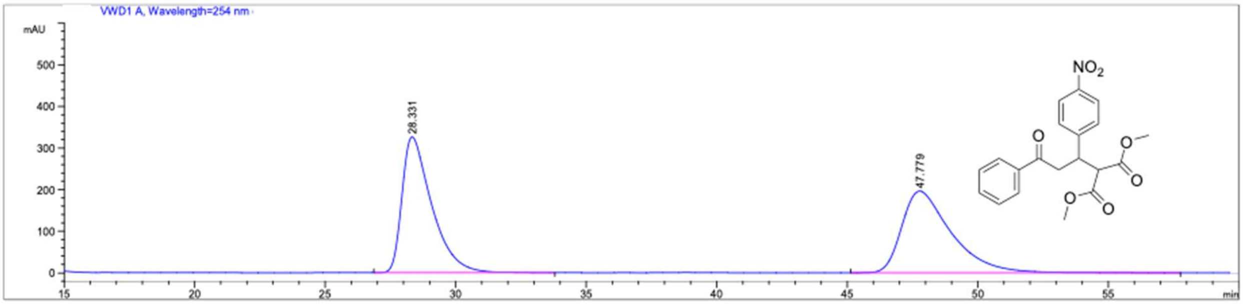

	Ret. Time (min)	Peak Area (mAU *min)	Peak Height (mAu)	Area %
1	28.331	25826.0	326.3	49.939
2	47.779	25889.2	196.3	50.061

(*R*)-Dimethyl 2-(1-(4-nitrophenyl)-3-oxo-3-phenylpropyl)malonate (6b)

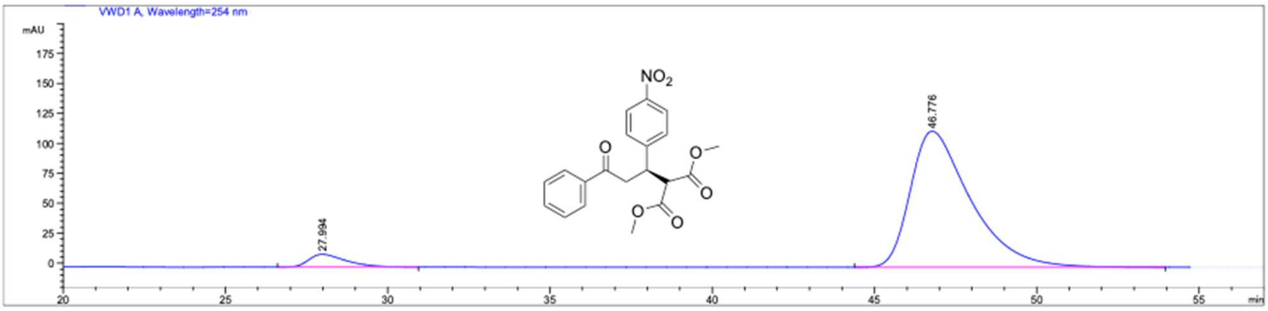

	Ret. Time (min)	Peak Area (mAU *min)	Peak Height (mAu)	Area %
1	27.994	858.6	10.7	5.529
2	46.776	14671.3	113.6	94.471

Eluent: Hexane/ 2-propanol 3:2; 0.8 mL/min

(*R*)-Dimethyl 2-(1-(4-chlorophenyl)-3-oxo-3-phenylpropyl)malonate (6c)

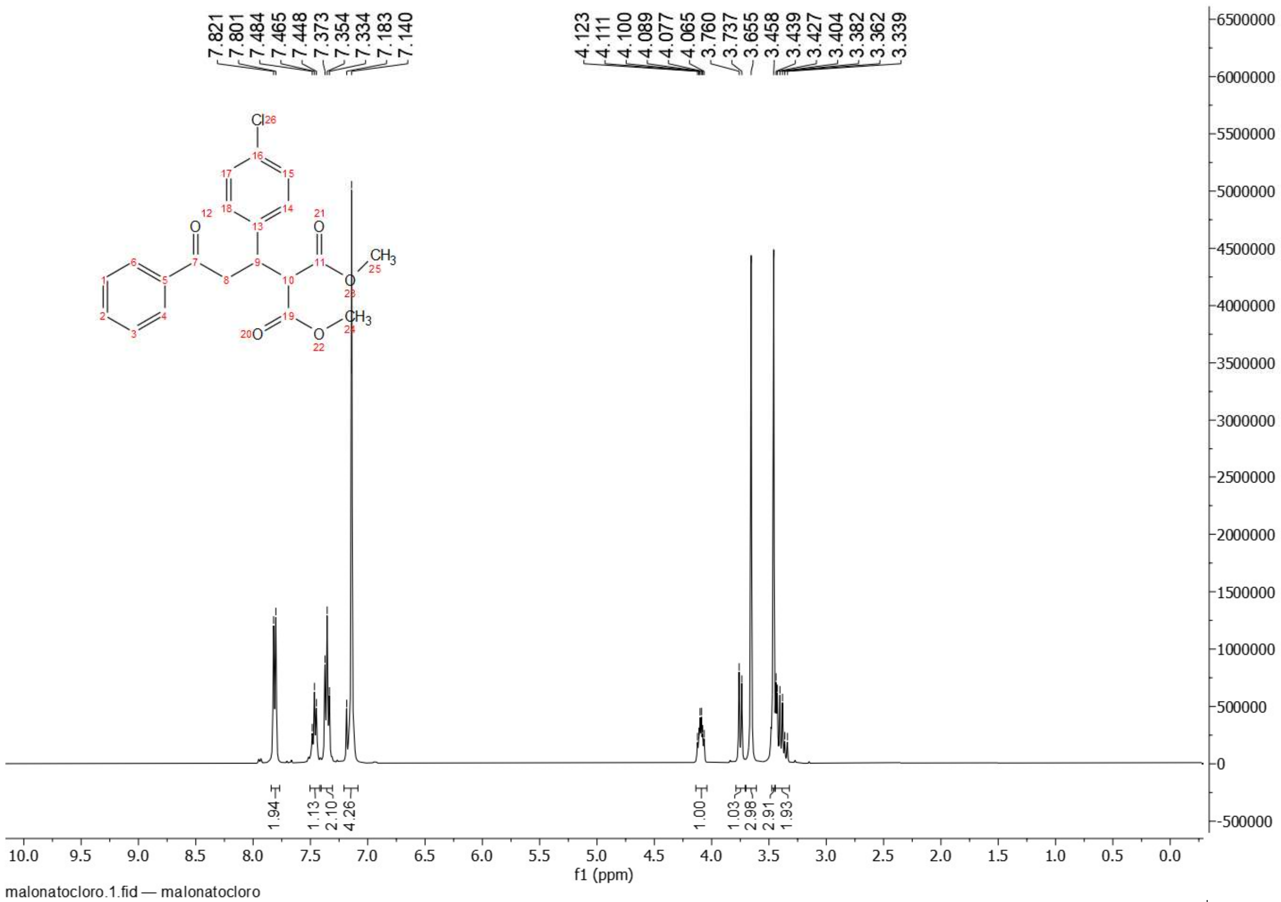

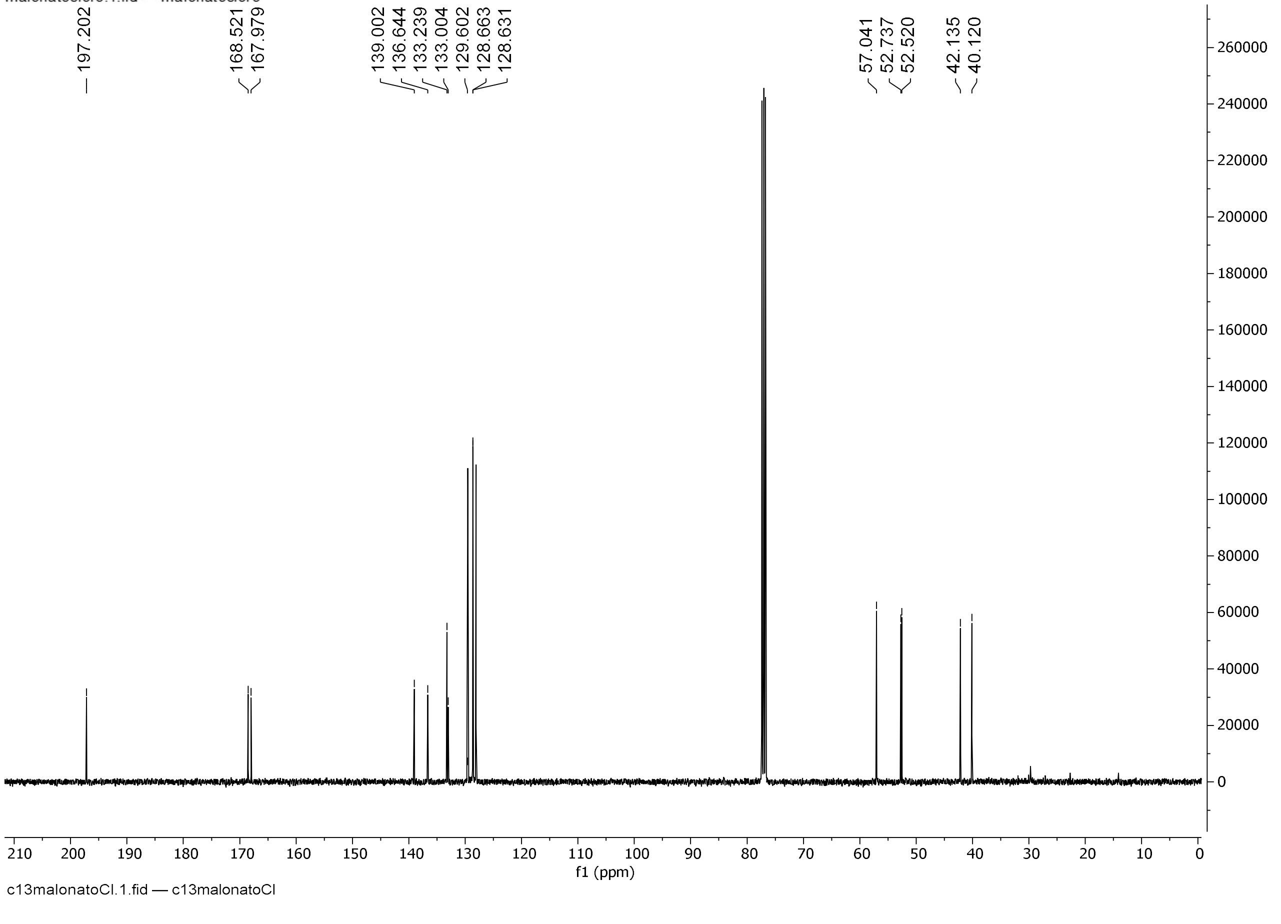

(*rac*)-Dimethyl 2-(1-(4-chlorophenyl)-3-oxo-3-phenylpropyl)malonate (6c)

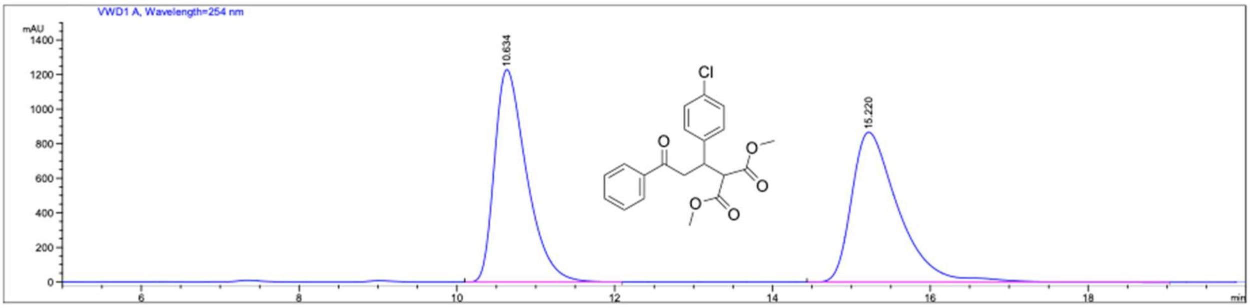

	Ret. Time (min)	Peak Area (mAU *min)	Peak Height (mAu)	Area %
1	10.634	13915.7	1386.3	50.193
2	15.220	13808.8	936.3	49.807

(*R*)-Dimethyl 2-(1-(4-chlorophenyl)-3-oxo-3-phenylpropyl)malonate (6c)

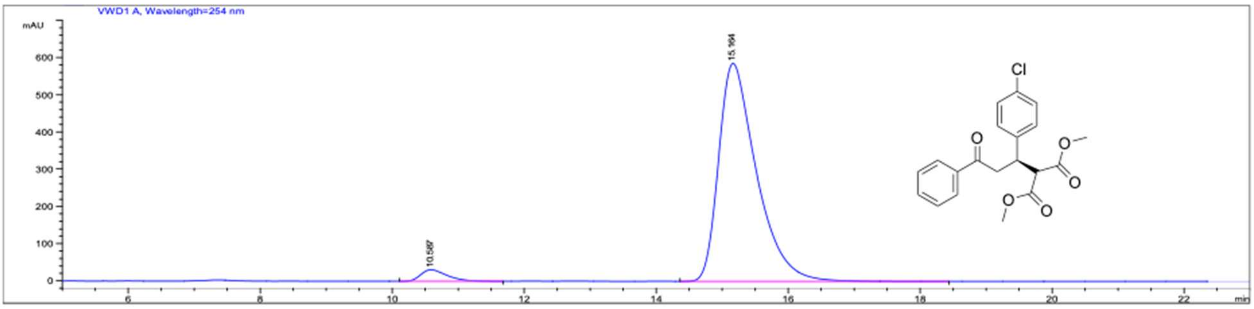

	Ret. Time (min)	Peak Area (mAU *min)	Peak Height (mAu)	Area %
1	10.587	857.3	31.8	3.547
2	15.164	23315.1	586.9	96.453

Eluent: Hexane/ 2-propanol 3:2; 1 mL/min

(*R*)-Dimethyl 2-(1-(4-tolyl)-3-oxo-3-phenylpropyl)malonate (6d)

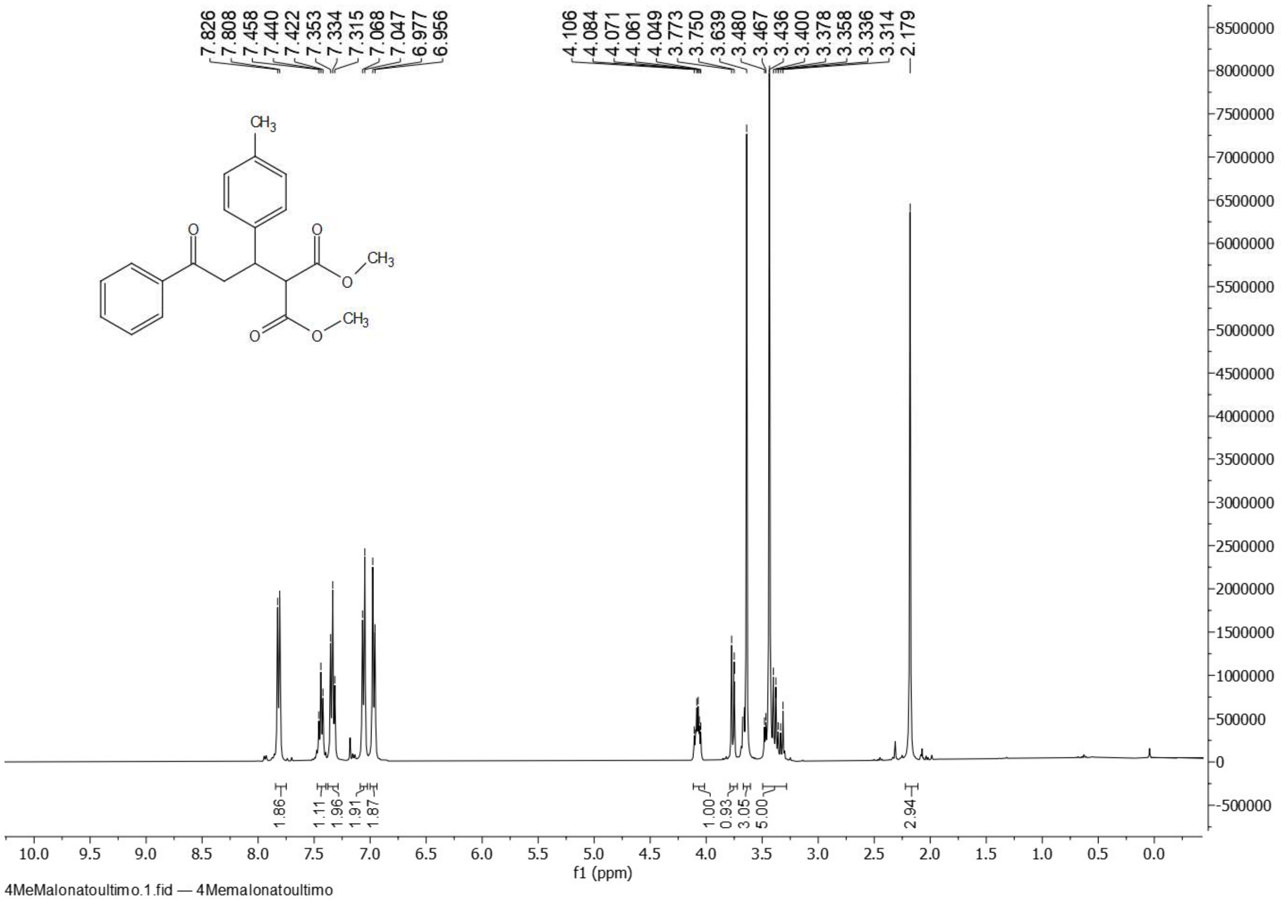

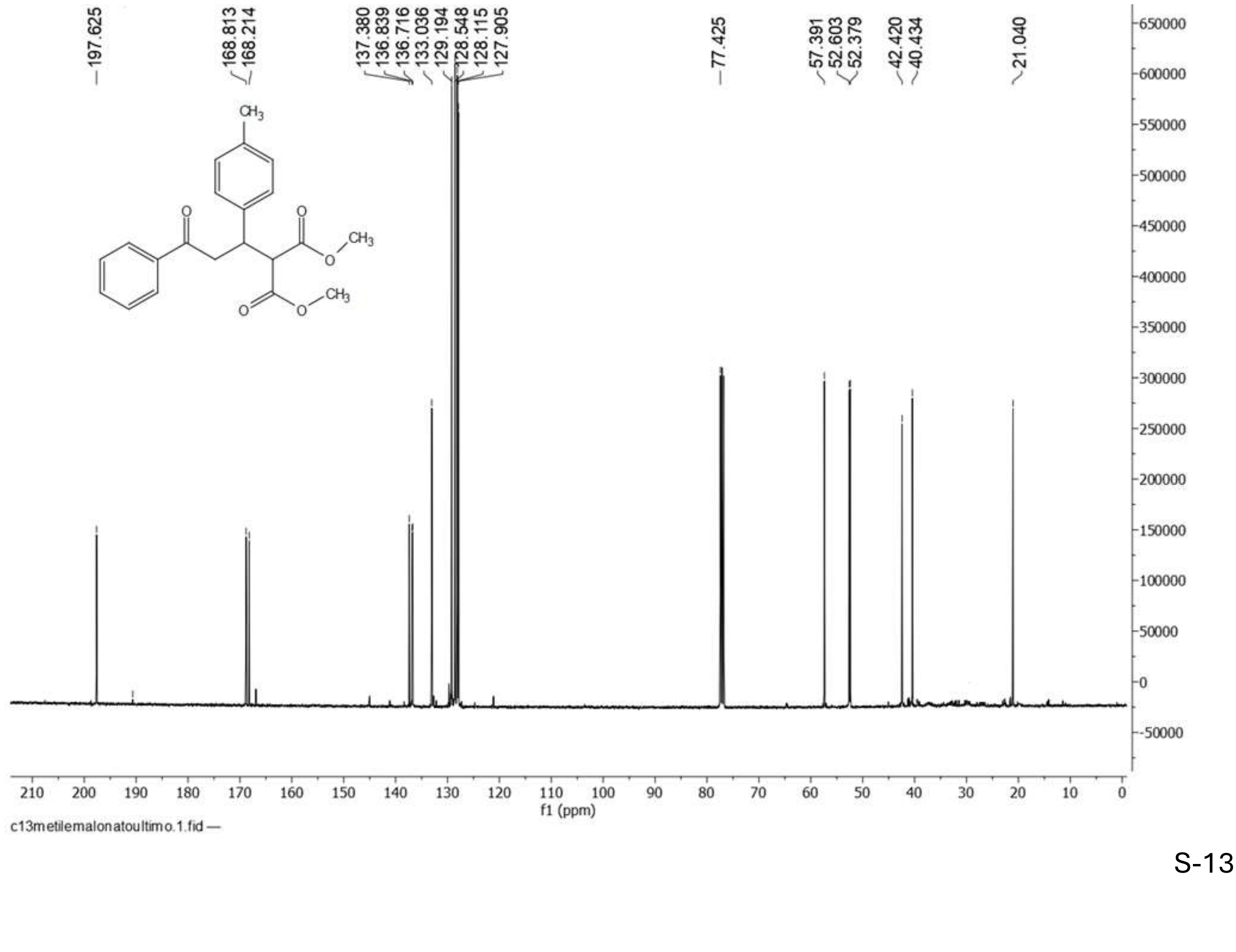

(*rac*)-Dimethyl 2-(1-(4-tolyl)-3-oxo-3-phenylpropyl)malonate (6d)

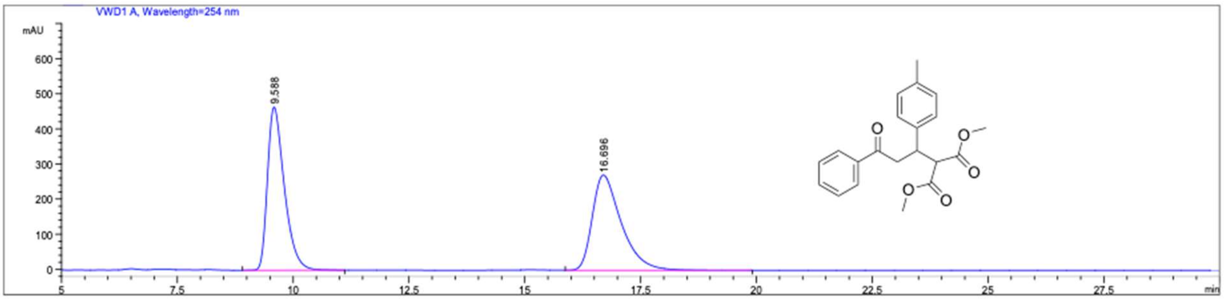

	Ret. Time (min)	Peak Area (mAU *min)	Peak Height (mAu)	Area %
1	9.588	11748.6	464.5	49.948
2	16.696	11773.2	271.1	50.052

(*R*)-Dimethyl 2-(1-(4-tolyl)-3-oxo-3-phenylpropyl)malonate (6d)

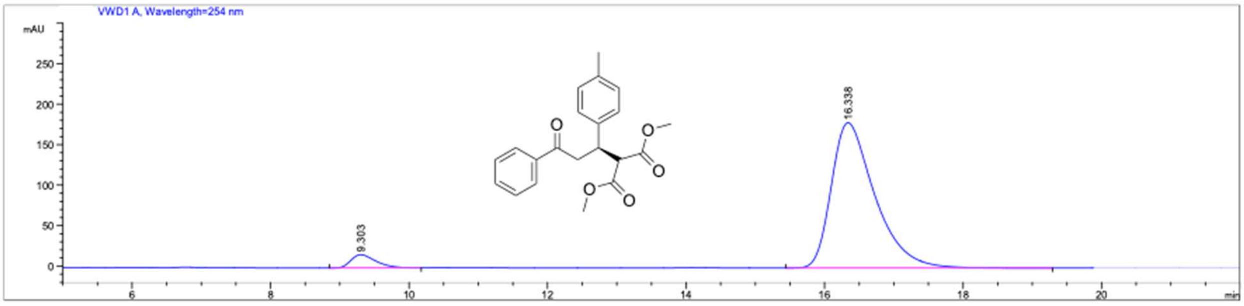

	Ret. Time (min)	Peak Area (mAU *min)	Peak Height (mAu)	Area %
1	9.303	410.9	16.4	4.985
2	16.338	7832.8	179.4	95.015

Eluent: Hexane/ 2-propanol 3:2; 1 mL/min

(*R*)-Dimethyl 2-(1-(4-cyanophenyl)-3-oxo-3-phenylpropyl)malonate (6e)

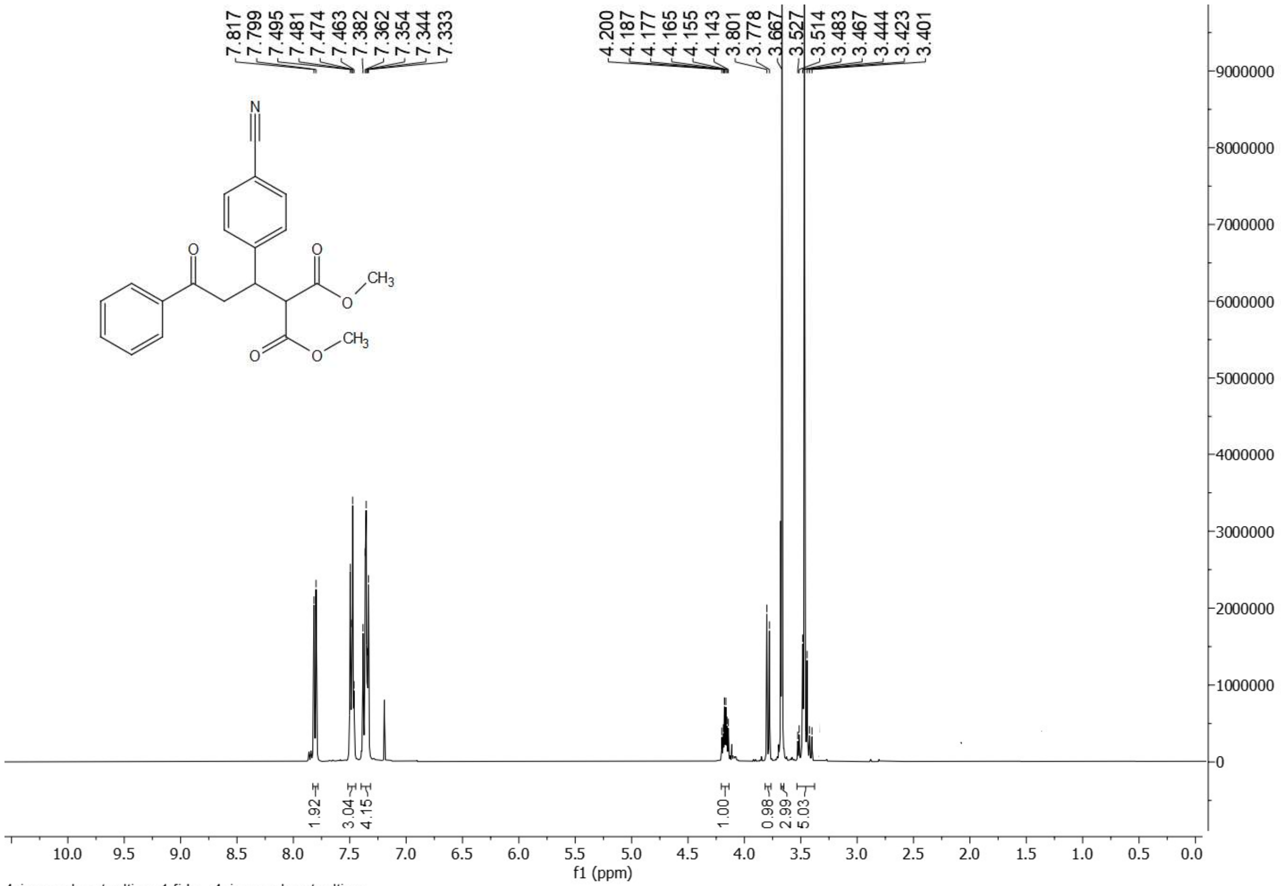

4cianomalonatoultimo.1.fid — 4cianomalonatoultimo

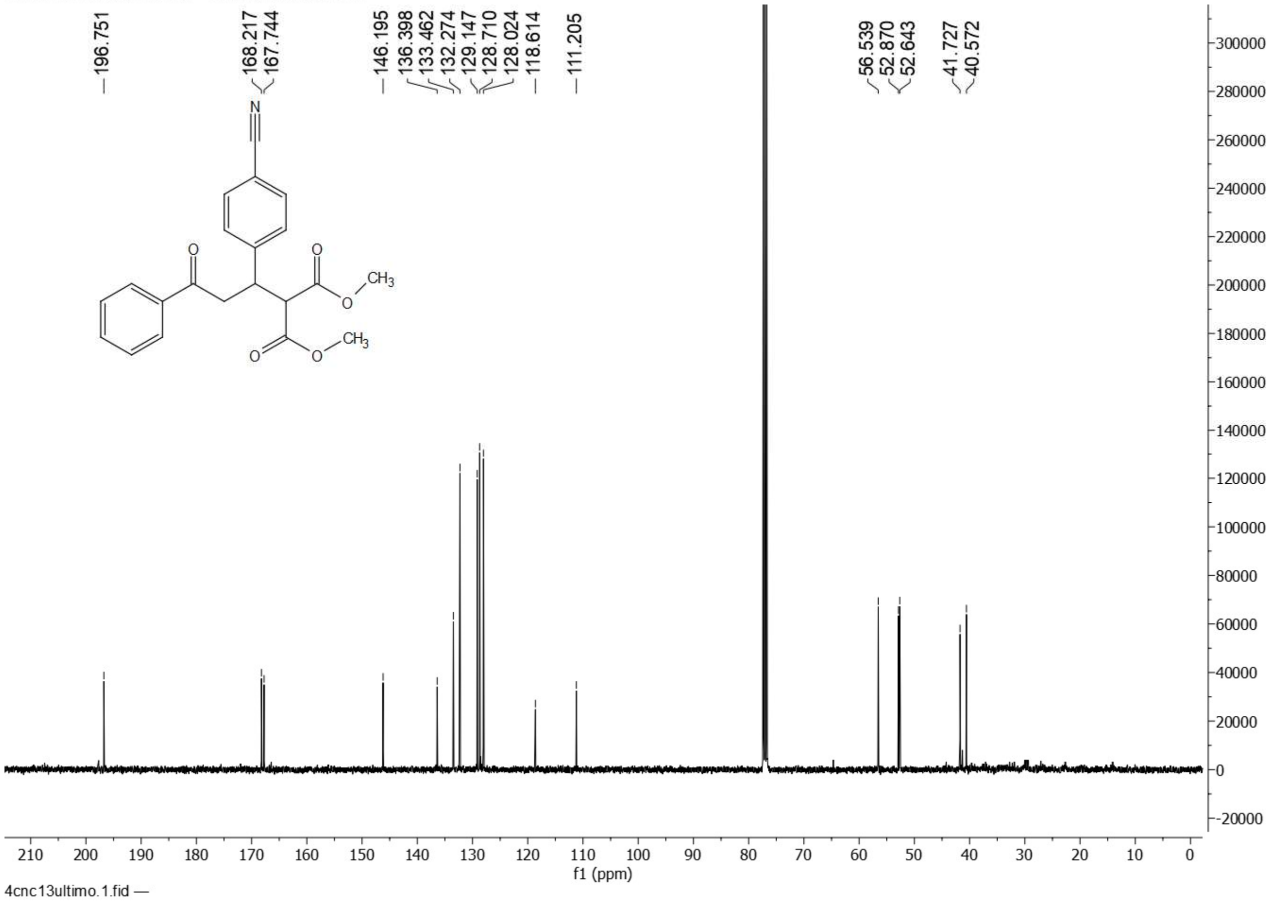

4cnc13ultimo.1.fid —

(*rac*)-Dimethyl 2-(1-(4-cyanophenyl)-3-oxo-3-phenylpropyl)malonate (6e)

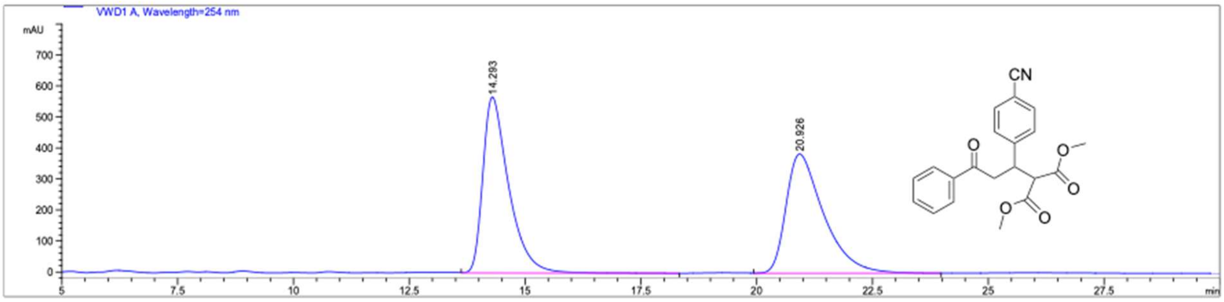

	Ret. Time (min)	Peak Area (mAU *min)	Peak Height (mAu)	Area %
1	14.293	222042.3	566.9	50.419
2	20.926	221676.3	383.9	49.581

(*R*)-Dimethyl 2-(1-(4-cyanophenyl)-3-oxo-3-phenylpropyl)malonate (6e)

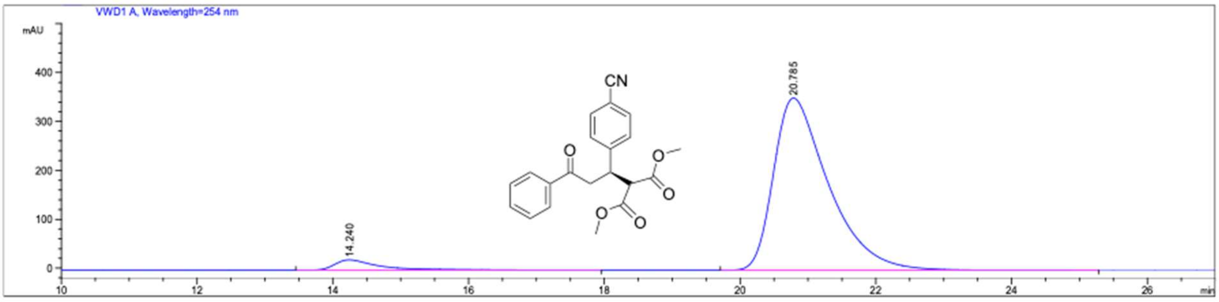

	Ret. Time (min)	Peak Area (mAU *min)	Peak Height (mAu)	Area %
1	14.240	1006.3	21.1	4.831
2	20.785	19825.8	351.9	95.169

Eluent: Hexane/ 2-propanol 3:2; 1 mL/min

(-)-Dimethyl 2-(1-(2-nitrophenyl)-3-oxo-3-phenylpropyl)malonate (6f)

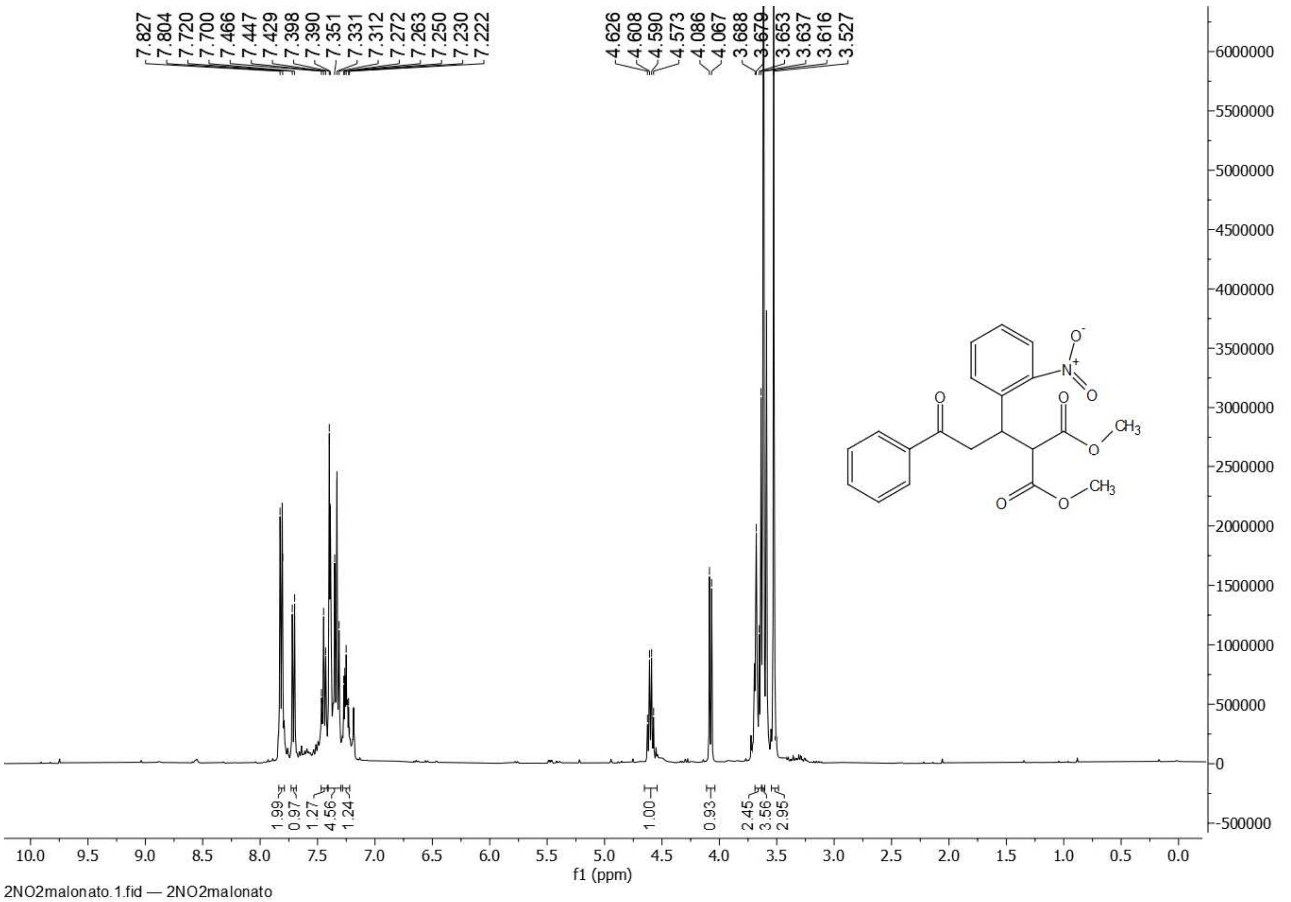

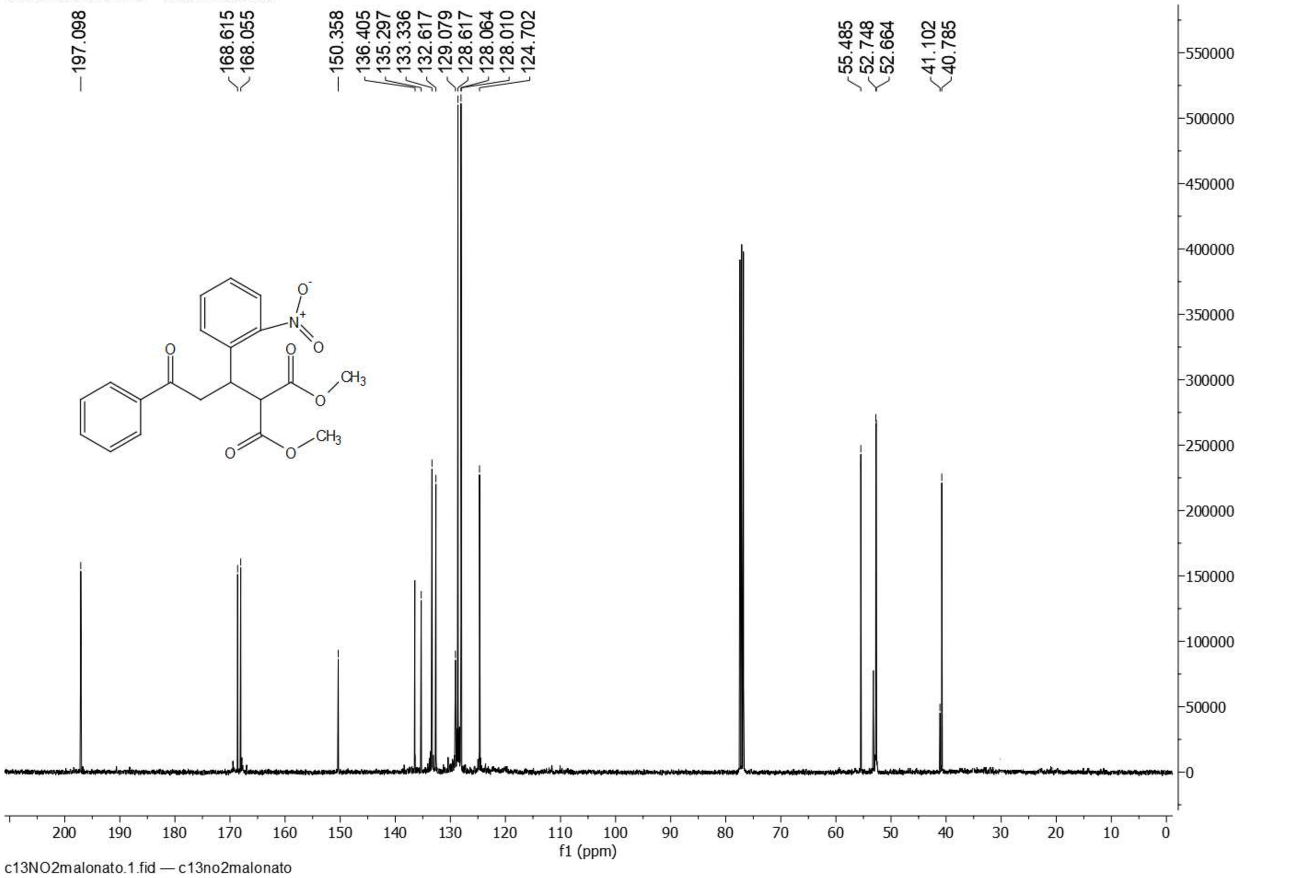

(*rac*)-Dimethyl 2-(1-(2-nitrophenyl)-3-oxo-3-phenylpropyl)malonate (6f)

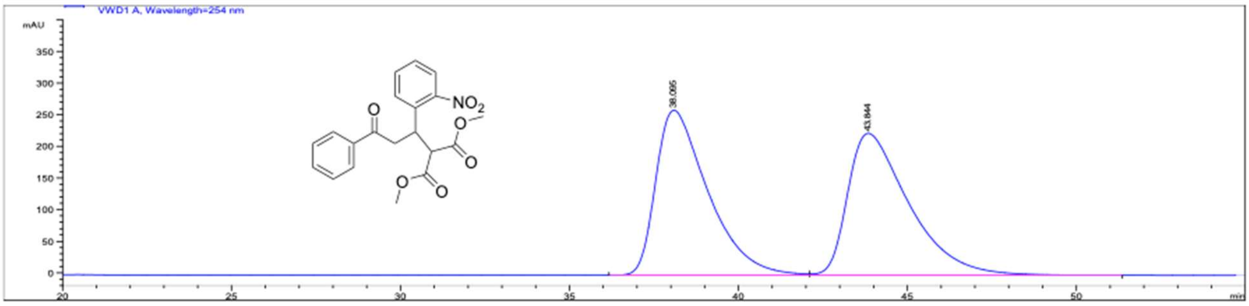

	Ret. Time (min)	Peak Area (mAU *min)	Peak Height (mAu)	Area %
1	38.095	28029.5	260.9	49.721
2	43.844	28344.6	224.4	50.279

(-)-Dimethyl 2-(1-(2-nitrophenyl)-3-oxo-3-phenylpropyl)malonate (6f)

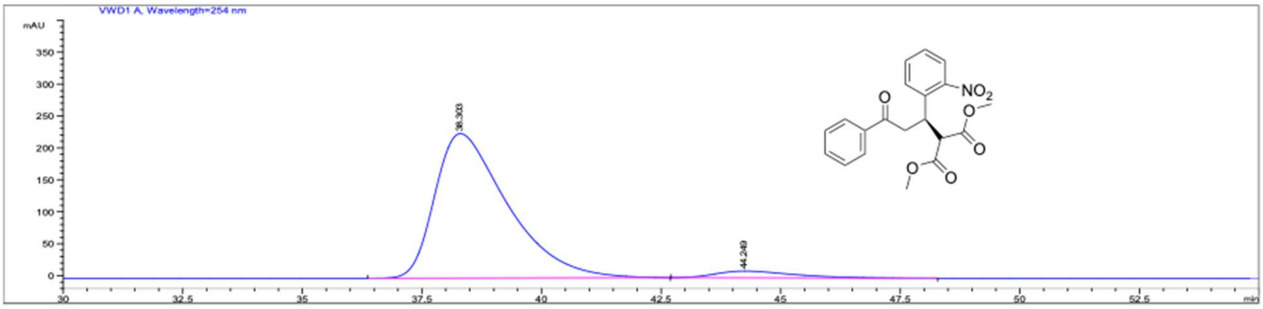

	Ret. Time (min)	Peak Area (mAU *min)	Peak Height (mAu)	Area %
1	38.303	23925.4	226.2	95.266
2	44.249	1188.8.6	10.5	4.734

Eluent: Hexane/ 2-propanol 3:2; 0.8 mL/min

(-)-Dimethyl 2-(1-(3-nitrophenyl)-3-oxo-3-phenylpropyl)malonate (6g)

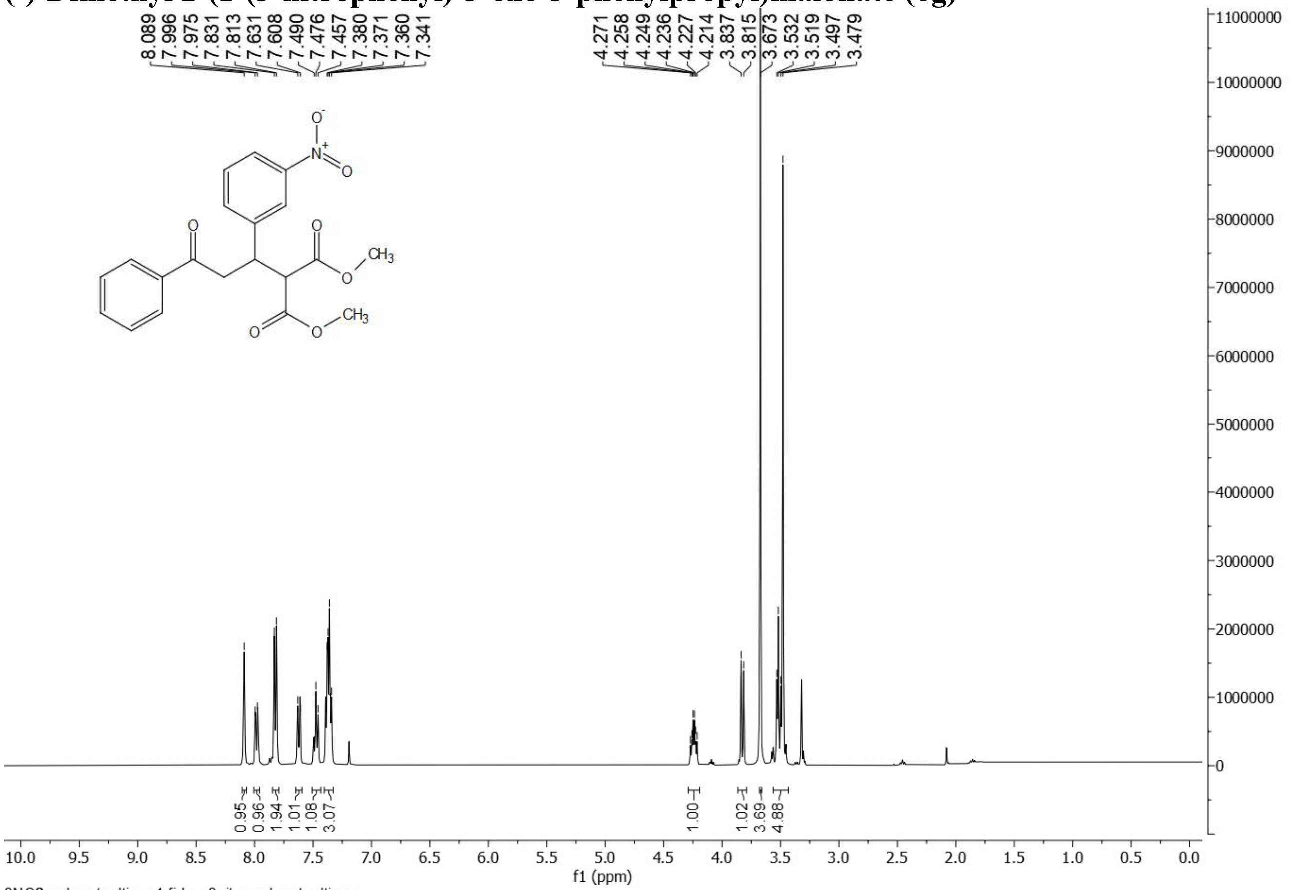

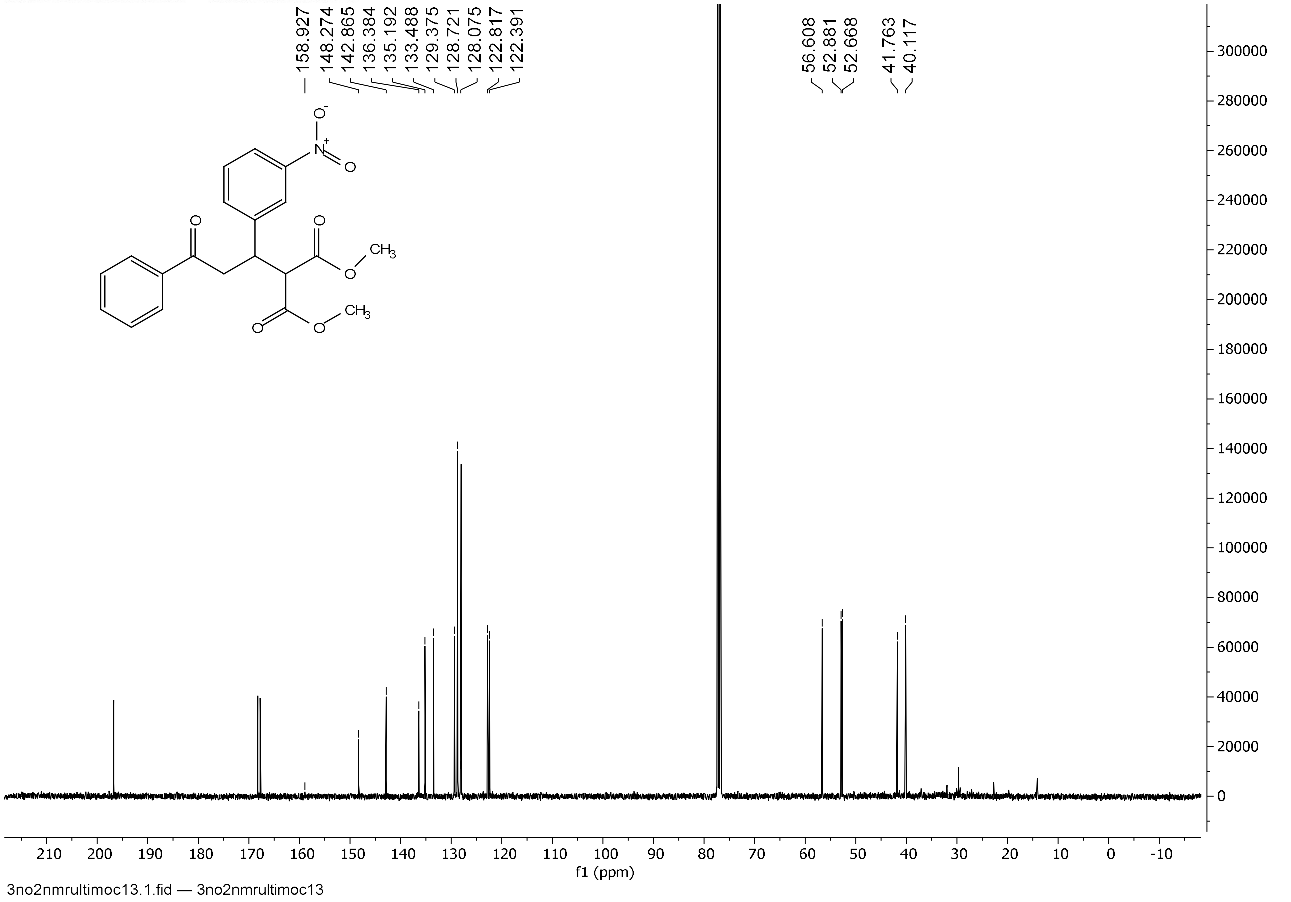

(*rac*)-Dimethyl 2-(1-(3-nitrophenyl)-3-oxo-3-phenylpropyl)malonate (6g)

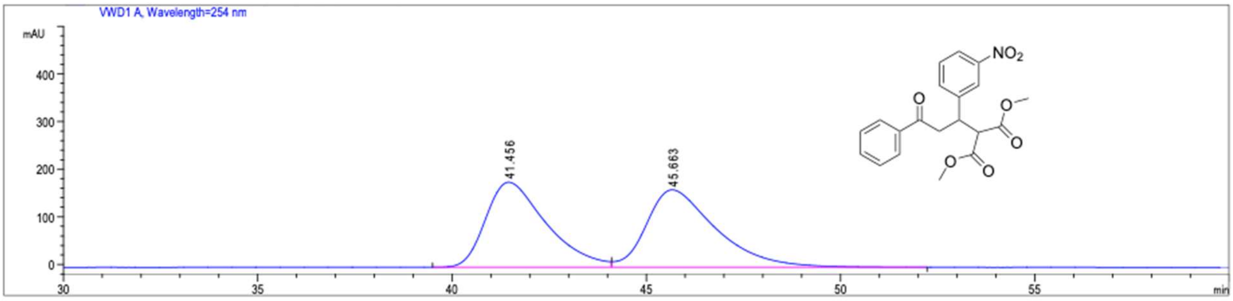

	Ret. Time (min)	Peak Area (mAU *min)	Peak Height (mAu)	Area %
1	41.456	19378.4	178.5	48.979
2	45.663	20186.0	162.5	51.021

(-)-Dimethyl 2-(1-(3-nitrophenyl)-3-oxo-3-phenylpropyl)malonate (6g)

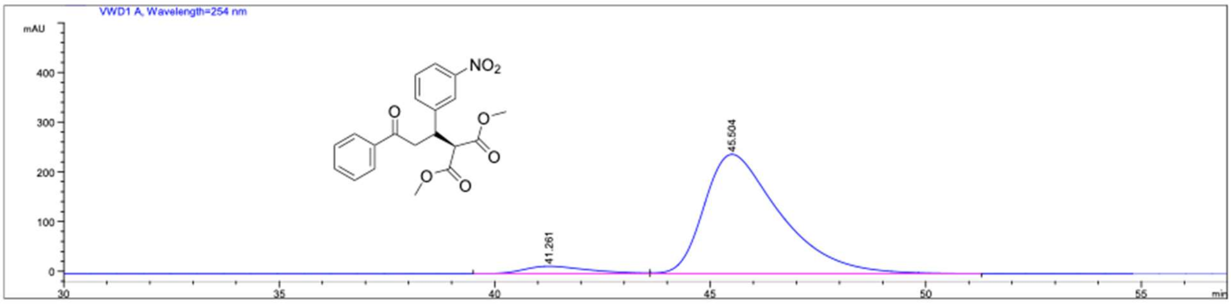

	Ret. Time (min)	Peak Area (mAU *min)	Peak Height (mAu)	Area %
1	41.261	1552.7	15.0	5.012
2	45.663	29426.1	240.1.5	94.988

Eluent: Hexane/ 2-propanol 3:2; 0.8 mL/min

(*R*)-Dimethyl 2-(3-oxo-3-phenyl-1-(thiophen-2-yl)propyl)malonate (6h)

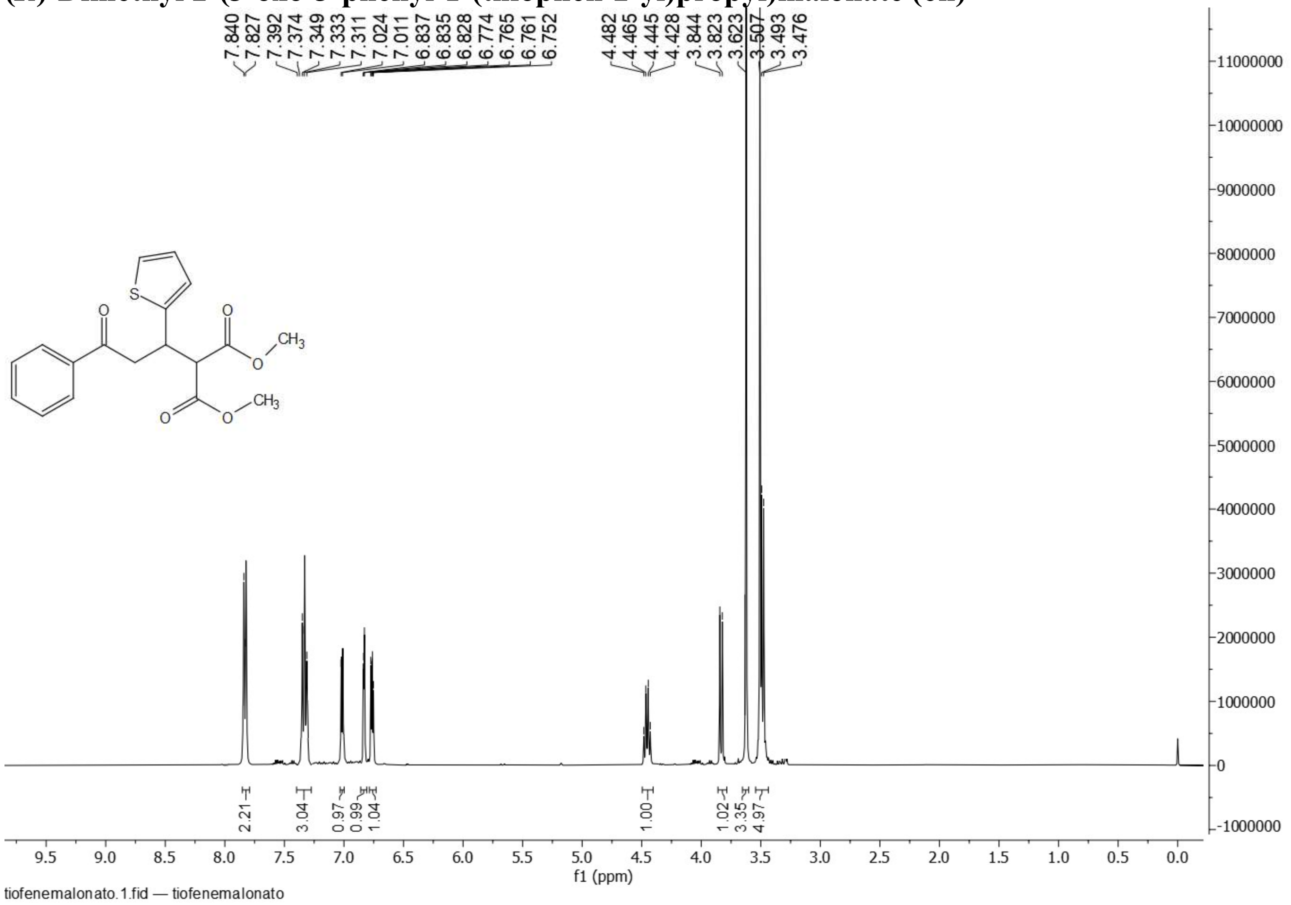

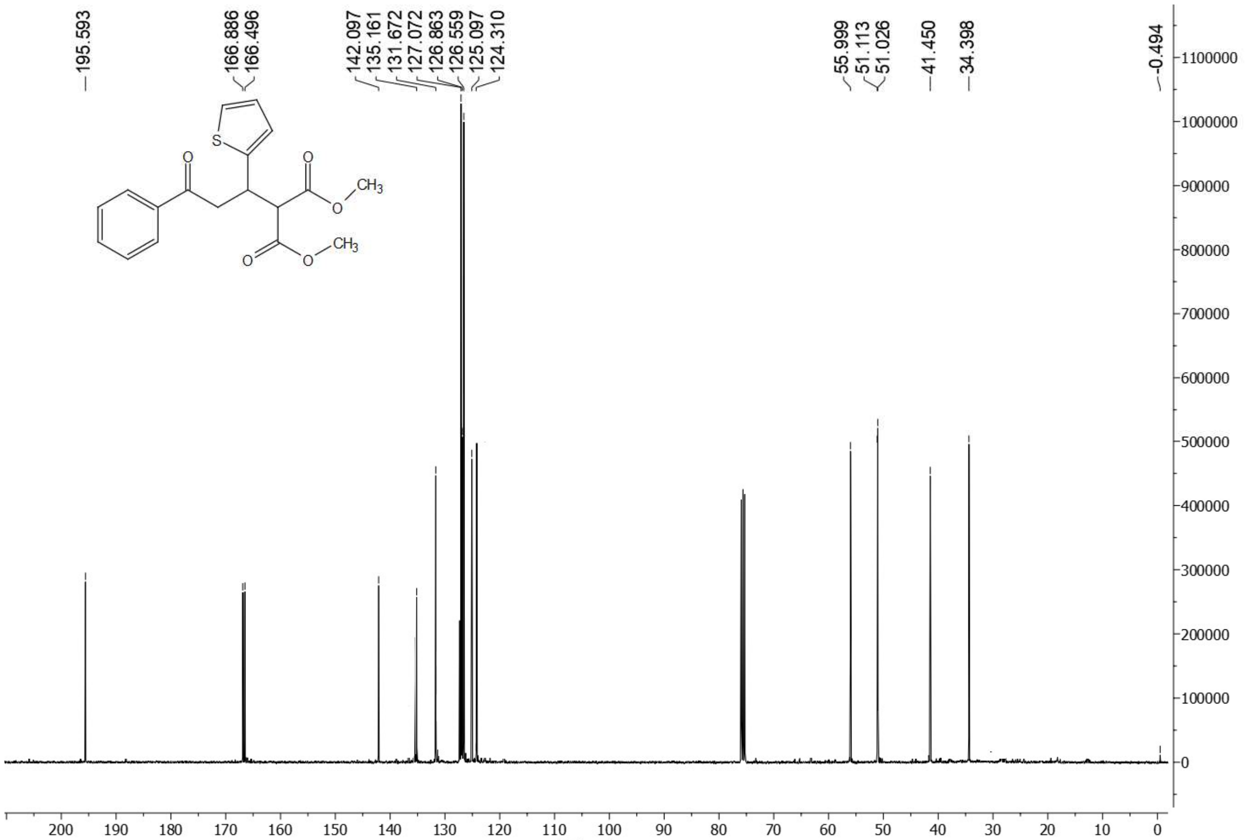

(*rac*)-Dimethyl 2-(3-oxo-3-phenyl-1-(thiophen-2-yl)propyl)malonate (6h)

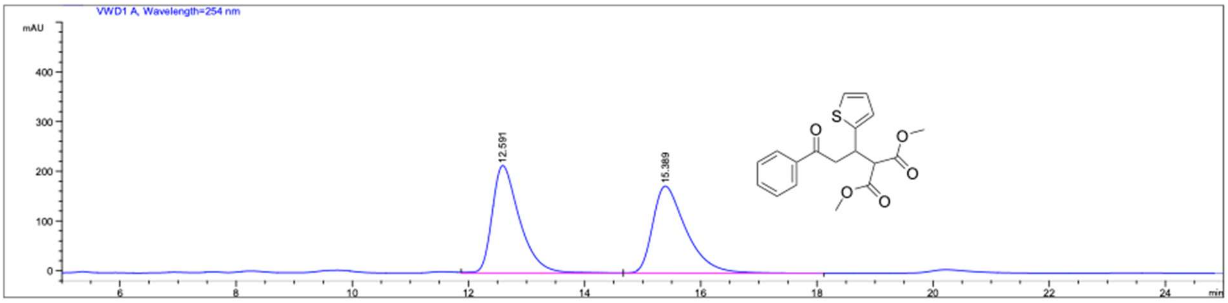

	Ret. Time (min)	Peak Area (mAU *min)	Peak Height (mAu)	Area %
1	12.591	7096.1	216.1	50.415
2	15.389	6979.3	174.8	49.585

(*R*)-Dimethyl 2-(3-oxo-3-phenyl-1-(thiophen-2-yl)propyl)malonate (6h)

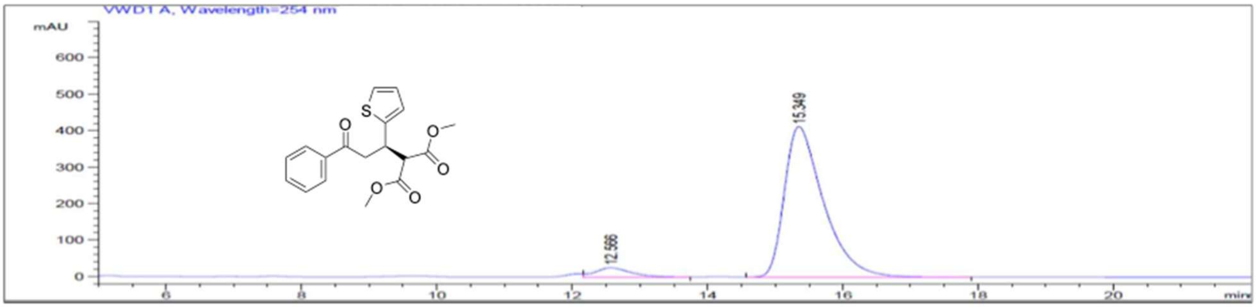

	Ret. Time (min)	Peak Area (mAU *min)	Peak Height (mAu)	Area %
1	12.566	911.4	25.2	5.205
2	15.389	16598.2	413.1	94.795

Eluent: Hexane/ 2-propanol 3:2; 1.0 mL/min

(*R*)-Dimethyl 2-(1-(furan-2-yl)-3-oxo-3-phenylpropyl)malonate (6i)

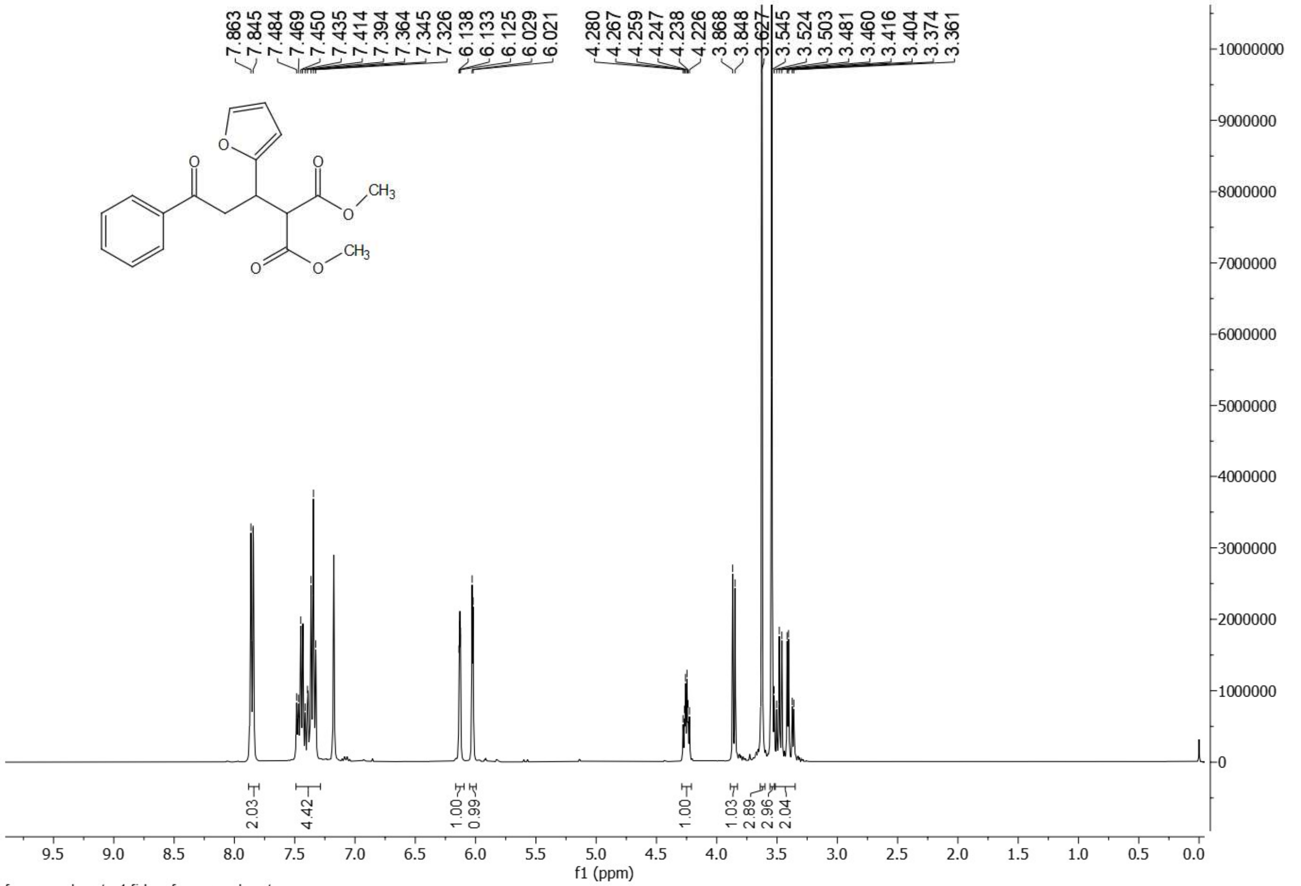

furanomalonato.1.fid — furanomalonato

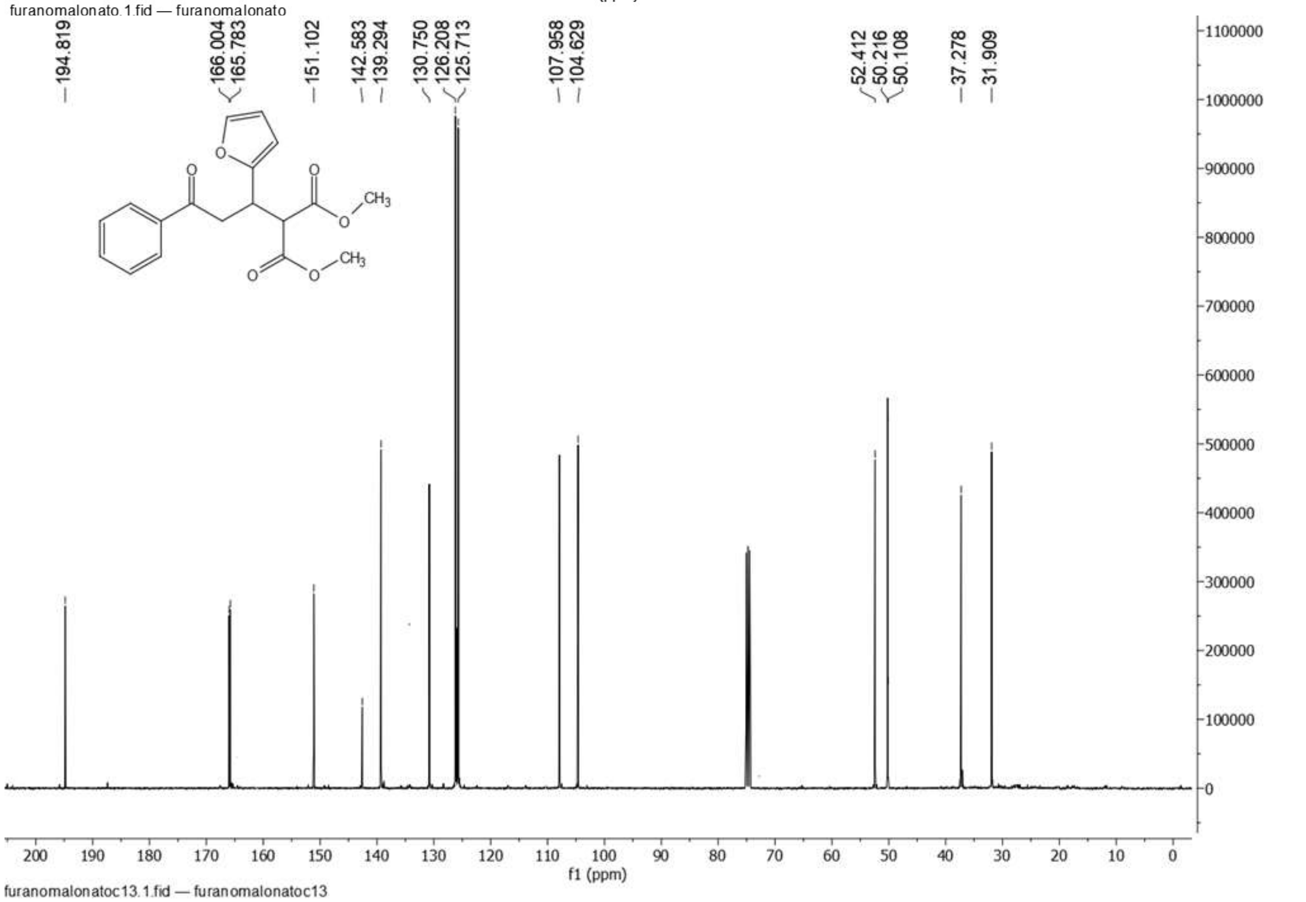

furanomalonatoc13.1.fid — furanomalonatoc13

(*rac*)-Dimethyl 2-(1-(furan-2-yl)-3-oxo-3-phenylpropyl)malonate (6i)

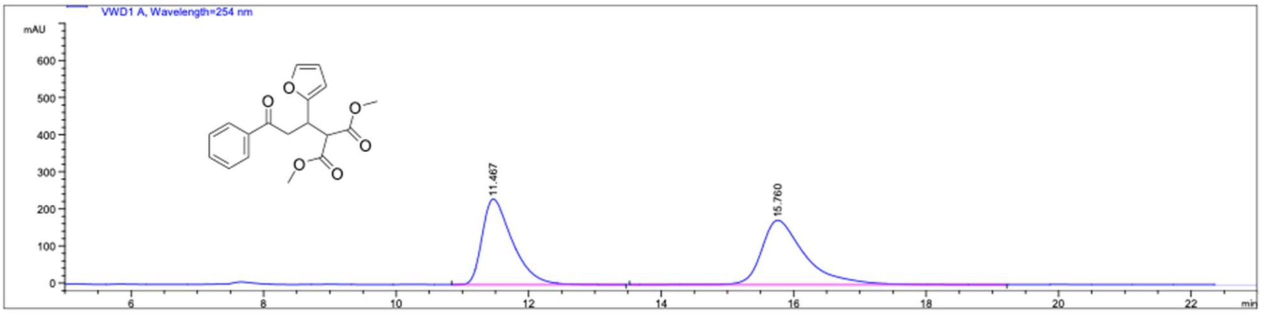

	Ret. Time (min)	Peak Area (mAU *min)	Peak Height (mAu)	Area %
1	11.467	7302.9	230.3	48.147
2	15.389	7865.1	173.3	51.583

(*R*)-Dimethyl 2-(1-(furan-2-yl)-3-oxo-3-phenylpropyl)malonate (6i)

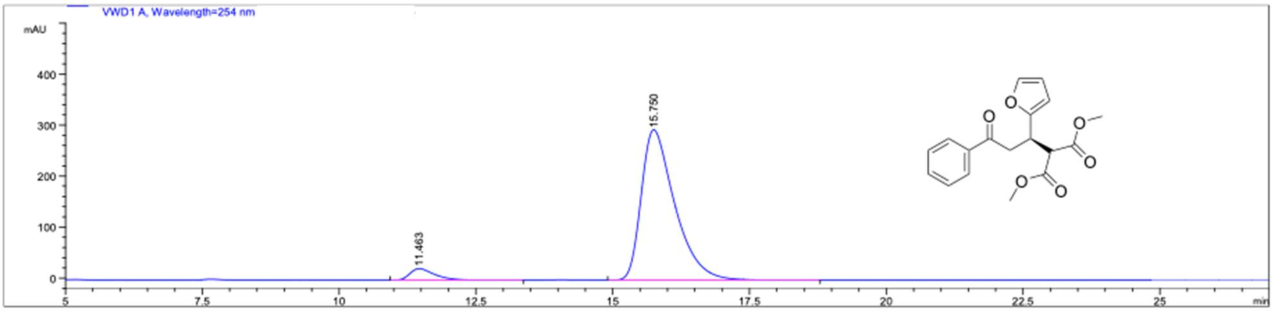

	Ret. Time (min)	Peak Area (mAU *min)	Peak Height (mAu)	Area %
1	11.463	708.0	22.5	5.338
2	15.750	12556.8	294.8	94.662

Eluent: Hexane/ 2-propanol 3:2; 1.0 mL/min

(*R*)-Dimethyl 2-(3-oxo-1-phenylbutyl)malonate (6j)

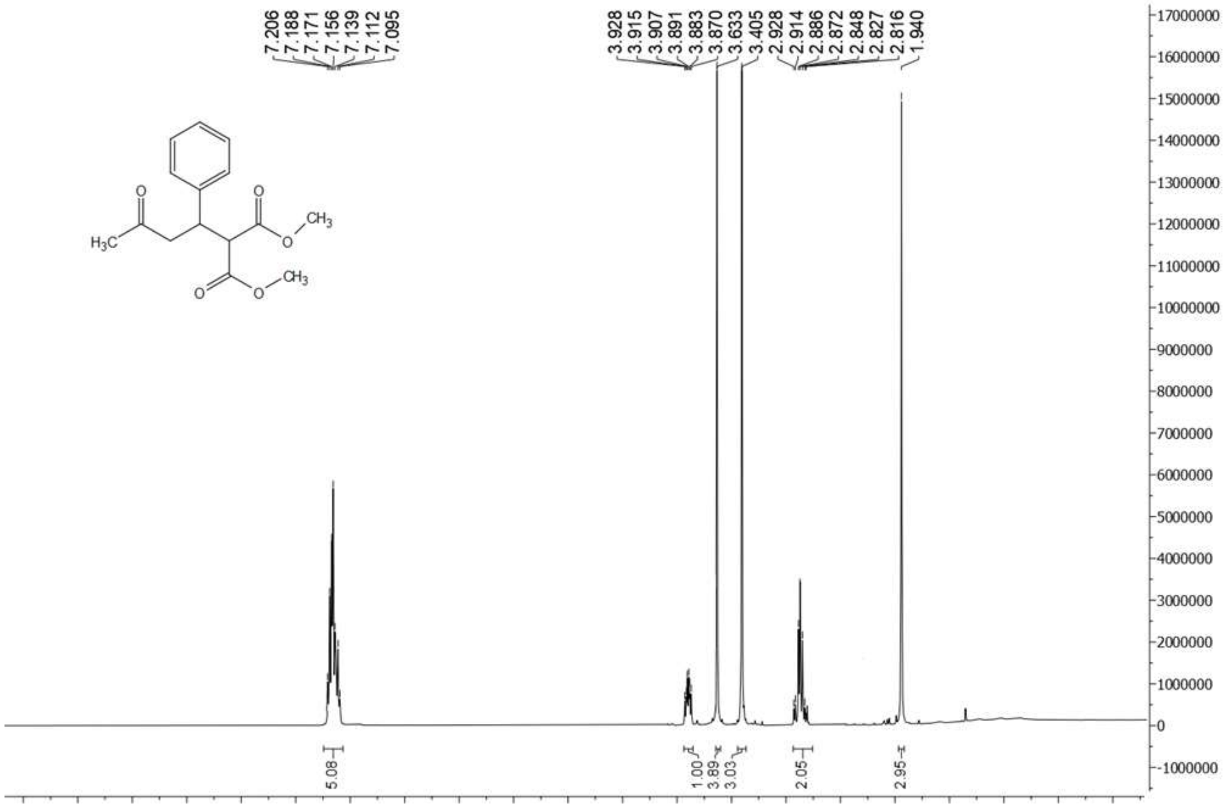

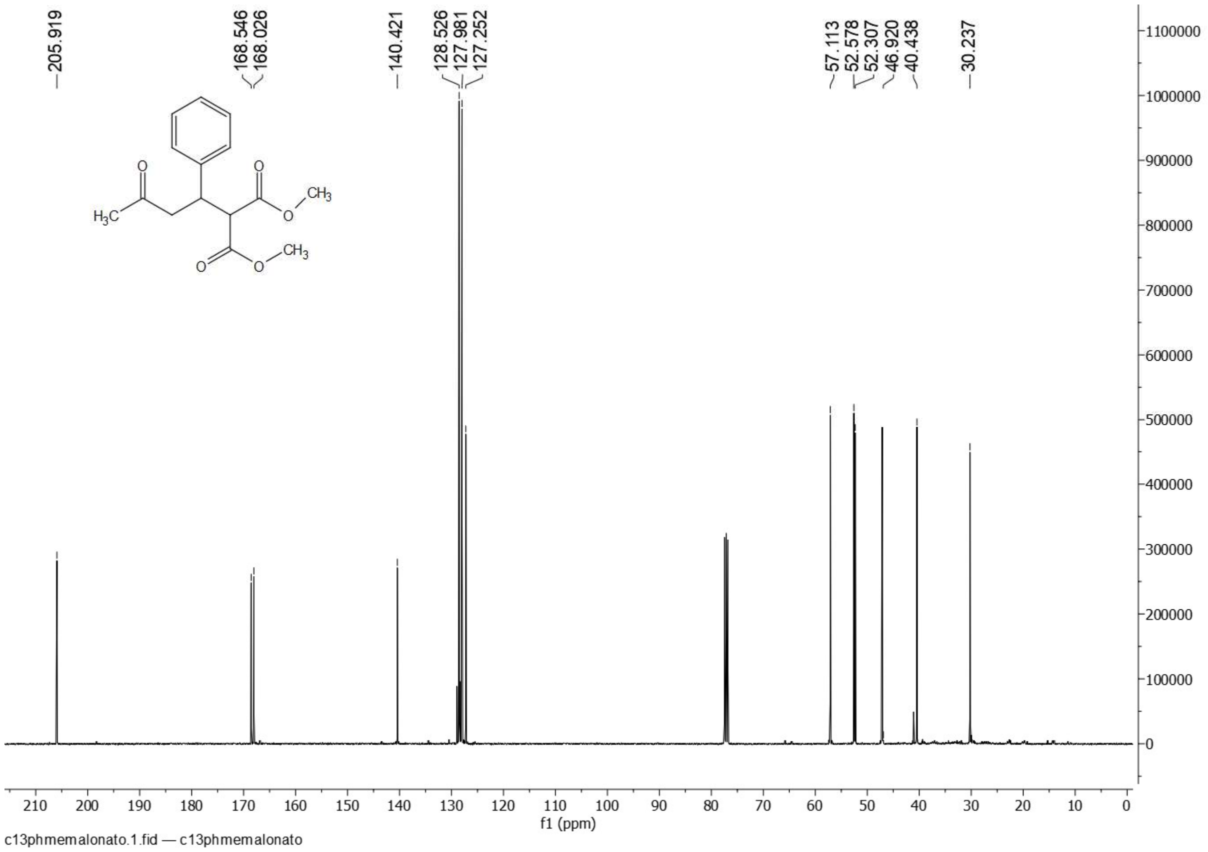

(*rac*)-Dimethyl 2-(3-oxo-1-phenylbutyl)malonate (6j)

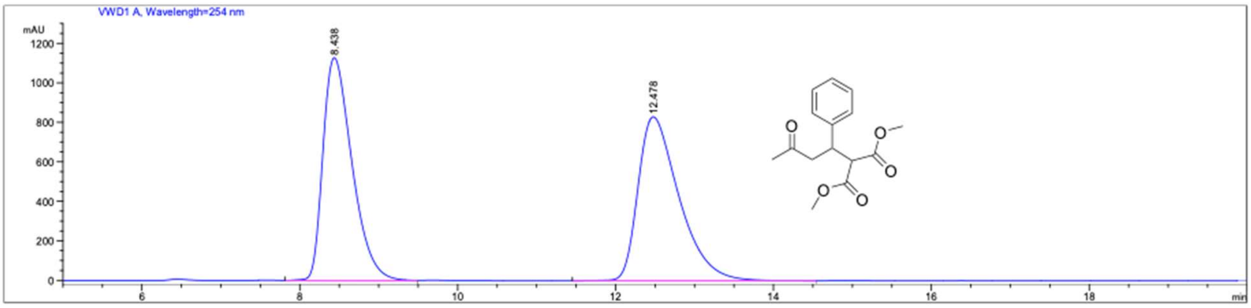

	Ret. Time (min)	Peak Area (mAU *min)	Peak Height (mAu)	Area %
1	8.438	28966.0	1129.3	49.129
2	12.478	29992.8	830.2	50.871

(*R*)-Dimethyl 2-(3-oxo-1-phenylbutyl)malonate (6j)

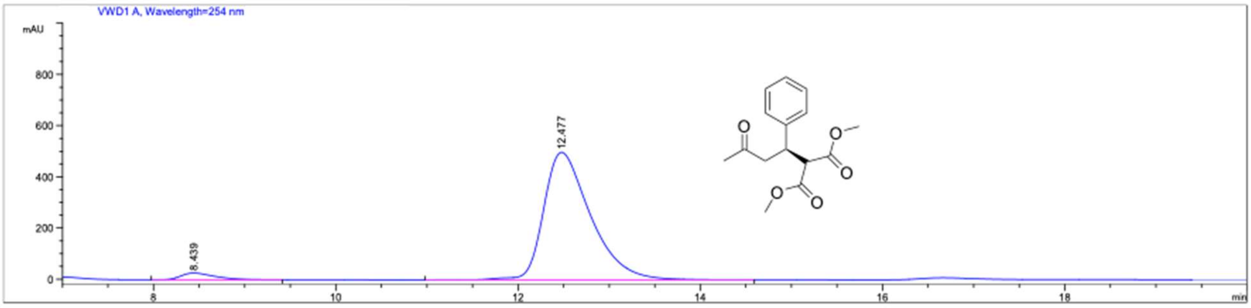

	Ret. Time (min)	Peak Area (mAU *min)	Peak Height (mAu)	Area %
1	8.439	690.9.0	26.5	3.741
2	12.477	17780.1	497.8	96.259

Eluent: Hexane/ 2-propanol 3:2; 1.0 mL/min

(-)-Dimethyl 2-(3-oxo-1-(*o*-tolyl)butyl)malonate (6k)

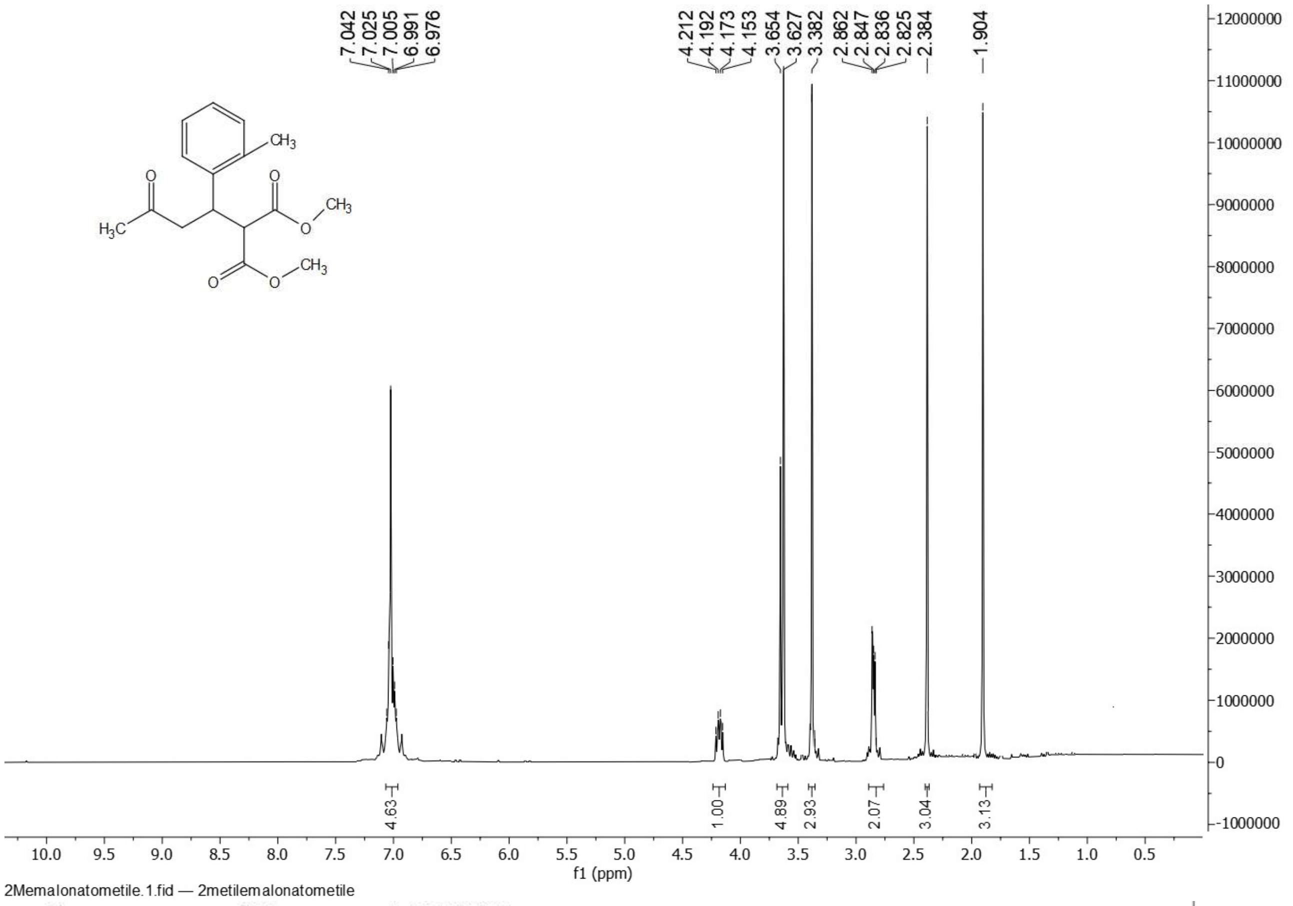

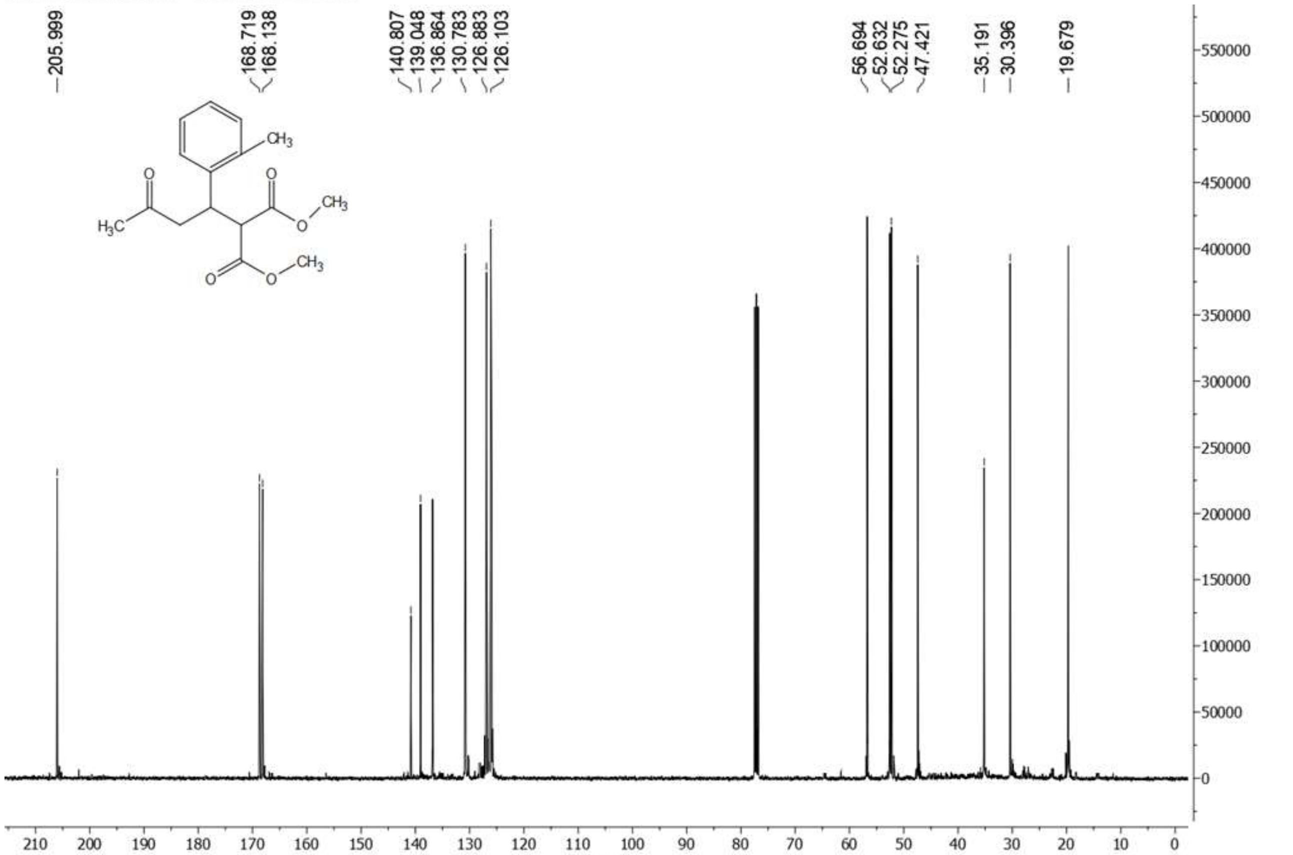

(*rac*)-Dimethyl 2-(3-oxo-1-(*o*-tolyl)butyl)malonate (6k)

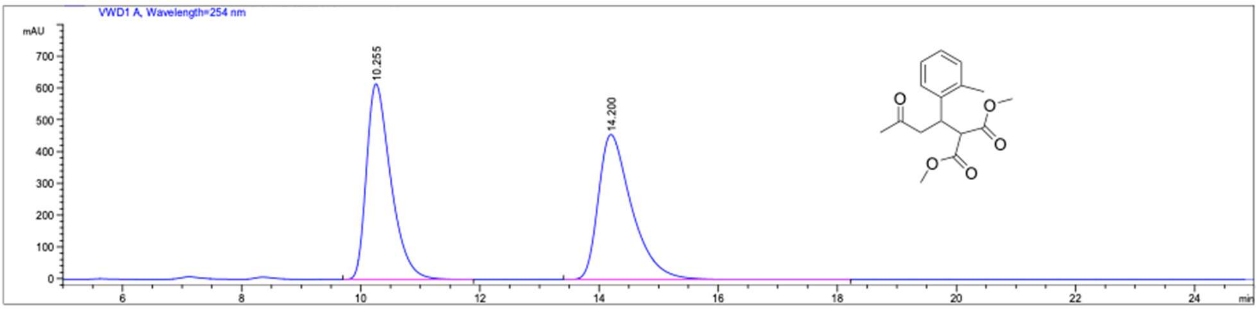

	Ret. Time (min)	Peak Area (mAU *min)	Peak Height (mAu)	Area %
1	10.255	17482.3	615.7	49.573
2	14.200	17783.3	456.2	50.427

(-)-Dimethyl 2-(3-oxo-1-(*o*-tolyl)butyl)malonate (6k)

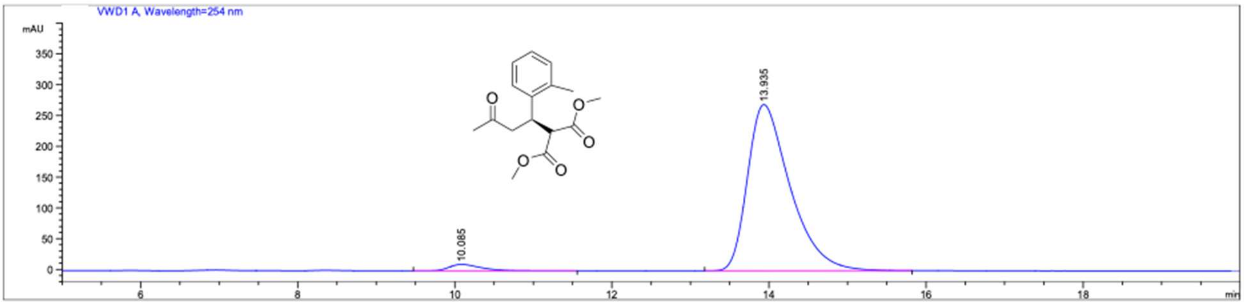

	Ret. Time (min)	Peak Area (mAU *min)	Peak Height (mAu)	Area %
1	10.085	292.3	10.5	2.796
2	13.935	10161.6	296.8	97.204

Eluent: Hexane/ 2-propanol 3:2; 1.0 mL/min

(-)-Dimethyl 2-(1-(3-methoxyphenyl)-3-oxobutyl)malonate (6l)

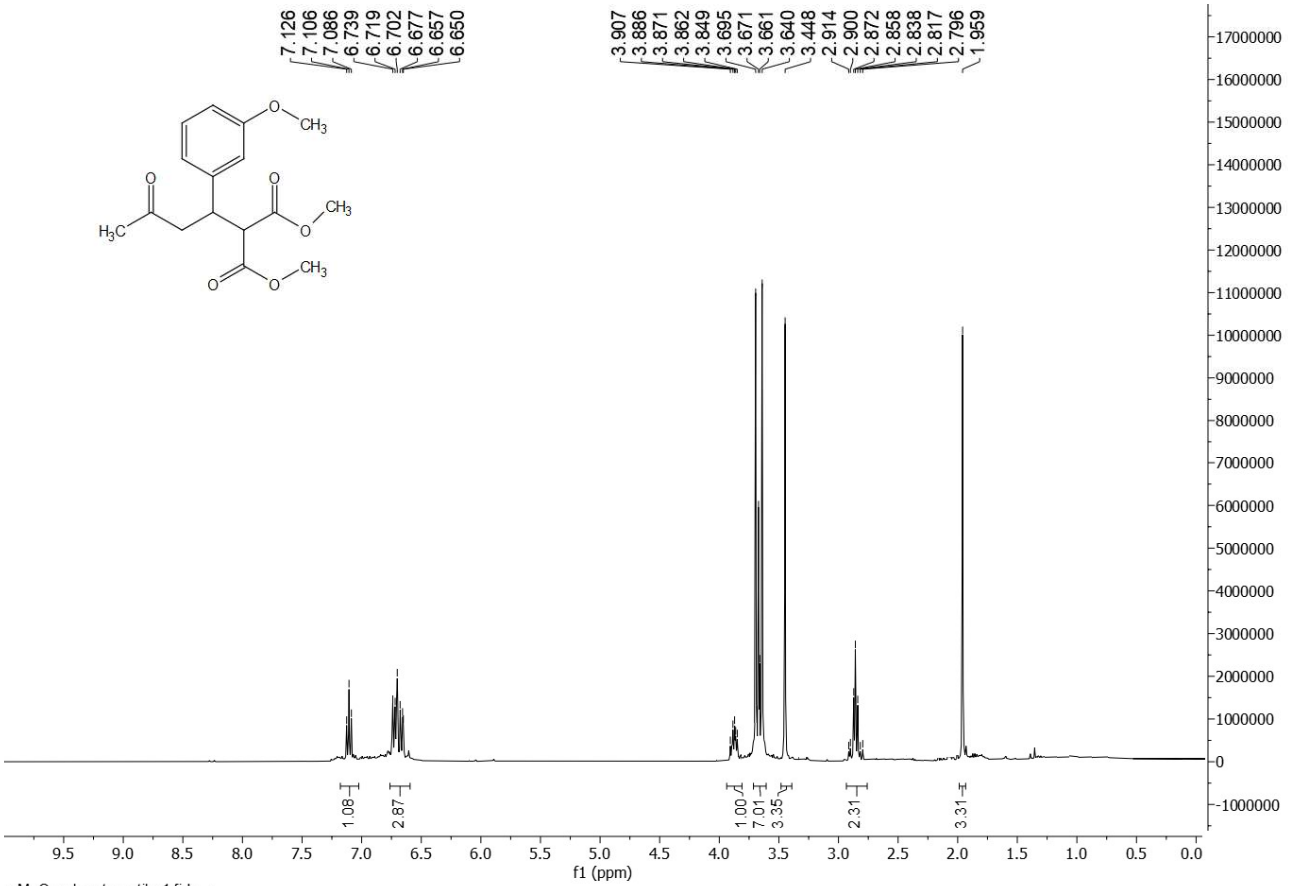

mMeOmalonatometile.1.fid —

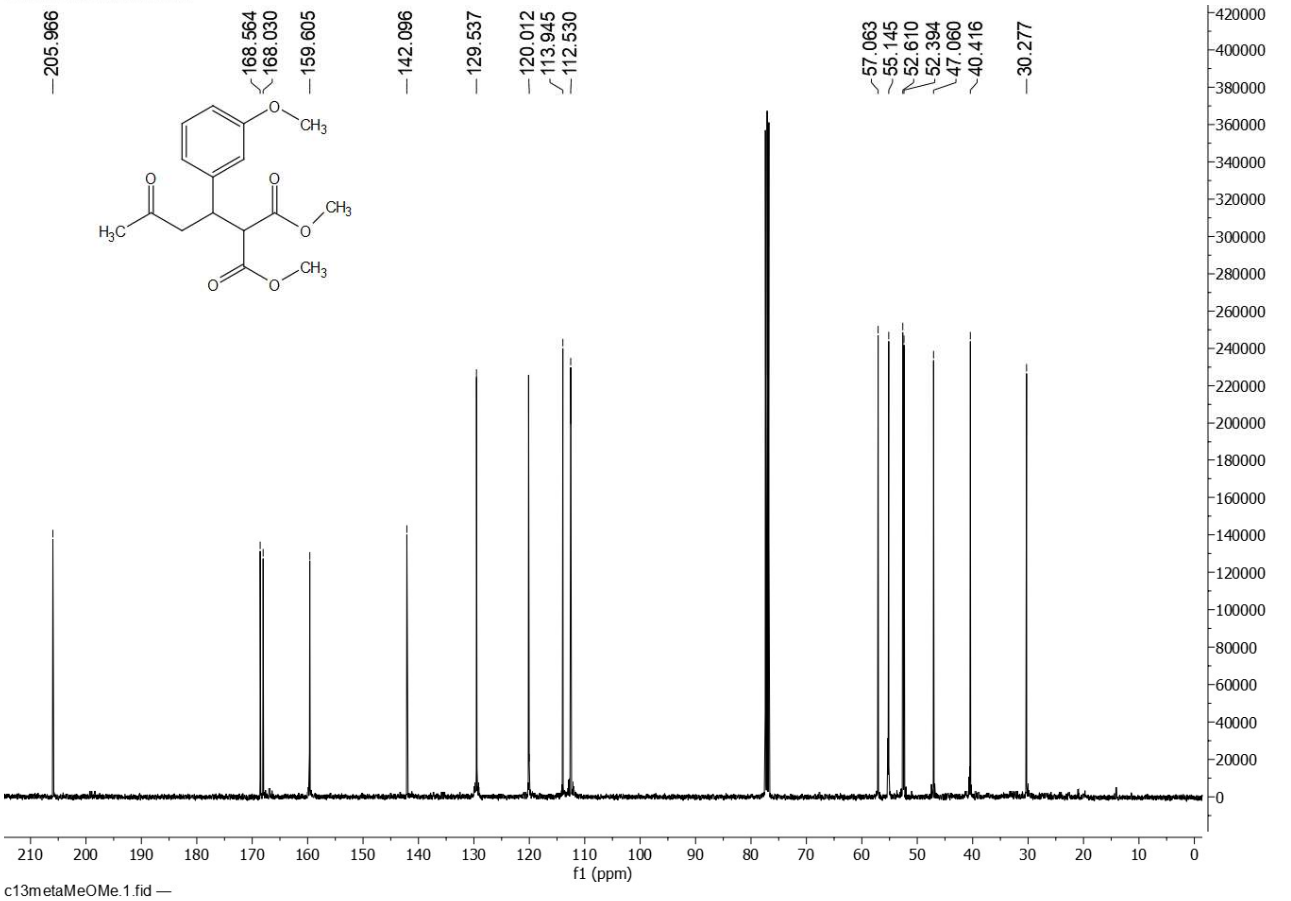

c13metaMeOMe.1.fid —

(*rac*)-Dimethyl 2-(1-(3-methoxyphenyl)-3-oxobutyl)malonate (6l)

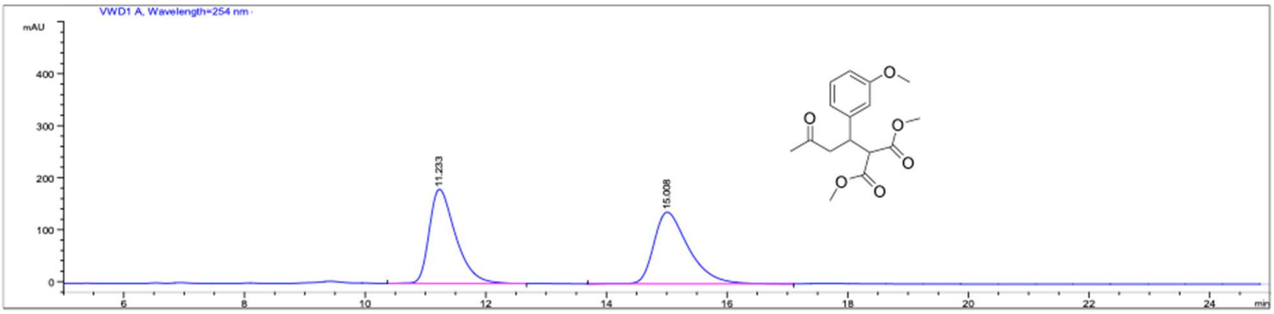

	Ret. Time (min)	Peak Area (mAU *min)	Peak Height (mAu)	Area %
1	11.233	5534.3	181.4	49.994
2	15.008	5535.6	137.9	50.006

(-)-Dimethyl 2-(1-(3-methoxyphenyl)-3-oxobutyl)malonate (6l)

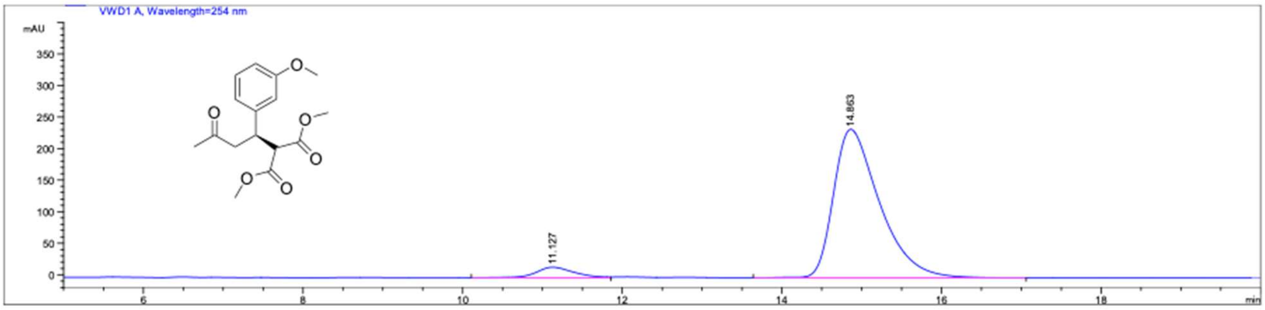

	Ret. Time (min)	Peak Area (mAU *min)	Peak Height (mAu)	Area %
1	11.127	555.4	16.7	5.558
2	14.863	9437.5	235.3	94.442

Eluent: Hexane/ 2-propanol 3:2; 1.0 mL/min

(*R*)-Dimethyl 2-(4-oxo-4-phenylbutan-2-yl)malonate (6m)

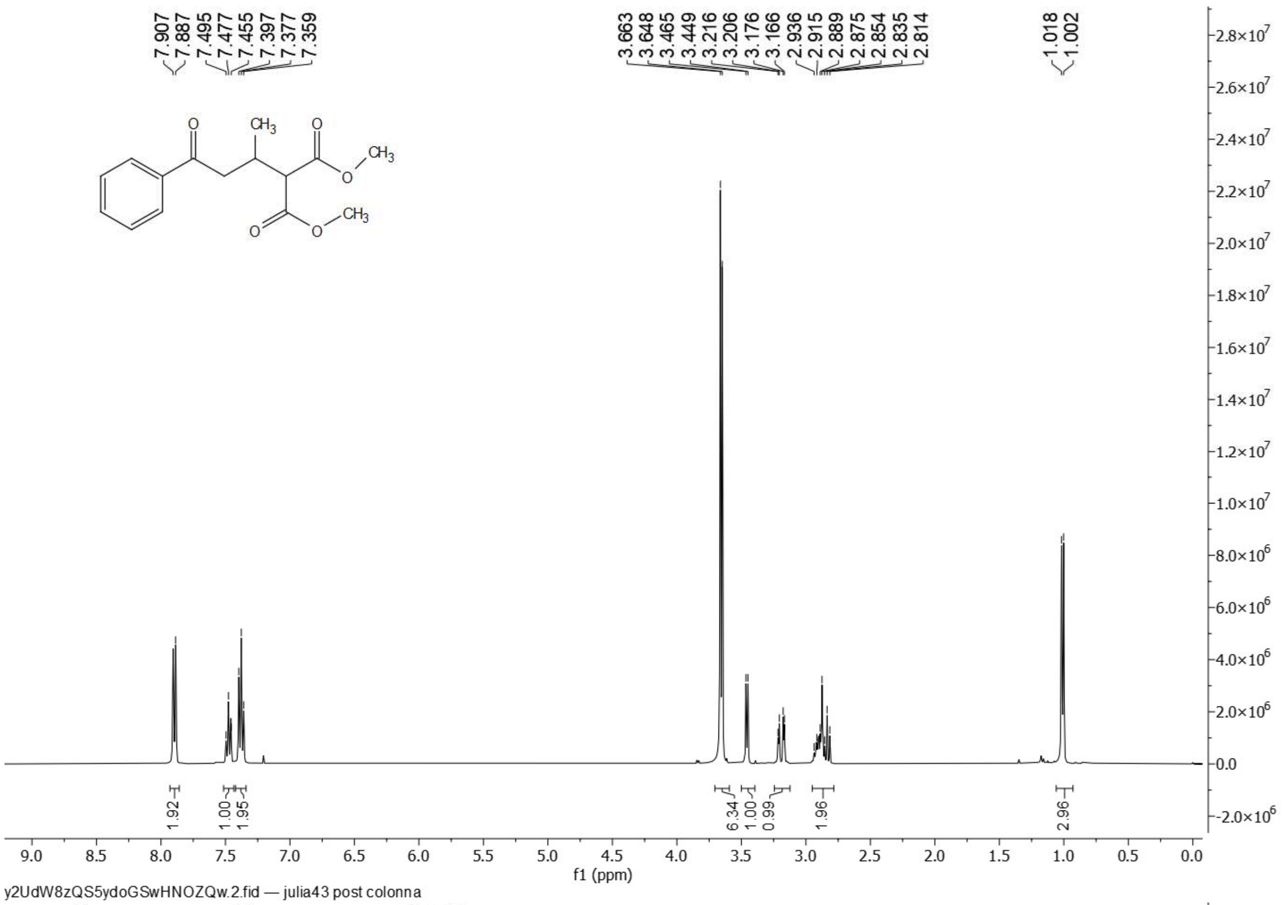

y2UdW8zQS5ydoGSwHNOZQw.2.fid — julia43 post colonna

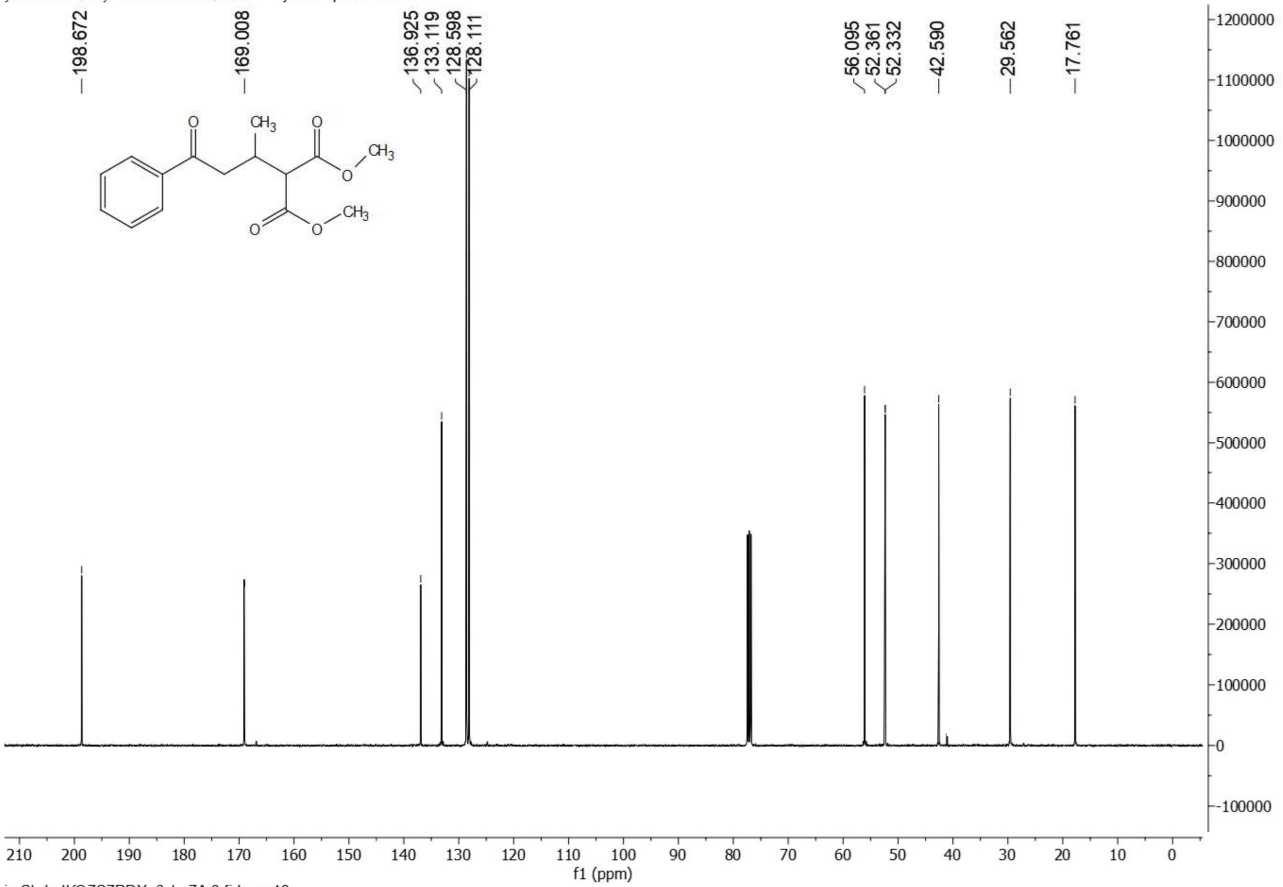

imShdpdKQ7S7PDXa6shc7A.3.fid — c13

(*rac*)-Dimethyl 2-(4-oxo-4-phenylbutan-2-yl)malonate (6m)

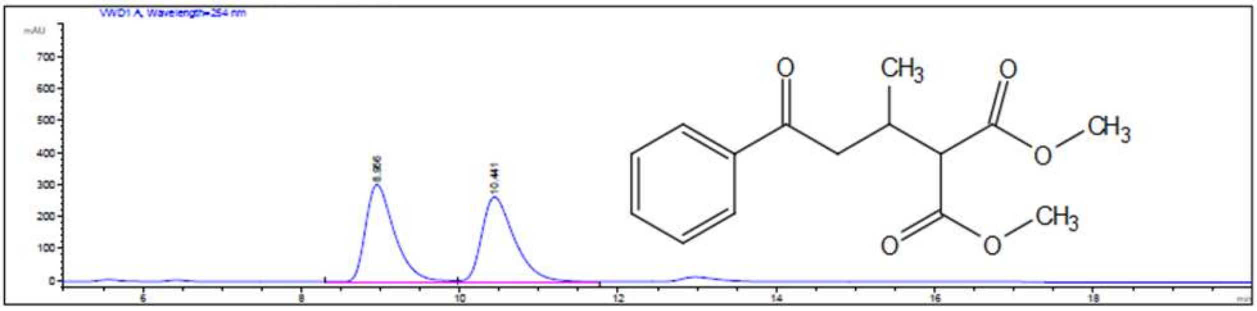

	Ret. Time (min)	Peak Area (mAU *min)	Peak Height (mAu)	Area %
1	8.956	7661.1	303.2	50.420
2	10.441	7533.6	264.7	49.580

(*R*)-Dimethyl 2-(4-oxo-4-phenylbutan-2-yl)malonate (6m)

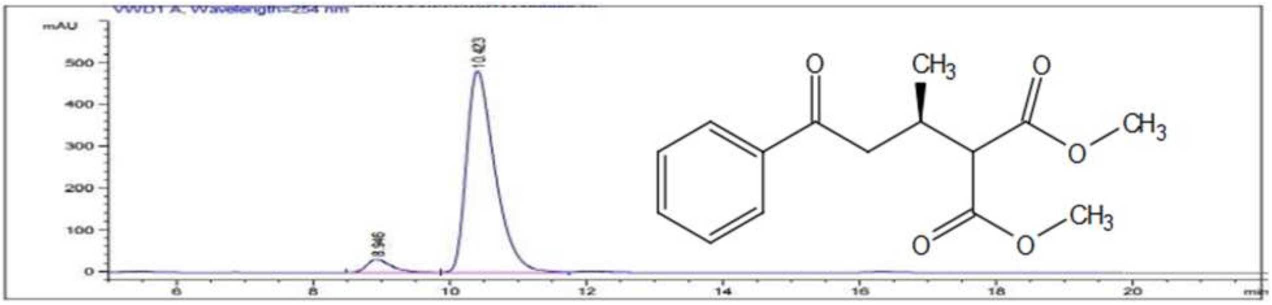

	Ret. Time (min)	Peak Area (mAU *min)	Peak Height (mAu)	Area %
1	8.946	77.28	31.1	5.258
2	10.423	1392.41	482.7	94.742

Eluent: Hexane/ 2-propanol 3:2; 1.0 mL/min

(-)-Dimethyl 2-(3-oxo-1-phenyl-3-(4-(trifluoromethyl)phenyl)propyl)malonate (6n)

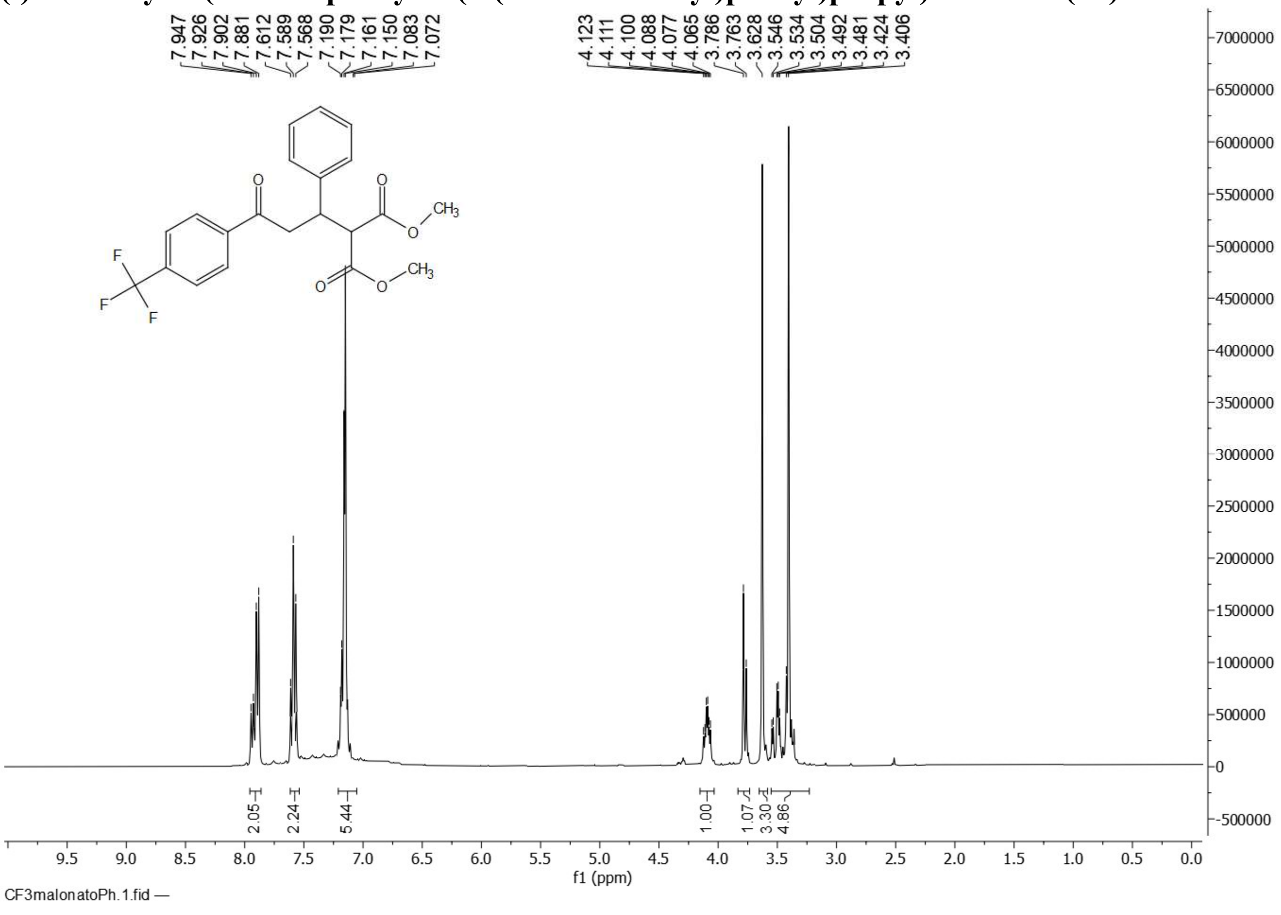

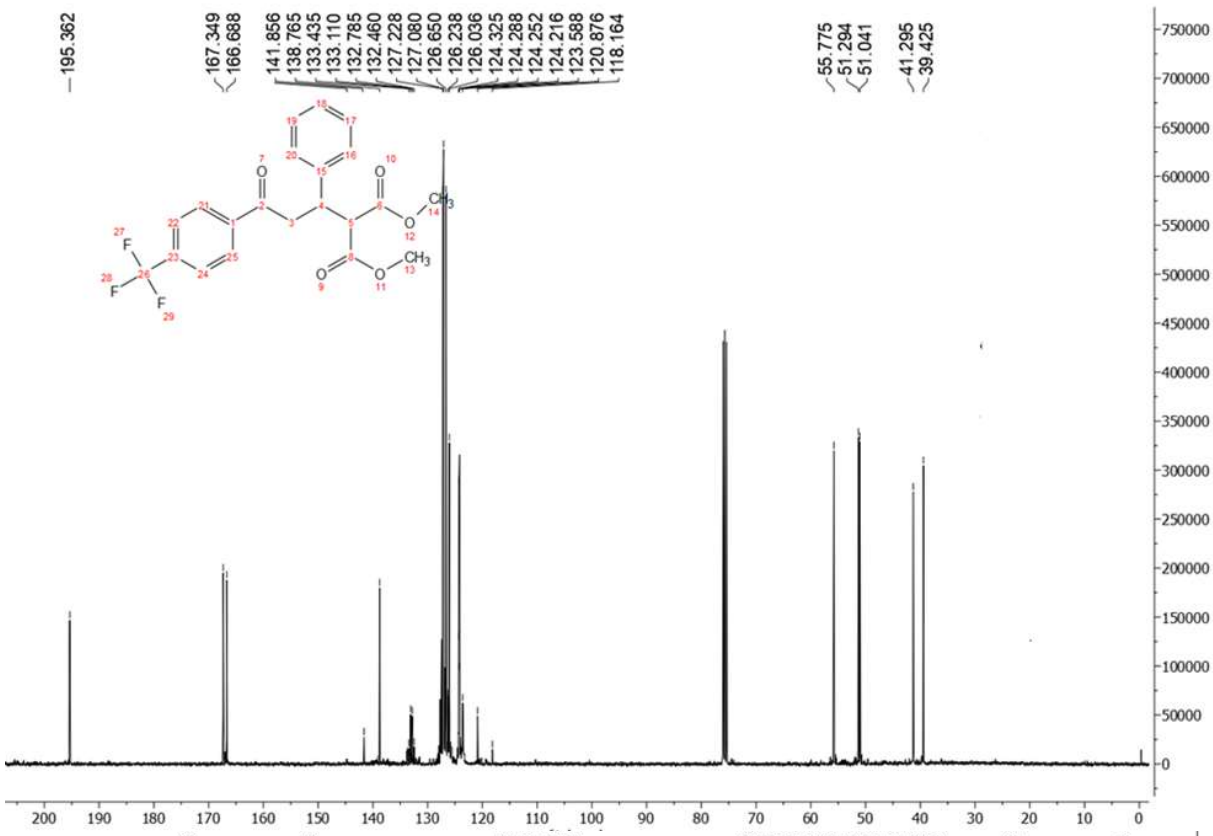
195.362
167.349
166.688
141.856
138.765
133.435
133.110
132.785
132.460
127.228
127.080
126.650
126.238
126.036
124.325
124.288
124.252
124.216
123.588
120.876
118.164
55.775
51.294
51.041
41.295
39.425
F
F
F
O
O
O
O
O
O
CH3
CH3

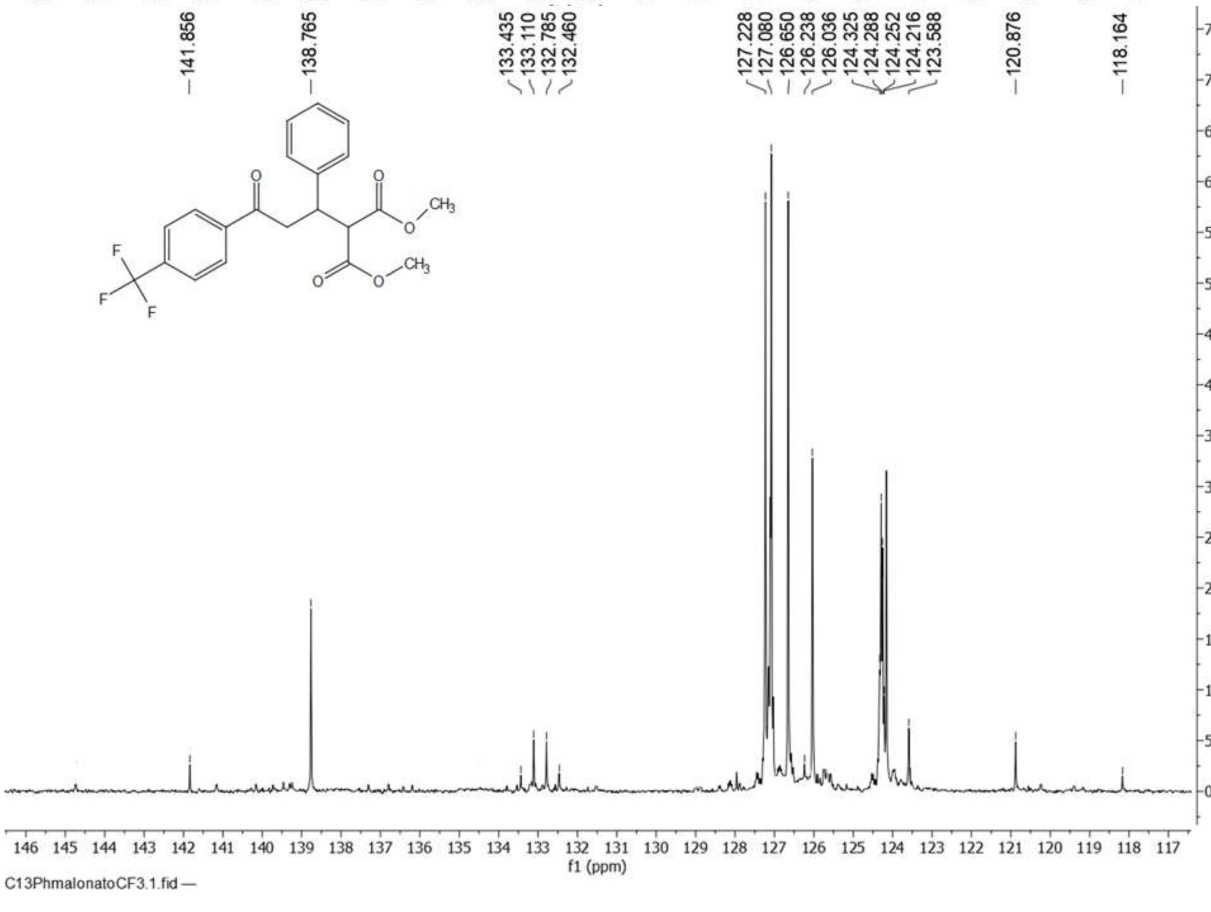
141.856
138.765
133.435
133.110
132.785
132.460
127.228
127.080
126.650
126.238
126.036
124.325
124.288
124.252
124.216
123.588
120.876
118.164
F
F
F
O
O
O
O
O
O
CH3
CH3
f1 (ppm)
C13PhmalonatoCF3.1.fid —

(*rac*)-Dimethyl 2-(3-oxo-1-phenyl-3-(4-(trifluoromethyl)phenyl)propyl)malonate (6n)

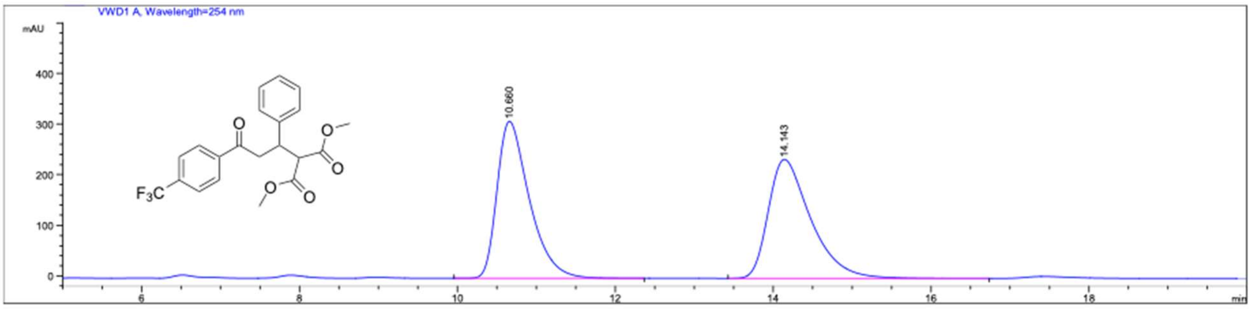

	Ret. Time (min)	Peak Area (mAU *min)	Peak Height (mAu)	Area %
1	10.660	8798.8	309.7	50.048
2	14.413	8781.8	234.8	49.952

(-)-Dimethyl 2-(3-oxo-1-phenyl-3-(4-(trifluoromethyl)phenyl)propyl)malonate (6n)

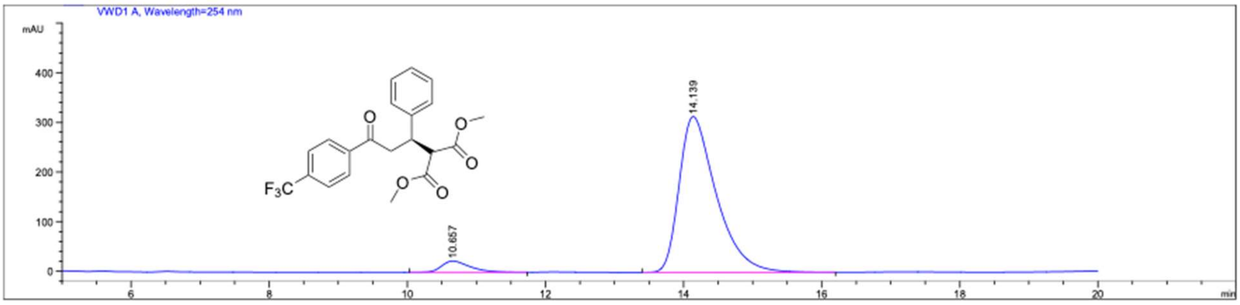

	Ret. Time (min)	Peak Area (mAU *min)	Peak Height (mAu)	Area %
1	10.657	635.5	22.5	5.152
2	14.139	11700.4	313.6	94.848

Eluent: Hexane/ 2-propanol 3:2; 1.0 mL/min

(-)-Dimethyl 2-(3-(2-methoxyphenyl)-3-oxo-1-phenylpropyl)malonate (6o)

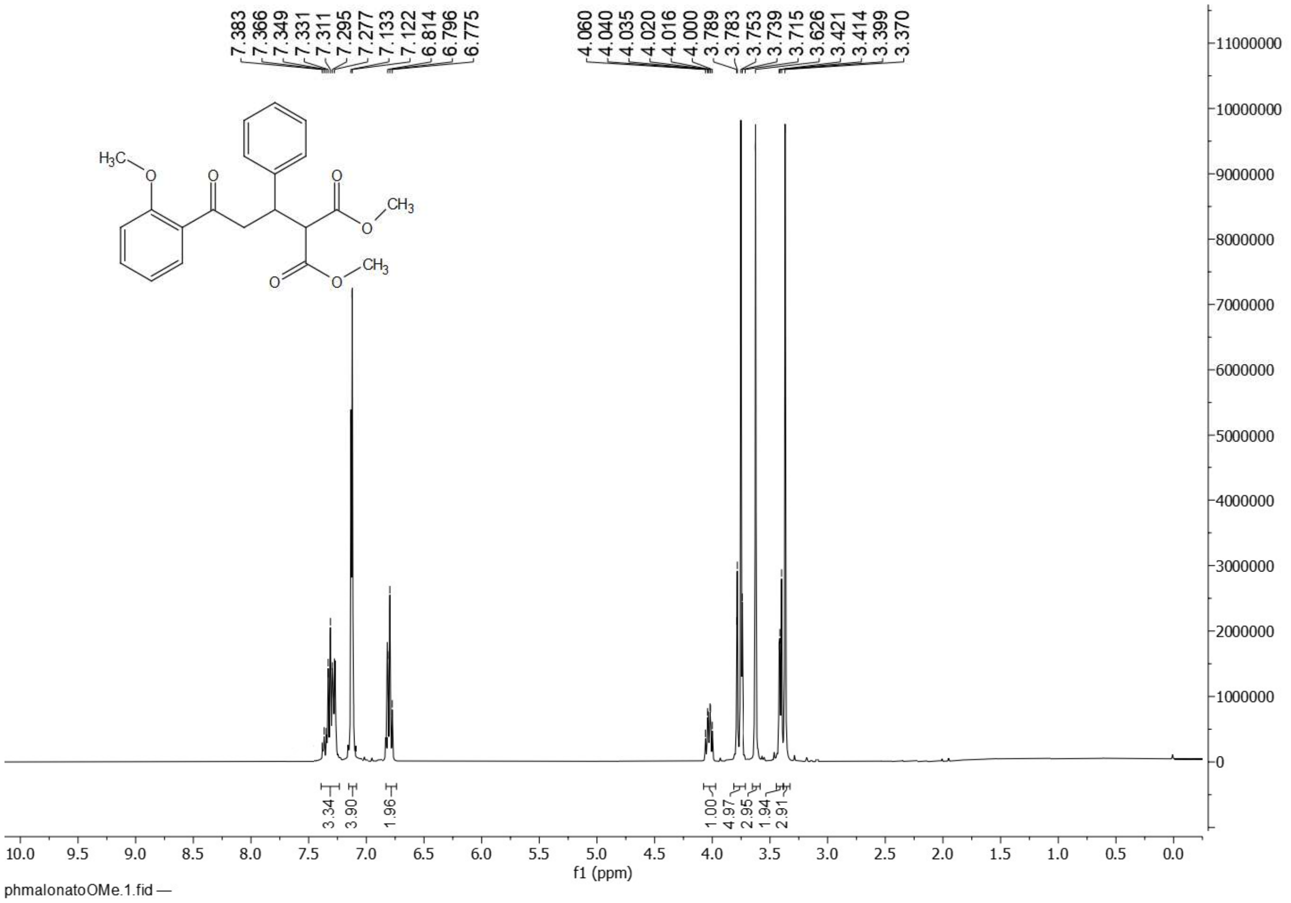

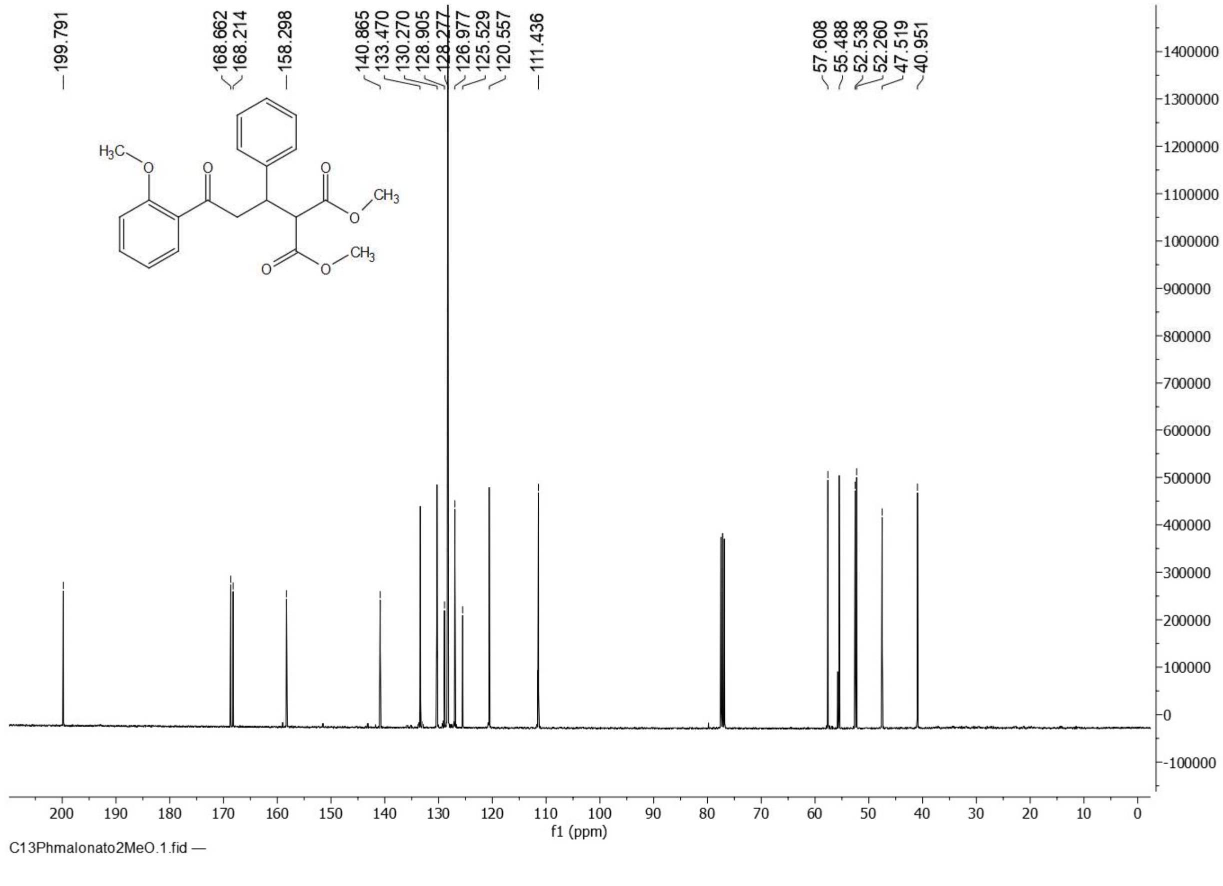

(*rac*)-Dimethyl 2-(3-(2-methoxyphenyl)-3-oxo-1-phenylpropyl)malonate (6o)

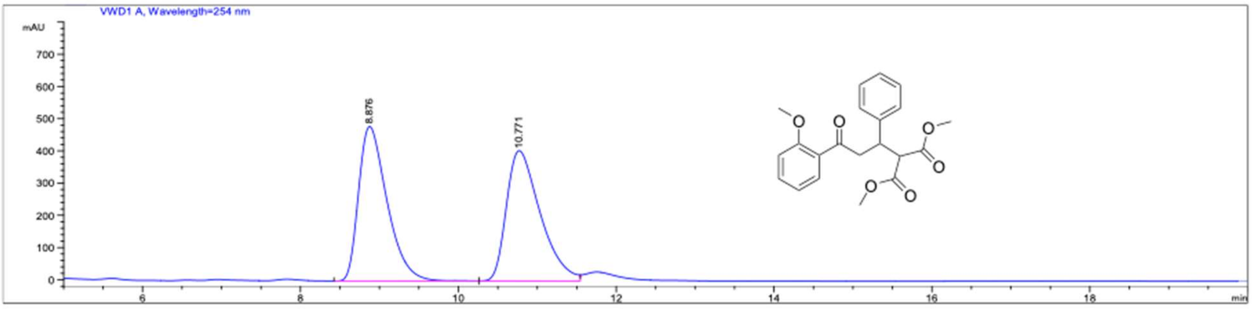

	Ret. Time (min)	Peak Area (mAU *min)	Peak Height (mAu)	Area %
1	8.876	12002.6	479.3	49.959
2	10.771	12022.3	404.4	50.041

(-)-Dimethyl 2-(3-(2-methoxyphenyl)-3-oxo-1-phenylpropyl)malonate (6o)

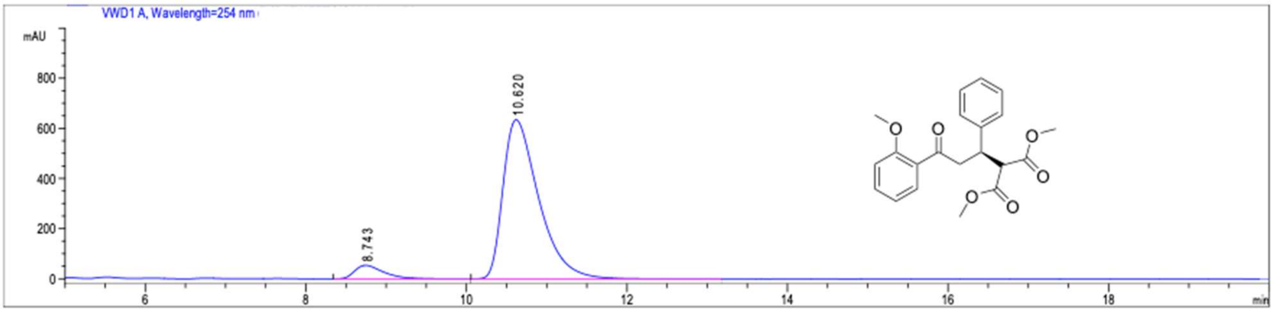

	Ret. Time (min)	Peak Area (mAU *min)	Peak Height (mAu)	Area %
1	8.743	1364.6	54.1	6.496
2	10.620	19644.2	635.1	93.504

Eluent: Hexane/ 2-propanol 3:2; 1.0 mL/min

(-)-Dimethyl 2-(1-(4-chlorophenyl)-3-oxo-3-(*m*-tolyl)propyl)malonate (6p)

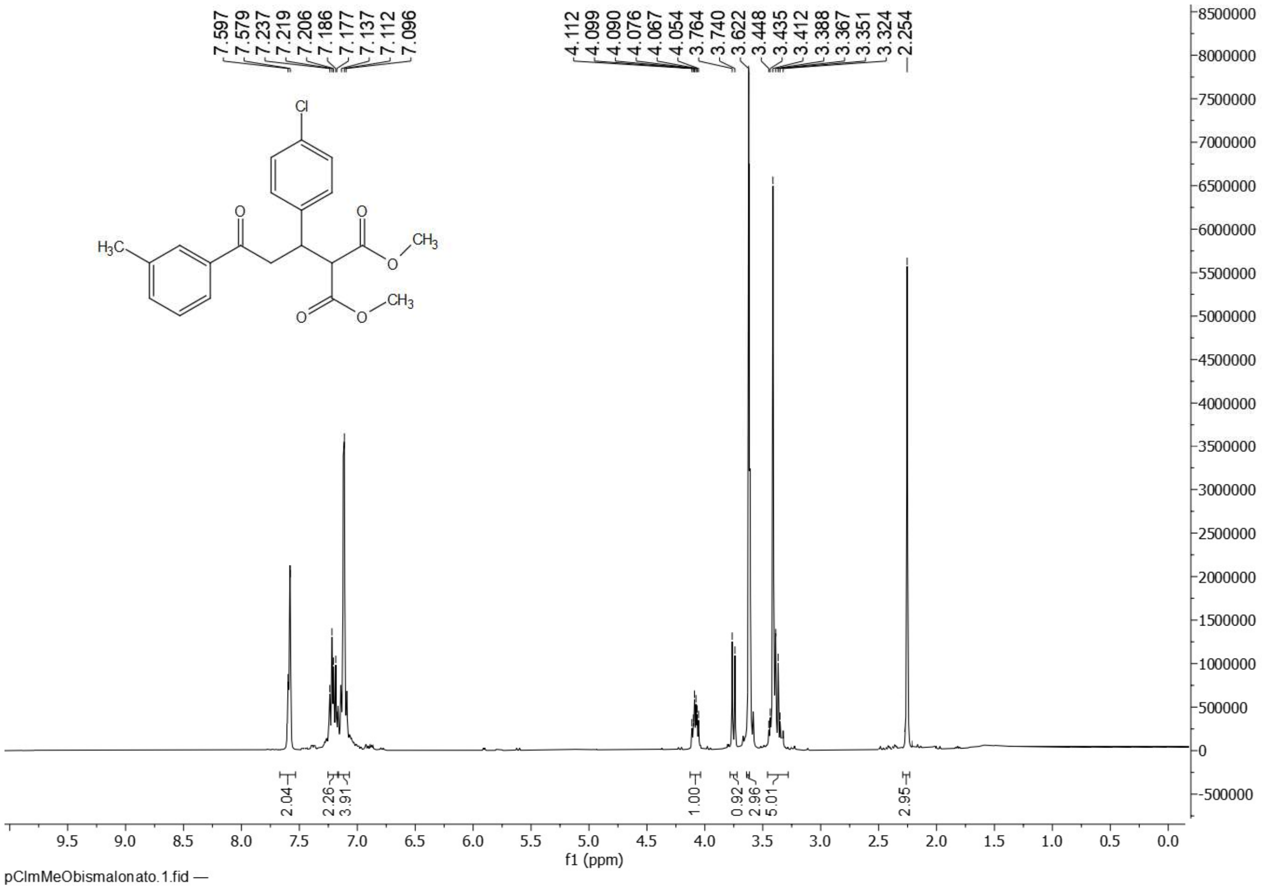

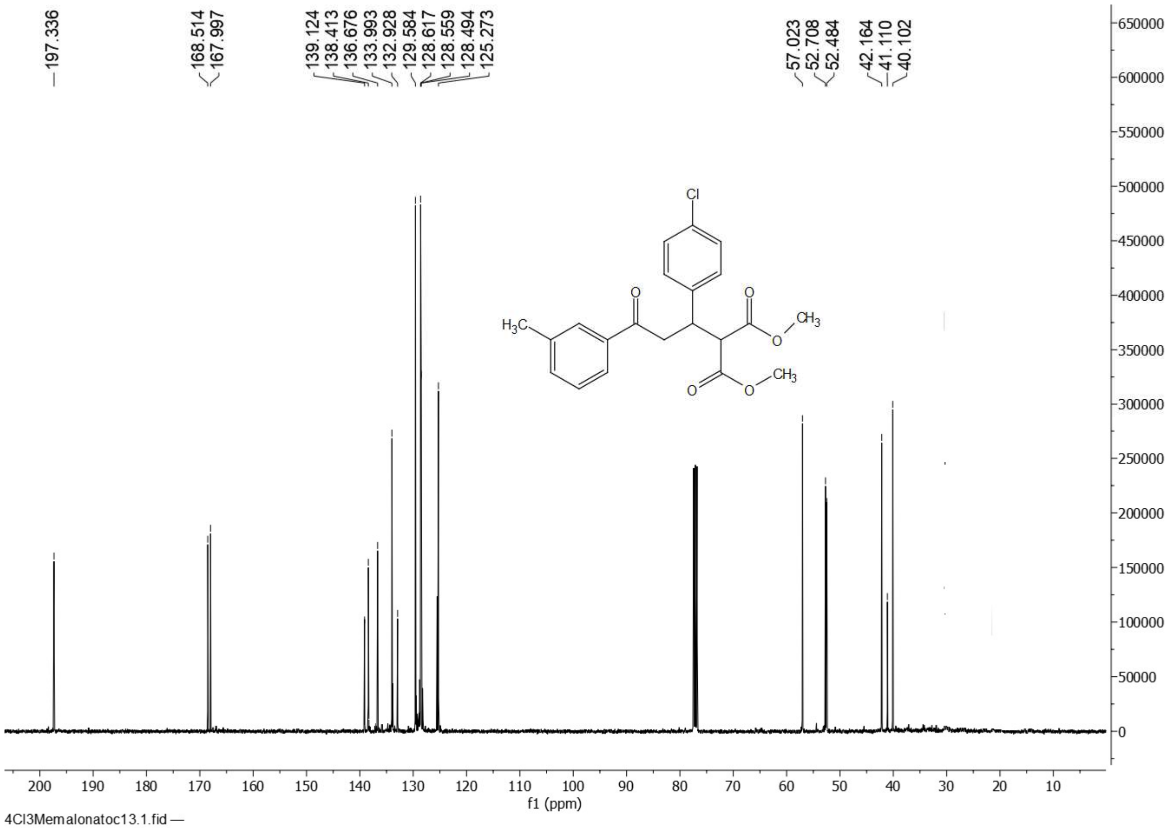

(*rac*)-Dimethyl 2-(1-(4-chlorophenyl)-3-oxo-3-(*m*-tolyl)propyl)malonate (6p)

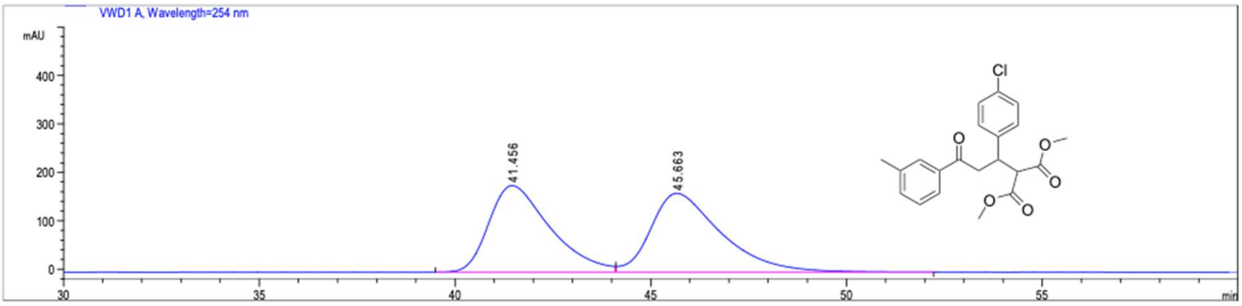

	Ret. Time (min)	Peak Area (mAU *min)	Peak Height (mAu)	Area %
1	41.456	19378.4	178.5	48.979
2	45.663	20186.0	162.5	51.021

(-) Dimethyl 2-(1-(4-chlorophenyl)-3-oxo-3-(*m*-tolyl)propyl)malonate (6p)

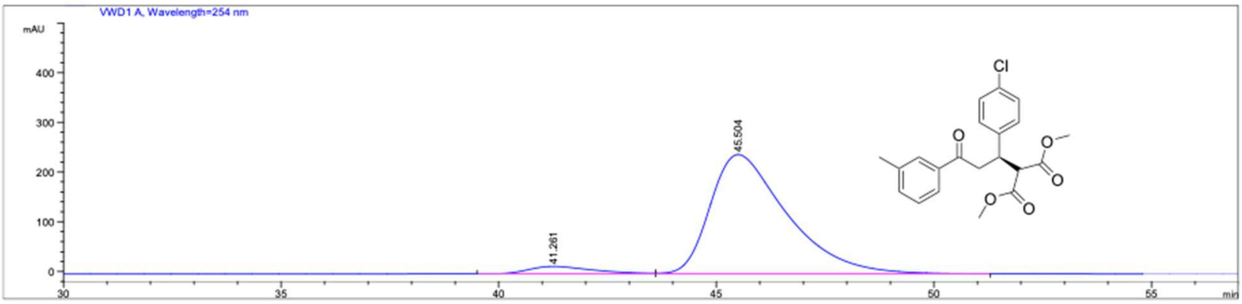

	Ret. Time (min)	Peak Area (mAU *min)	Peak Height (mAu)	Area %
1	41.261	1552.7	15.0	5.012
2	45.504	29426.1	240.1	94.988

Eluent: Hexane/ 2-propanol 4:1; 1.0 mL/min

(-)-Dimethyl 2-(1-(4-nitrophenyl)-3-oxo-3-(*m*-tolyl)propyl)malonate (6q)

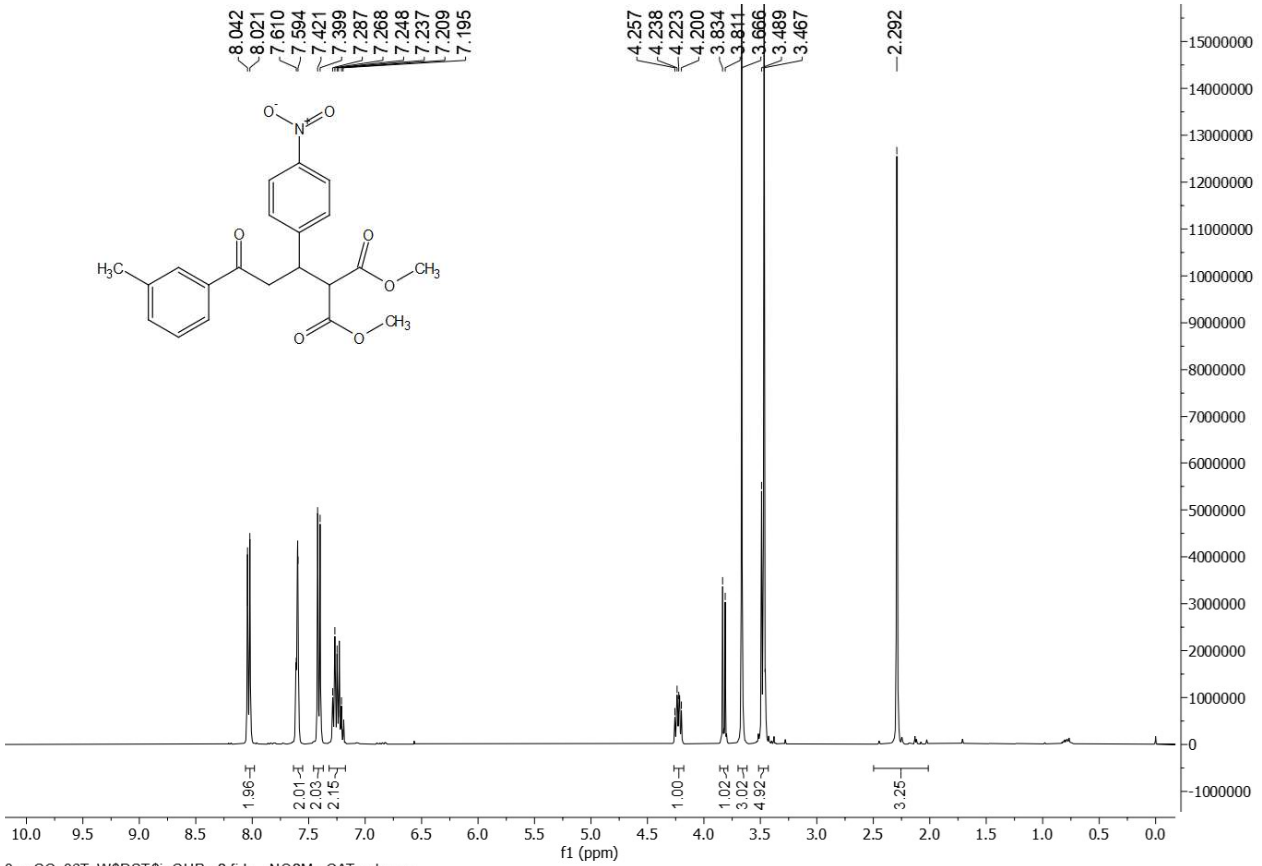

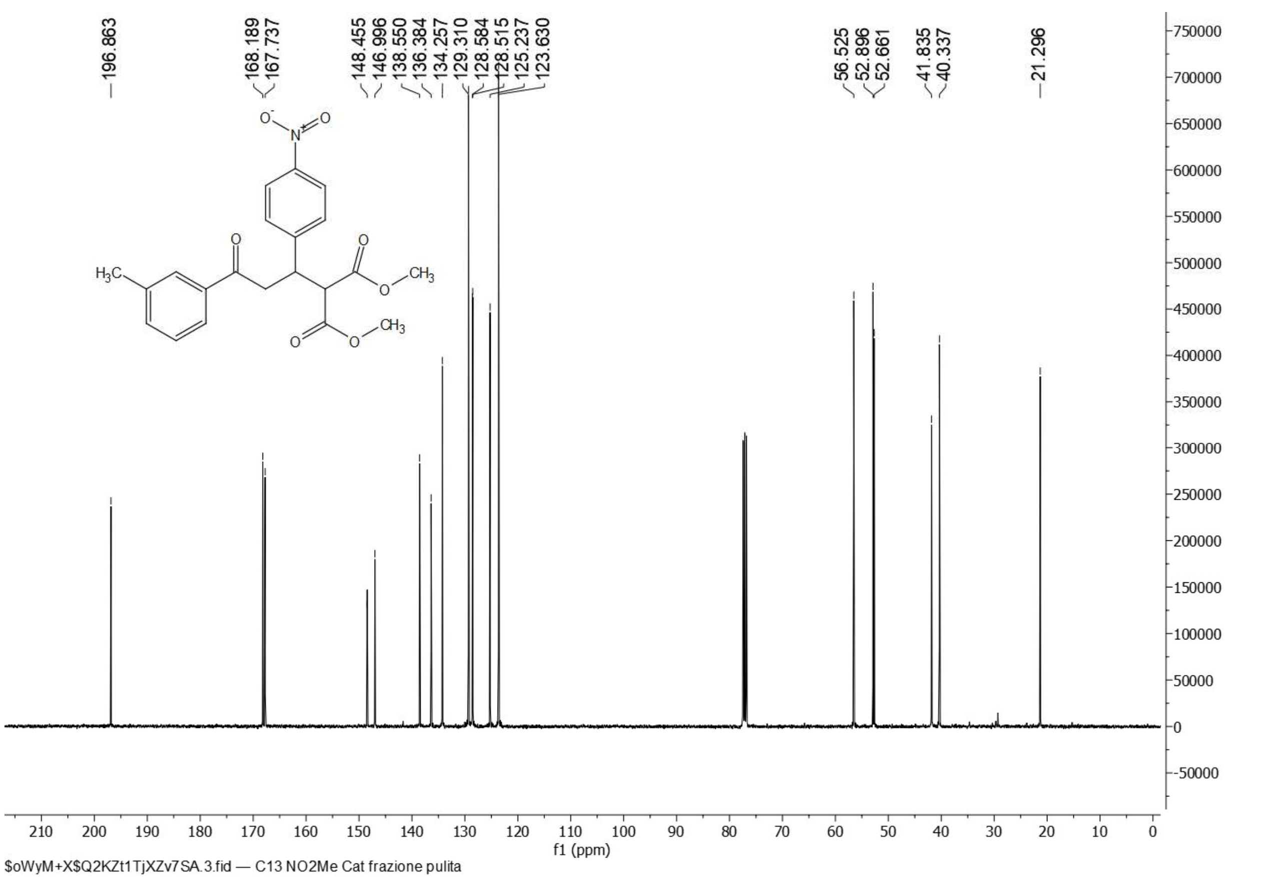

(*rac*)-Dimethyl 2-(1-(4-nitrophenyl)-3-oxo-3-(*m*-tolyl)propyl)malonate (6q)

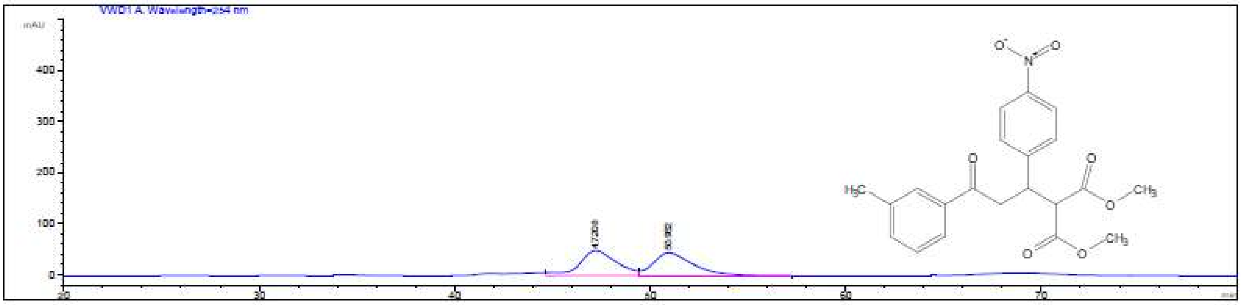

	Ret. Time (min)	Peak Area (mAU *min)	Peak Height (mAu)	Area %
1	47.208	6502.3	49.0	50.736
2	50.962	6313.6	45.1	49.264

(-)-Dimethyl 2-(1-(4-nitrophenyl)-3-oxo-3-(*m*-tolyl)propyl)malonate (6q)

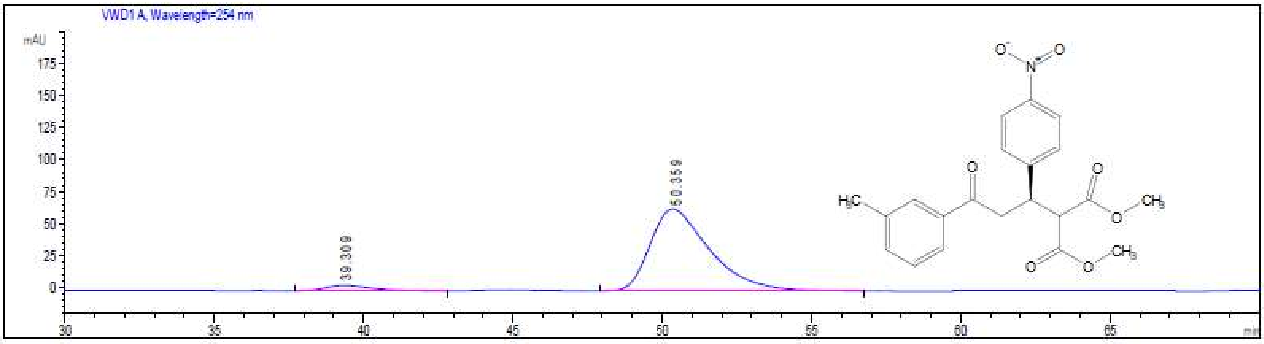

	Ret. Time (min)	Peak Area (mAU *min)	Peak Height (mAu)	Area %
1	39.208	482.4	4.4	5.140
2	50.359	8902.9	63.9	94.860

Eluent: Hexane/ 2-propanol 4:1; 1.0 mL/min

(-)-Dimethyl 2-(3-oxo-1-(*p*-tolyl)- 3-(4-trifluoromethyl)phenyl)propyl)malonate (6r)

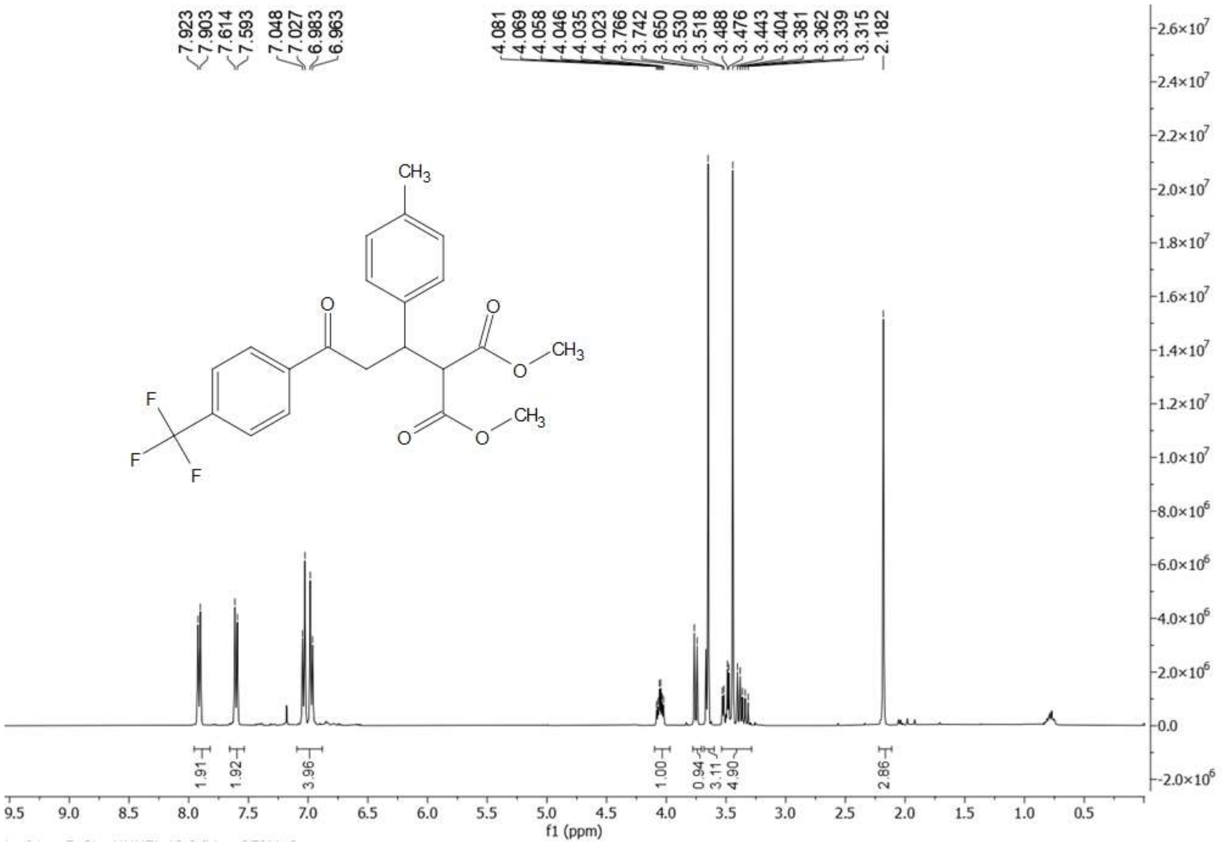

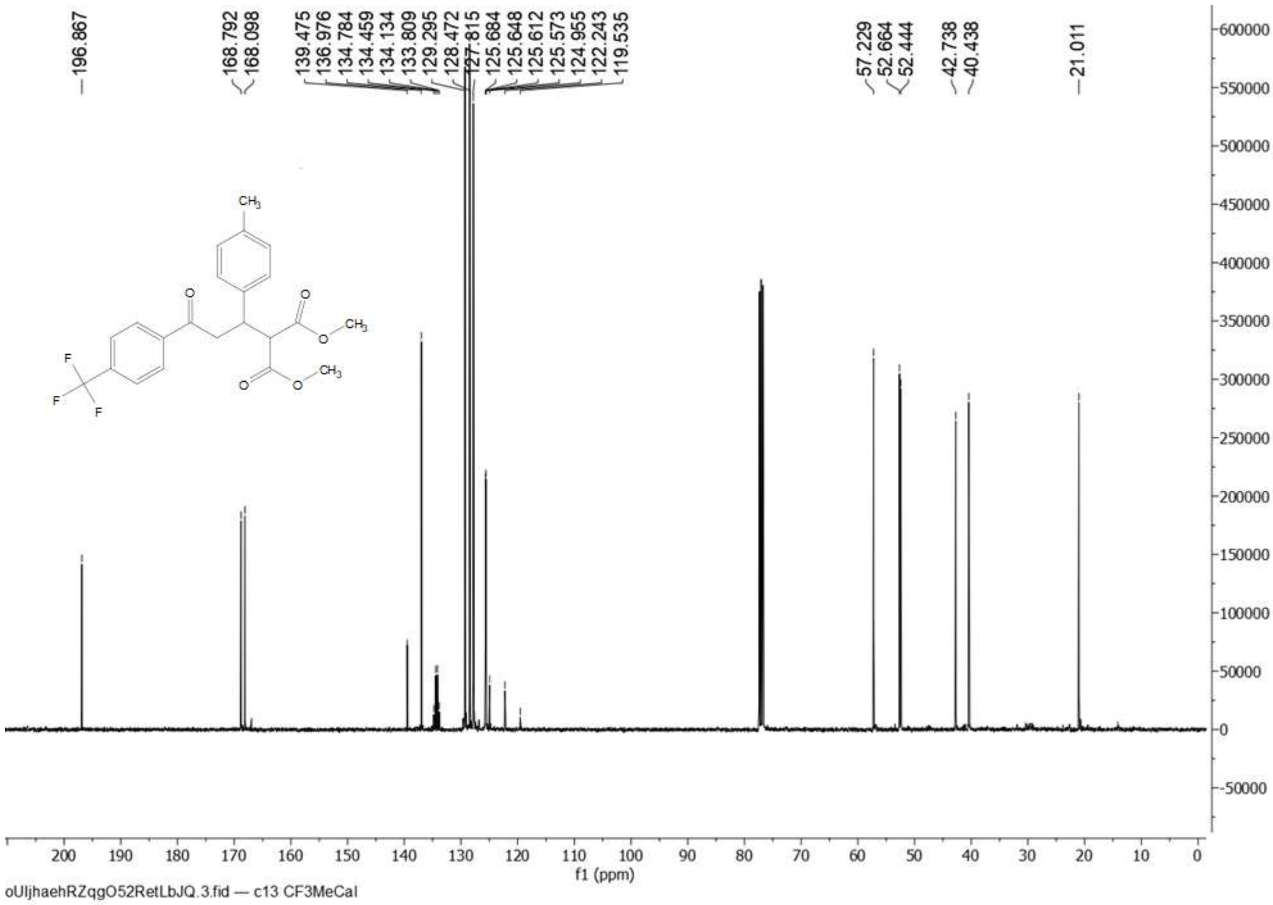

(*rac*)-Dimethyl 2-(3-oxo-1-(*p*-tolyl)- 3-(4-trifluoromethyl)phenyl)propyl)malonate (6r)

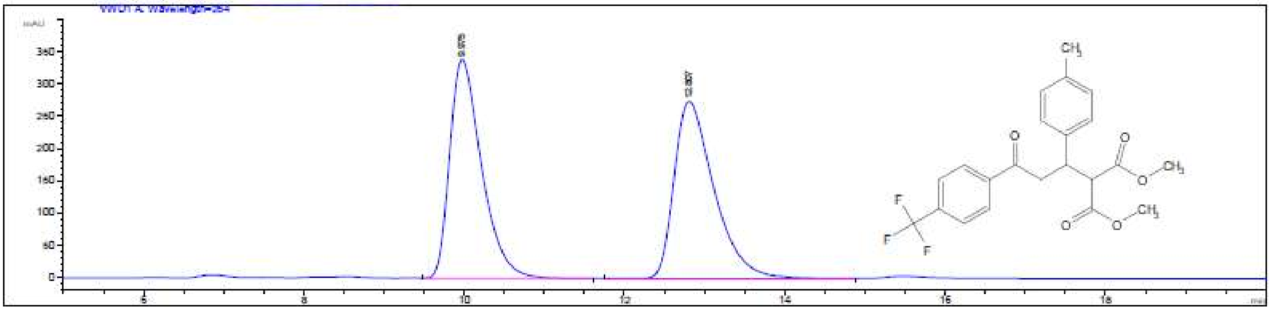

	Ret. Time (min)	Peak Area (mAU *min)	Peak Height (mAu)	Area %
1	9.975	9354.7	338.8	49.688
2	12.807	9472.3	274.7	50.312

(-)-Dimethyl 2-(3-oxo-1-(*p*-tolyl)- 3-(4-trifluoromethyl)phenyl)propyl)malonate (6r)

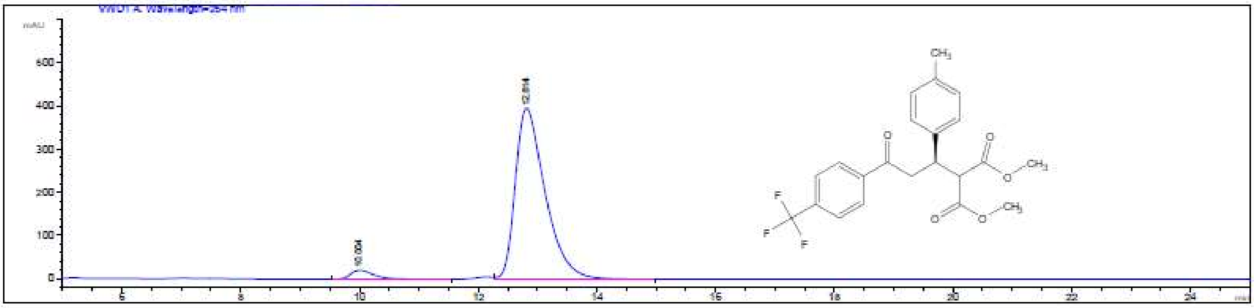

	Ret. Time (min)	Peak Area (mAU *min)	Peak Height (mAu)	Area %
1	10.004	544.2	20.2	3.792
2	12.814	13808.8	395.6	96.208

Eluent: Hexane/ 2-propanol 4:1; 1.0 mL/min

(*R*)-Dimethyl 2-(3-(4-chlorophenyl)-3-oxo-1-phenylpropyl)malonate (6s)

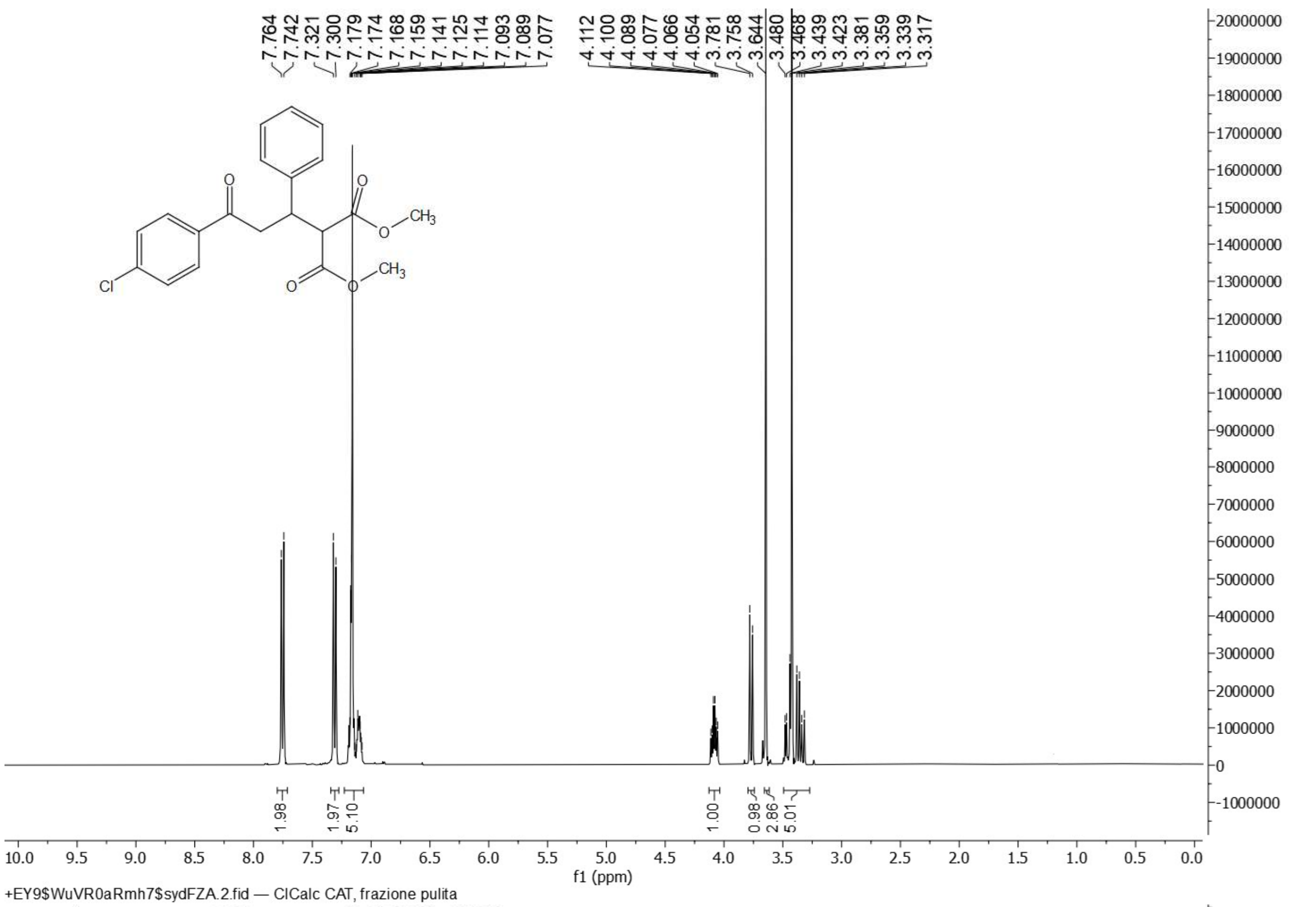

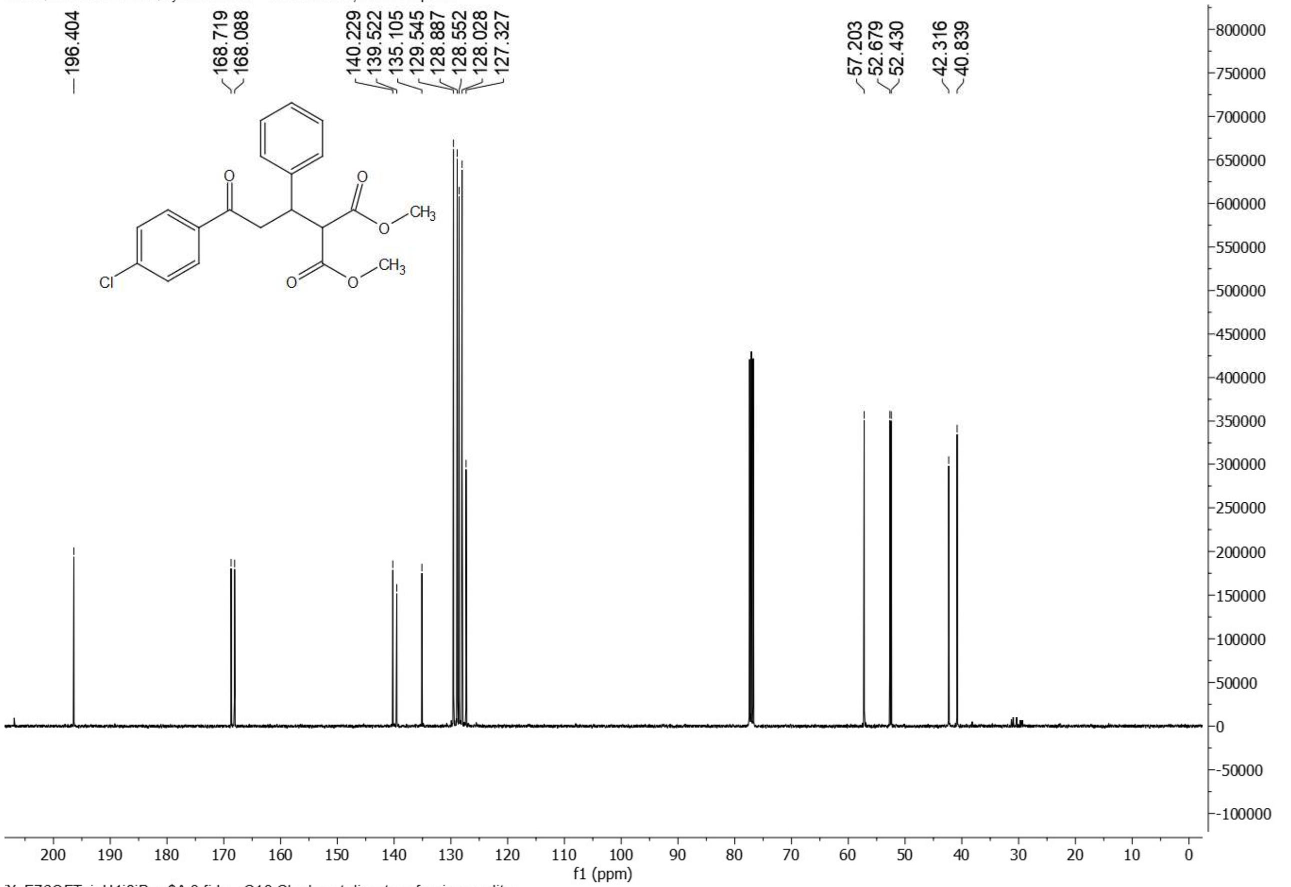

(*rac*)-Dimethyl 2-(3-(4-chlorophenyl)-3-oxo-1-phenylpropyl)malonate (6s)

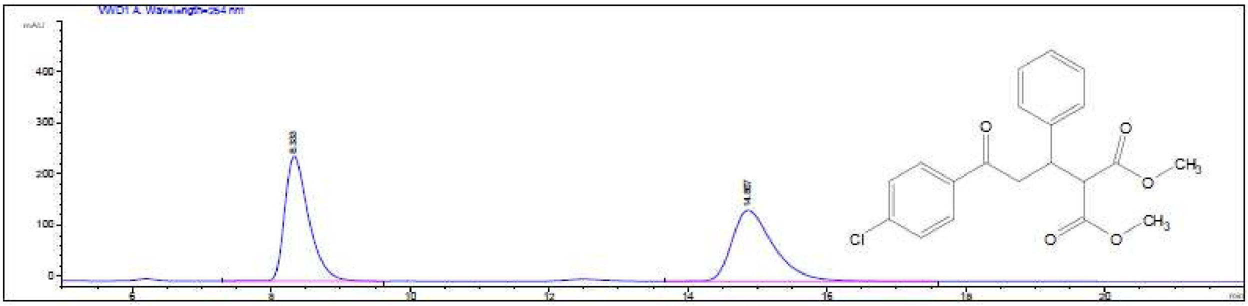

	Ret. Time (min)	Peak Area (mAU *min)	Peak Height (mAu)	Area %
1	8.333	5864.7	244.5	50.420
2	14.867	5800.6	139.1	49.580

(*R*)-Dimethyl 2-(3-(4-chlorophenyl)-3-oxo-1-phenylpropyl)malonate (6s)

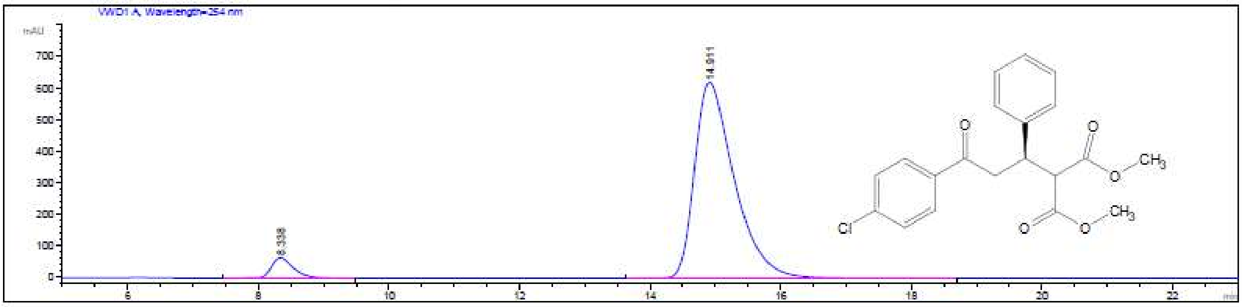

	Ret. Time (min)	Peak Area (mAU *min)	Peak Height (mAu)	Area %
1	8.338	1513.4	64.2	5.460
2	14.911	262707.7	620.4	94.540

Eluent: Hexane/ 2-propanol 3:2; 1.0 mL/min

(*R*)-Dimethyl 2-(3-(4-nitrophenyl)-3-oxo-1-phenylpropyl)malonate (6t)

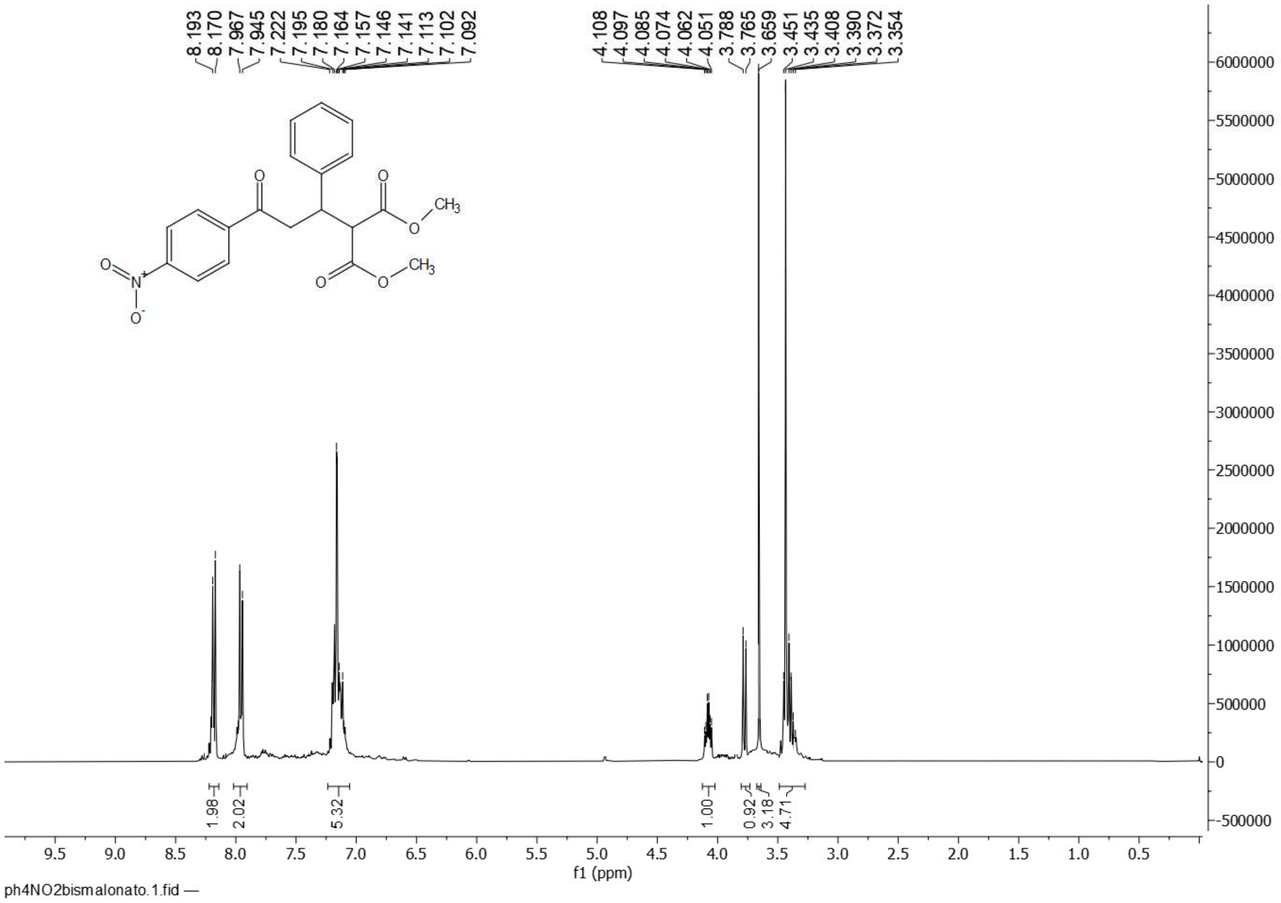

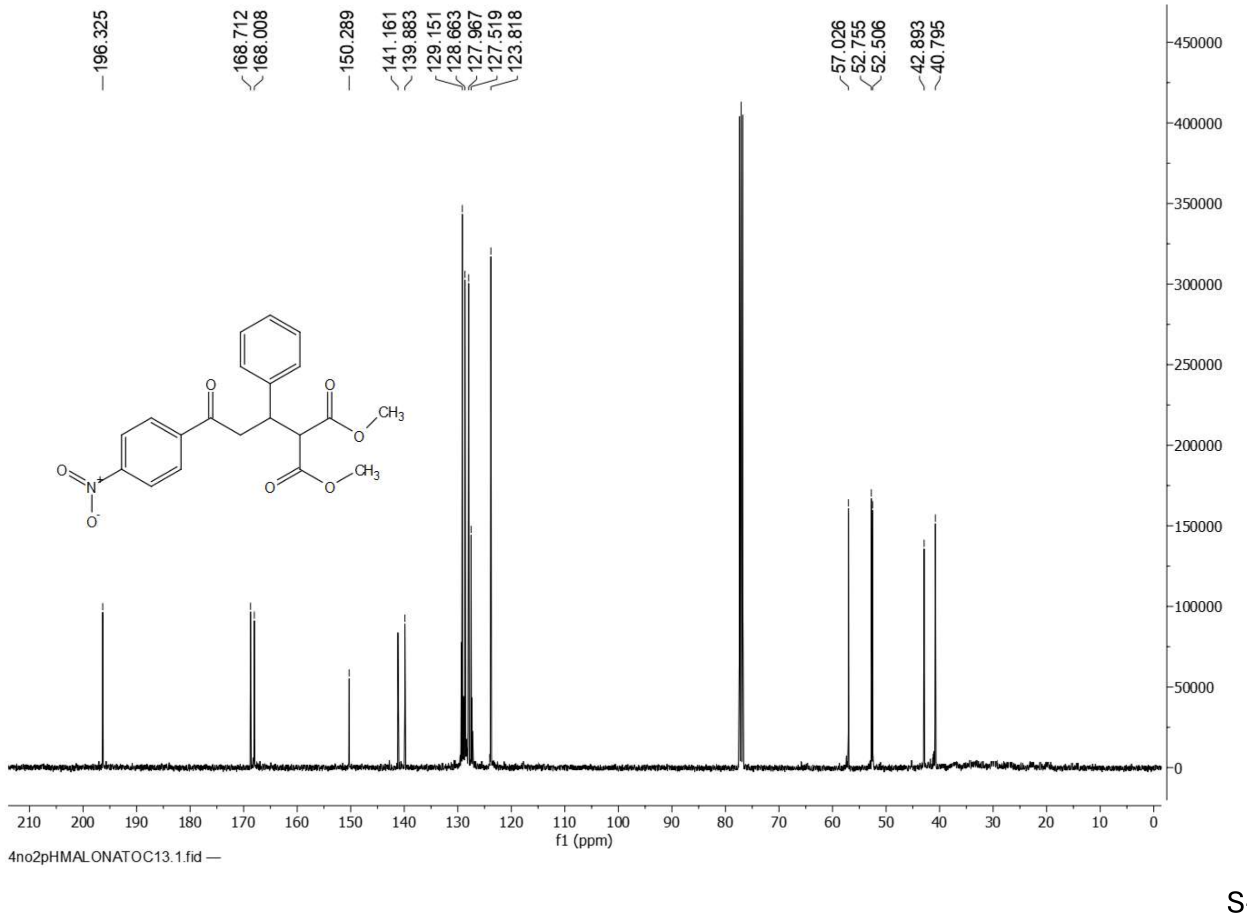

(*rac*)-Dimethyl 2-(3-(4-nitrophenyl)-3-oxo-1-phenylpropyl)malonate (6t)

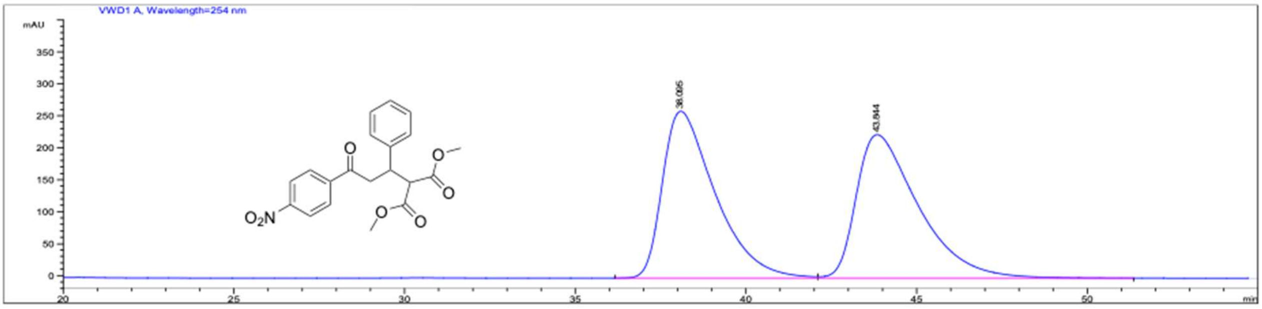

	Ret. Time (min)	Peak Area (mAU *min)	Peak Height (mAu)	Area %
1	38.095	28029.5	260.9	49.721
2	43.844	28344.6	224.4	50.279

(*R*)-Dimethyl 2-(3-(4-nitrophenyl)-3-oxo-1-phenylpropyl)malonate (6t)

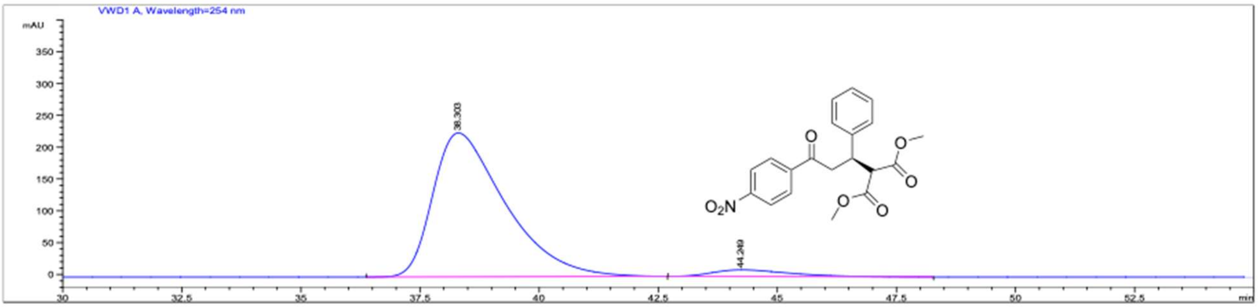

	Ret. Time (min)	Peak Area (mAU *min)	Peak Height (mAu)	Area %
1	38.303	23925.4	226.2	95.266
2	44.249	1188.8	10.5	4.734

Eluent: Hexane/ 2-propanol 3:2; 1.0 mL/min

(-)-Dimethyl 2-(4-oxohexan-2-yl)malonate (6u)

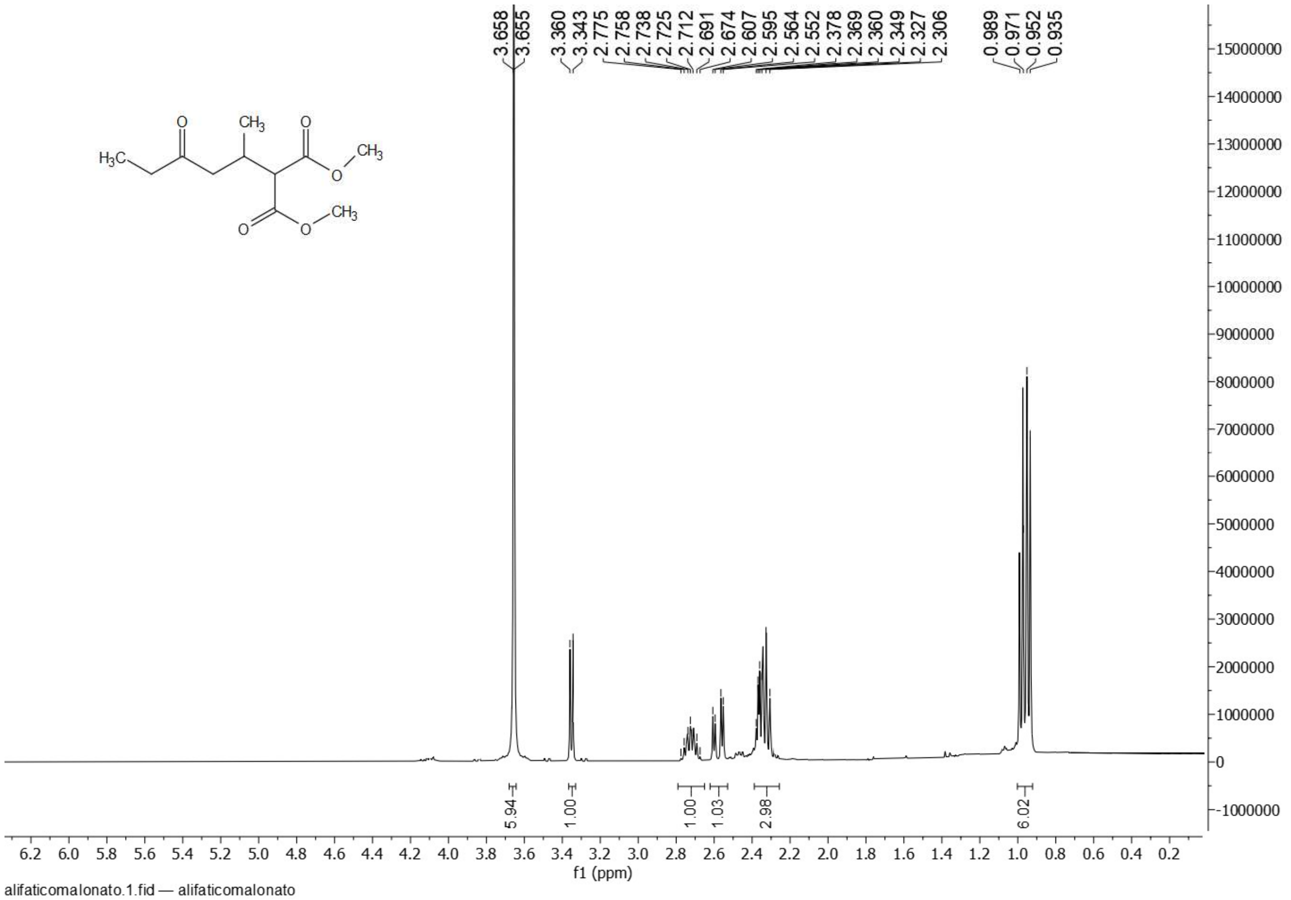

alifaticomalonato.1.fid — alifaticomalonato

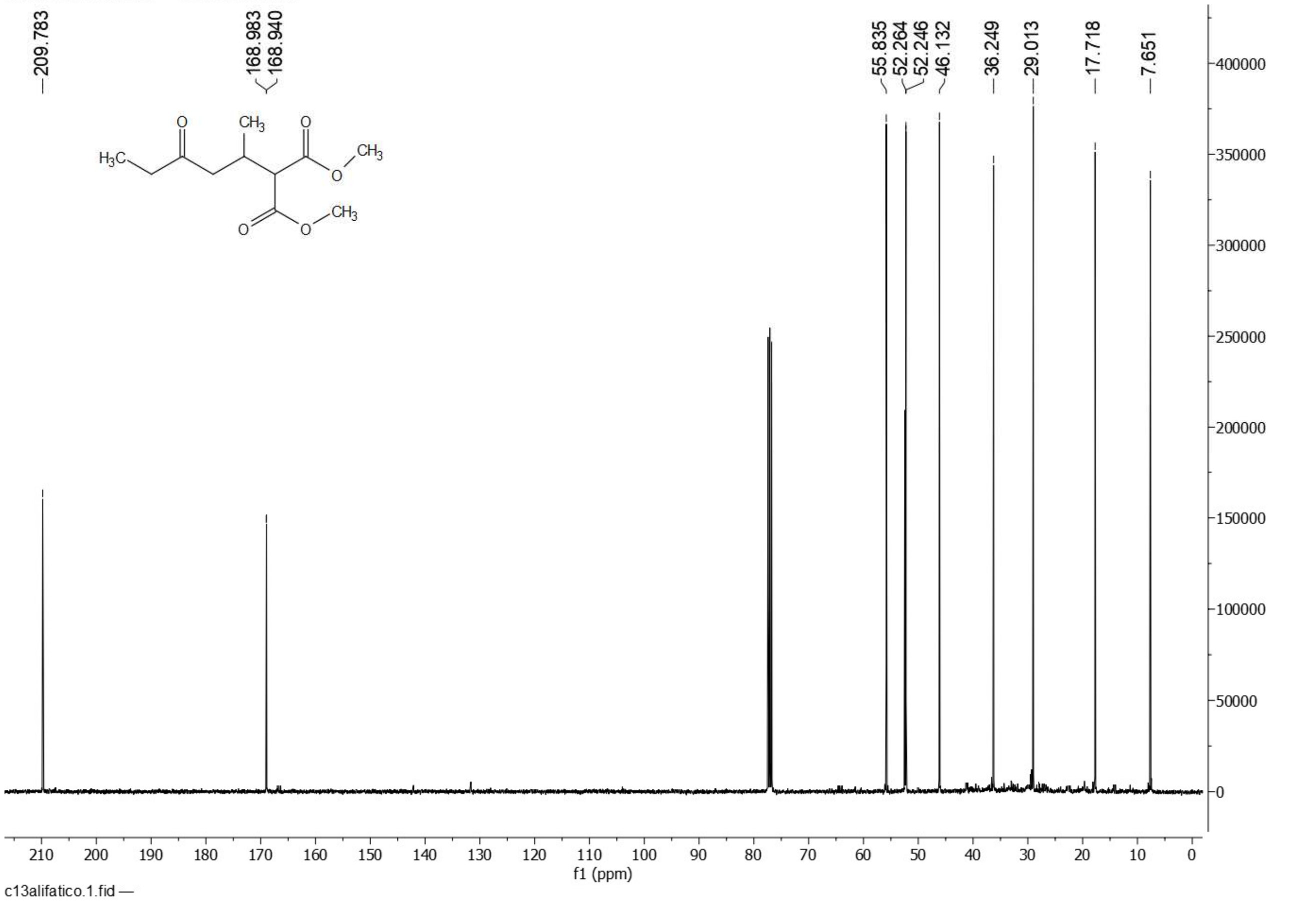

c13alifatico.1.fid —

(*rac*)-Dimethyl 2-(4-oxohexan-2-yl)malonate (6u)

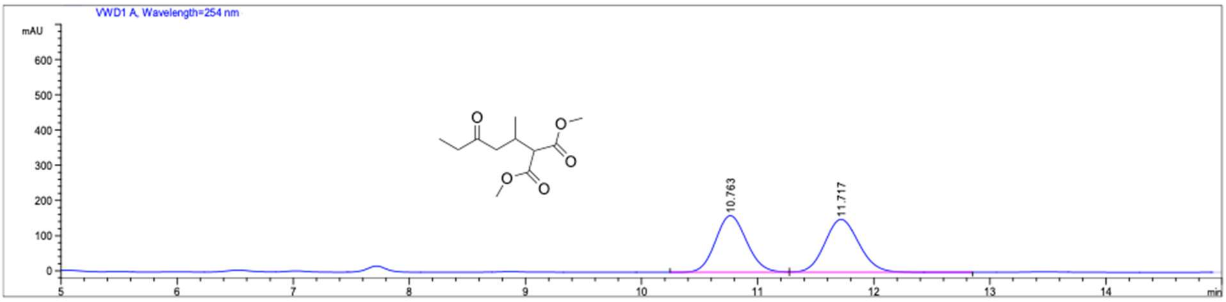

	Ret. Time (min)	Peak Area (mAU *min)	Peak Height (mAu)	Area %
1	10.763	3002.3	160.7	49.374
2	11.717	3078.4	150.3	50.626

(-)-Dimethyl 2-(4-oxohexan-2-yl)malonate (6u)

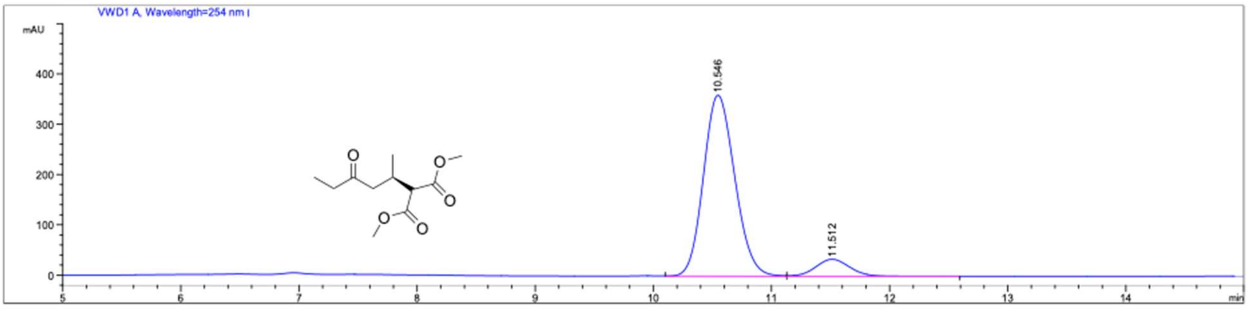

	Ret. Time (min)	Peak Area (mAU *min)	Peak Height (mAu)	Area %
1	10.546	6638.9	359.9	90.774
2	11.717	674.8	33.7	9.226

Eluent: Hexane/ 2-propanol 9:1; 1.0 mL/min

(*R*)-Dimethyl 2-(3-oxocyclopentyl)malonate (6v)

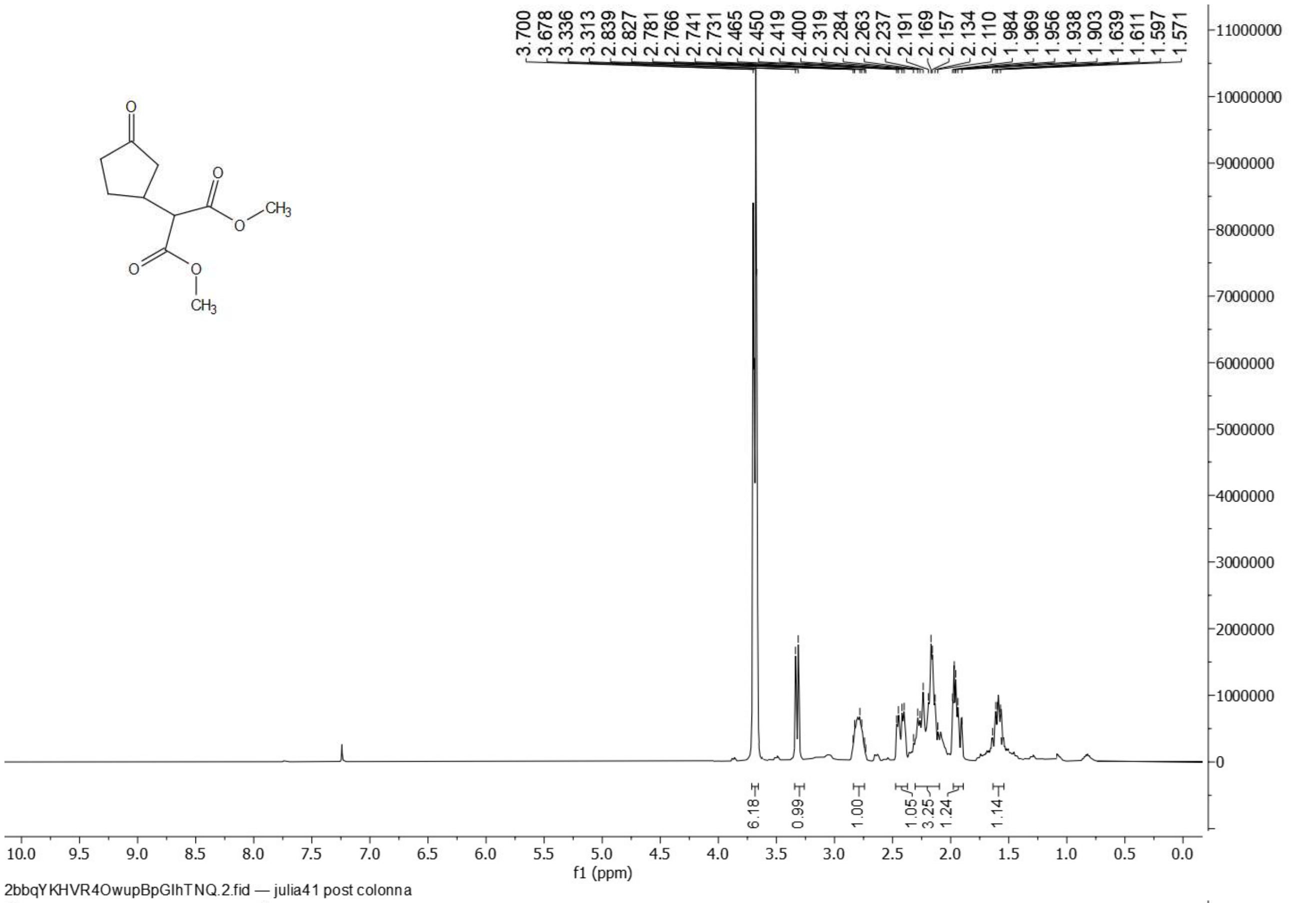

2bbqYKHVR4OwupBpGlhTNQ.2.fid — julia41 post colonna

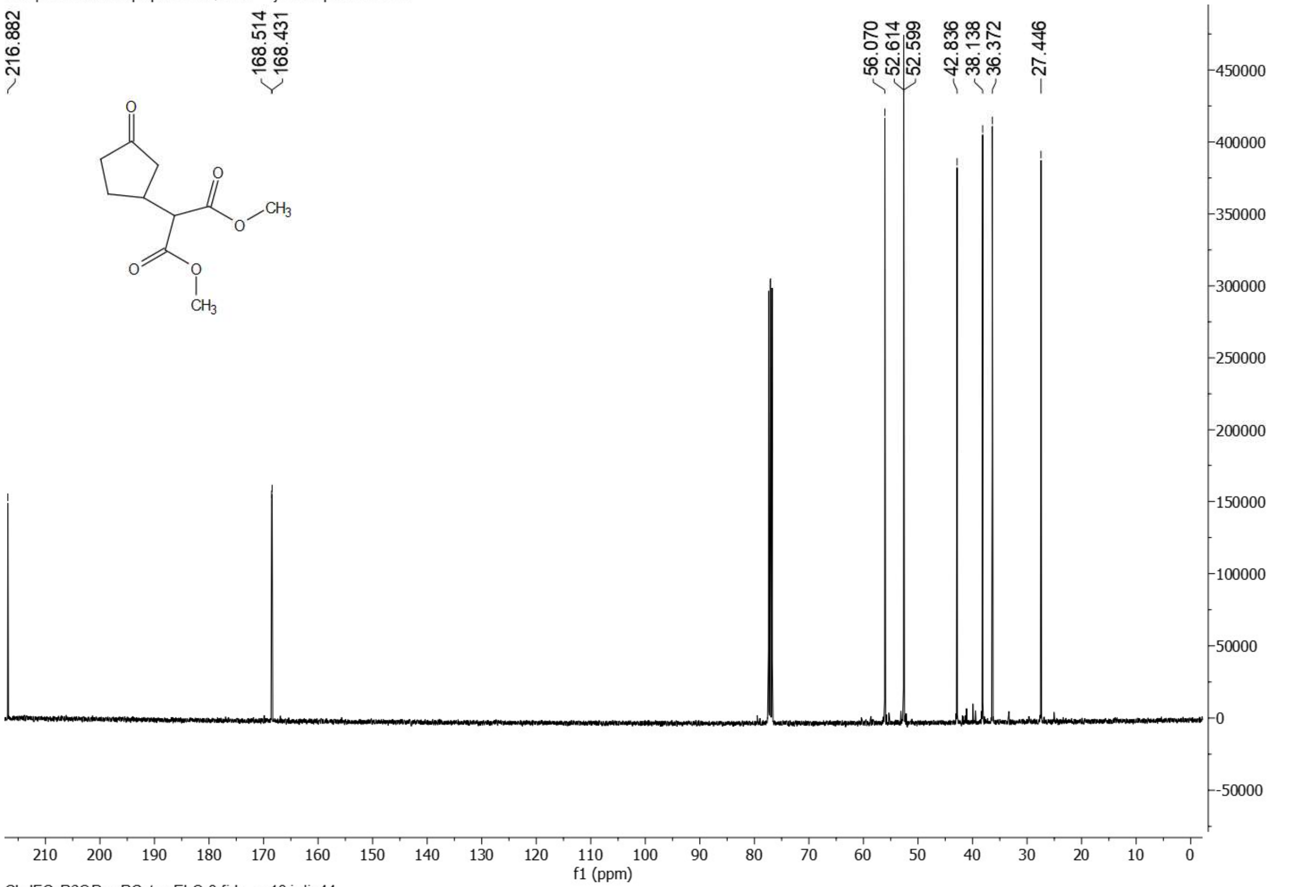

CLdFCxP6QDywPGxtrwEbQ.3.fid — c13 julia44

(*rac*)-Dimethyl 2-(3-oxocyclopentyl)malonate (6v)

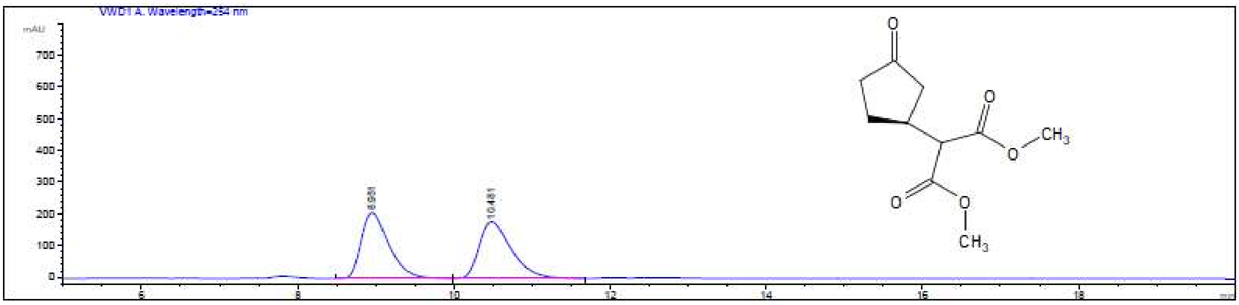

	Ret. Time (min)	Peak Area (mAU *min)	Peak Height (mAu)	Area %
1	8.951	5138.5	207.1	49.825
2	10.481	5174.6	179.2	50.175

(*R*)-Dimethyl 2-(3-oxocyclopentyl)malonate (6v)

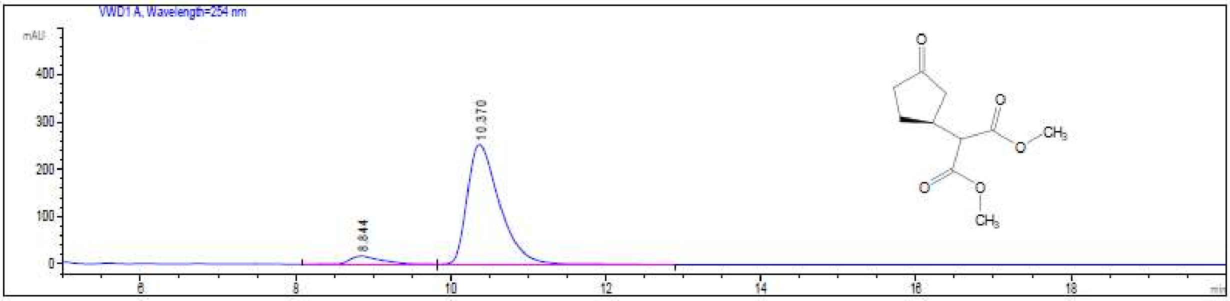

	Ret. Time (min)	Peak Area (mAU *min)	Peak Height (mAu)	Area %
1	8.884	489.6	17.7	6.262
2	10.370	7328.7	253.3	93.738

Eluent: Hexane/ 2-propanol 9:1; 1.0 mL/min

(*R*)-Dimethyl 2-(3-oxocyclohexyl)malonate (6w)

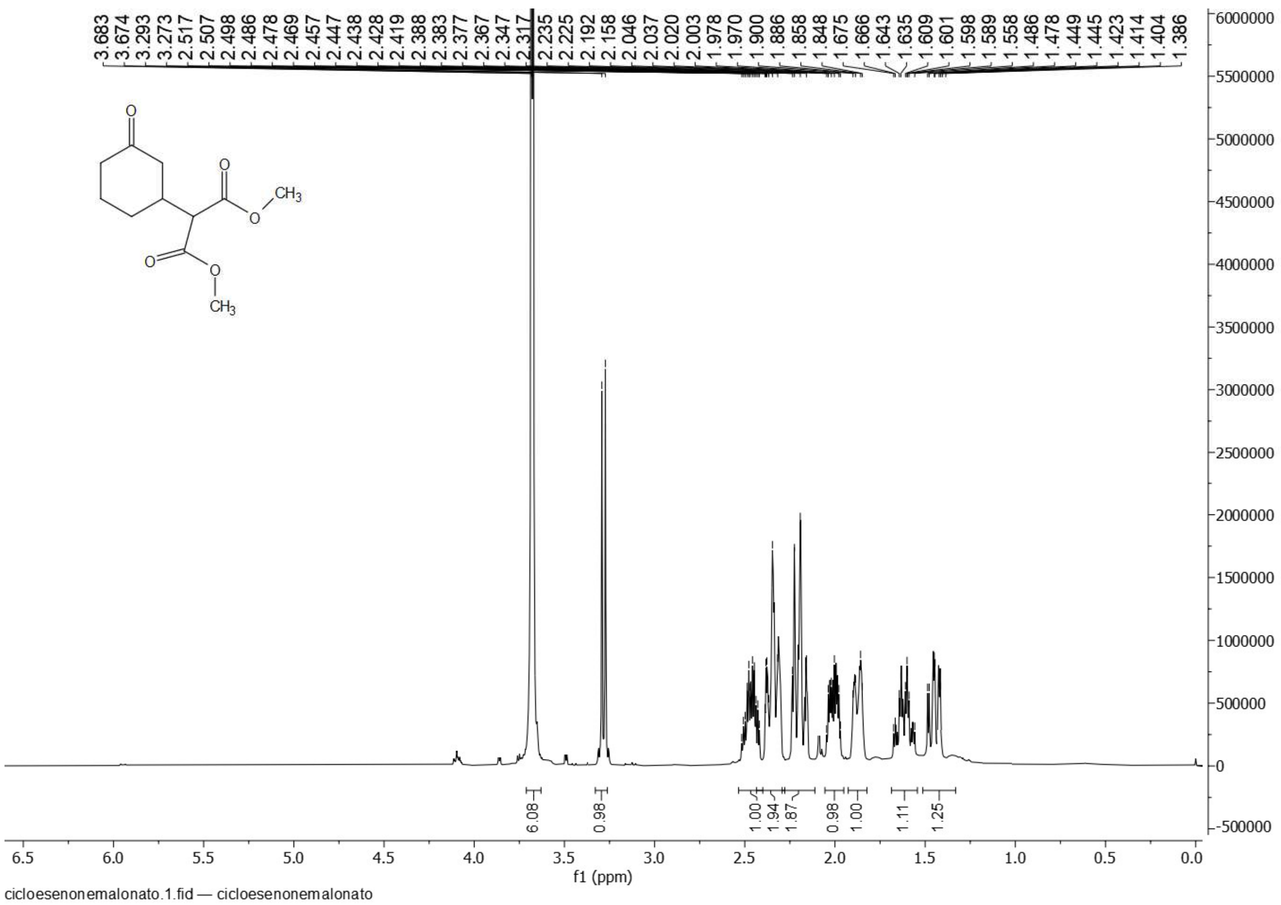

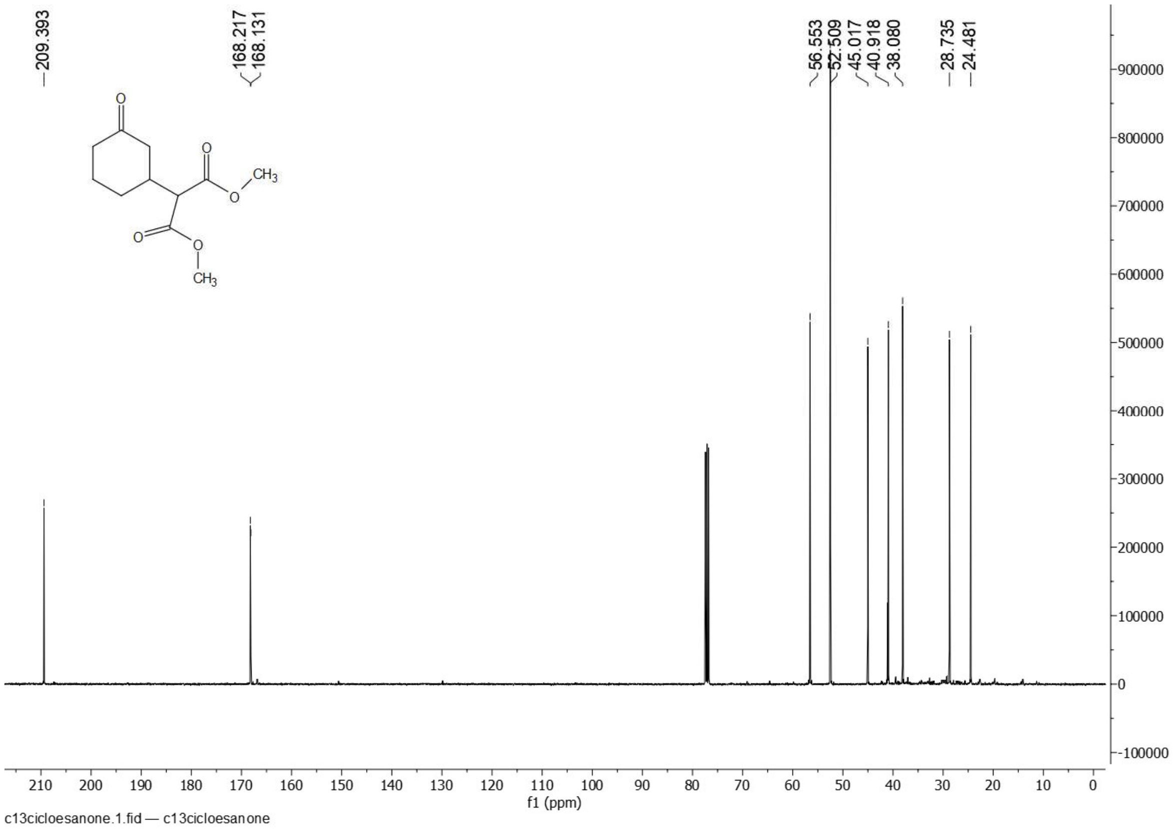

(*rac*)-Dimethyl 2-(3-oxocyclohexyl)malonate (6w)

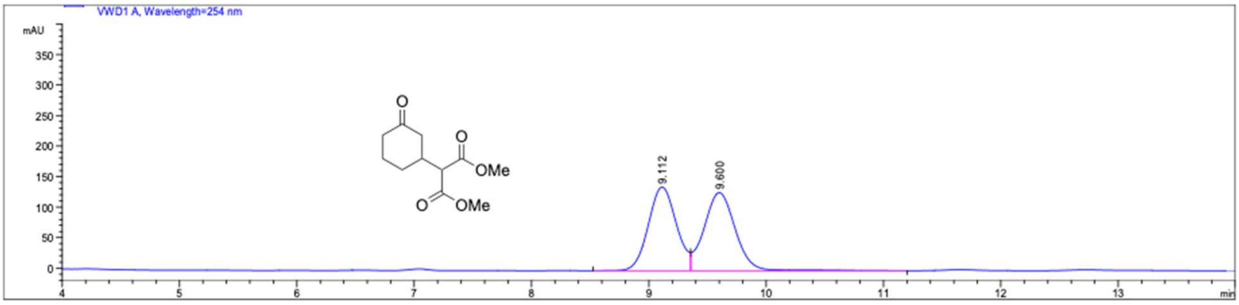

	Ret. Time (min)	Peak Area (mAU *min)	Peak Height (mAu)	Area %
1	9.112	2276.8	137.0	48.954
2	9.600	2374.1	128.2	51.046

(*R*)-Dimethyl 2-(3-oxocyclohexyl)malonate (6w)

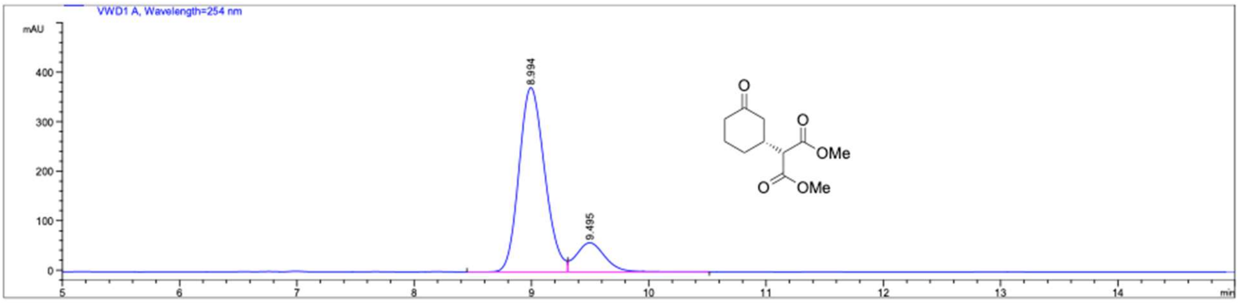

	Ret. Time (min)	Peak Area (mAU *min)	Peak Height (mAu)	Area %
1	8.994	5673.5	372.3	85.081
2	9.495	994.8	58.8	14.919

Eluent: Hexane/ 2-propanol 9:1; 1.0 mL/min

Computational method.

The structures of the reactants, intermediates and transition states have been optimized by using the density functional method (DFT)[1] with the functional M06-2X[2,3] and the basis sets def2-SVP with diffuse functions on C and O atoms.[4] The nature of the critical points was characterized by using vibrational analysis[5] which also furnished the Zero Point Energies (ZPE) and entropies for the calculations of the Free Energies. These have been converted from the gas phase to the 1 M standard state at 1 atm and 298.15, 275.15 and 253.15 K.[6] The solvent effects (methanol) were introduced in all calculations using the universal solvation model (SMD) by Truhalr *et al.*[7] For sodium malonate, TS-s and TS-a (see Table S-1), The rate constants of the reactions are calculated using canonical transition state theory.[8]

$$k = \sigma \bullet (k_B \bullet T / h_P) \bullet (RT)^{n-1} \bullet \exp(-\Delta G^{\ddagger} / RT)$$

in which σ is the symmetry number, k_B the Boltzmann constant, h_P the Planck constant, n is 1 for for monomolecular reactions and 2 for bimolecular reactions.

The calculations were performed by the quantum package Gaussian 16-A.03[9] The figures were obtained using the graphical program Molden.[10]

[1] Parr, R.G. Density Functional Theory of Atoms and Molecules, in: Horizons Quantum Chem., Springer Netherlands, **1980**: pp. 5–15. DOI:10.1007/978-94-009-9027-2_2.

[2] Zhao, Y.; Truhlar, D.G. The M06 suite of density functionals for main group thermochemistry, thermochemical kinetics, noncovalent interactions, excited states, and transition elements: two new functionals and systematic testing of four M06-class functionals and 12 other functionals. *Theor. Chem. Acc.* **2008**, *120*, 215–241. DOI: 10.1007/s00214-007-0310-x.

[3] Zhao, Y.; Truhlar, D.G. Density functionals with broad applicability in chemistry. *Acc. Chem. Res.* **2008**,*41*,157–167. DOI: 10.1021/ar700111a.

[4] Schaefer, A.; Horn, H.; Ahlrichs, R. Fully optimized contracted Gaussian basis sets for atoms Li to Kr. *J. Chem. Phys.***1992**, *97*, 2571–2577. DOI: 10.1063/1.463096.

[5] Foresman, J.; Frisch, A. Exploring chemistry with electronic structure methods, **1996**, Gaussian Inc, Pittsburgh, PA, 1996, http://gaussian.com/expchem3/(accessed June 4, 2021).

[6] Ribeiro, R.F.; Marenich, A.V.; Cramer, C.J.; Truhlar, D.G. Use of solution-phase vibrational frequencies in continuum models for the free energy of solvation. *J. Phys. Chem. B.* **2011**, *115*, 14556–14562, DOI: 10.1021/jp205508z.

[7] Marenich, A.V.; Cramer, C.J.; Truhlar, D.G. Universal solvation model based on solute electron density and on a continuum model of the solvent defined by the bulk dielectric constant and atomic surface tensions *J. Phys. Chem. B*, **2009**, *113*, 6378–6396. DOI: 10.1021/jp810292n.

[8] Truhlar, D.G.; Garrett, B.C.; Klippenstein, S.J. Current status of transition-state theory *J. Phys. Chem.* **1996**, *100*, 12771–. DOI: 10.1021/jp953748q.

[9] Frisch, M.J.; Trucks, G.W.; Schlegel, H.B.; Scuseria, G.E.; Robb, M.A.; Cheeseman, J.R.; Scalmani, G.; Barone, V.; Petersson, G.A.; Nakatsuji, H.; Li, X.; Caricato, M.; Marenich, A.V.; Bloino, J.; Janesko, B.G.; Gomperts, R.; Mennucci, B.; Hratch, Gaussian 16, Revision A.03, **2016**.

[10] Schaftenaar, G.; Noordik, J.H. Molden: a pre- and post-processing program for molecular and electronic structures. *J. Comput. Aided. Mol. Des.* **2000**, *14*, 123–134. DOI: 10.1023/a:1008193805436.

Schemes, table, pictures and Cartesian coordinates. The uncatalyzed reaction.

In the computational study (Scheme S-1 and Table S-1) we start with the sodium malonate with four explicit molecules of methanol, **4'**. This one bound with a molecule of calcone in one of its two conformation (CO-*syn* **5a-s** or CO-*anti* **5a-s**) molecule releasing on molecule of methanol. The relative two transition structures follow (**TS-s** and **TS-a**) yielding the respective adduct **8a-s** and **8a-a**. The reaction with the CO-*syn* conformer **5a-s** is both kinetically (ΔG_{att} = 11.0 *versus* 12.1 kcal mol^{-1} at -20°C) and thermodynamically (ΔG_{att} = -1.9 *versus* -0.7 kcal mol^{-1}) preferred. The reaction terminates after protonation of the adduct **8**:

$$\mathbf{8a} + \text{MeOH} \longrightarrow \mathbf{6a} + \text{MeONa}$$

Scheme S-1. The mechanism of the uncatalyzed reaction.

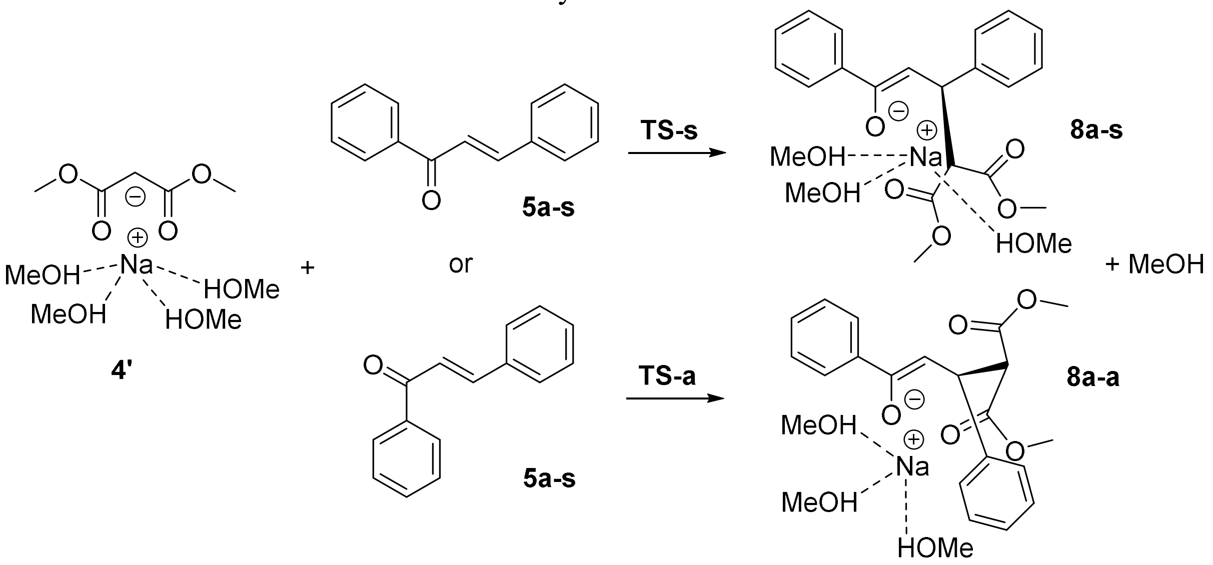

Table S-1. Absolute and relative energies for the uncatalyzed reaction.

**No Catalyzer - Calcone**		E DZ(D) /au	*ΔE*	ZPE/au	ΔE^{0K}	Δn	δG^{298K} /au	δG^{273K} /au	δG^{253K} /au	ΔG^{298K}	ΔG^{273K}	ΔG^{253K}
Malonate-Na^+ $(MeOH)_4$	**4'**	-1119.845074		0.336717			0.274232	0.281870	0.287739	**Conc. MeOH:**		**24.72**
Calcone CO-*syns*	**5a-s**	-653.303691		0.226788			0.184329	0.188973	0.192562			
Calcone CO-*anti*	**5a-a**	-653.302195	**0.94**	0.226791	**0.94**		0.185521					
Methanol		-115.591709		0.051595			0.028818	0.031069	0.032843			
**4 + 5a(CO-*syns*)**		-1773.148765	**0.00**	0.563505	**0.00**	**0**	0.458561	0.470843	0.480301	**0.00**	**0.00**	**0.00**
TS_{Add} 1,4 $(MeOH)_3$ CO-*syn*	**TS-s**	-1657.545862		0.511507			0.437387	0.446695	0.453808	**1.3E+04**	**4.6E+03**	**1.7E+03**
**TS_{Add} + MeOH**		-1773.137571	**7.02**	0.563102	**6.77**	**0**	0.466205	0.477764	0.486651	**11.82**	**11.37**	**11.01**
Cpl Adduct CO-*syn*	**8a-s**	-1657.568380		0.514154			0.439279	0.448612	0.455748			
**4 + MeOH**		-1773.160089	**-7.11**	0.565749	**-5.70**	**0**	0.468097	0.479681	0.488591	**-1.12**	**-1.56**	**-1.90**
TS_{Add} 1,4 $(MeOH)_3$ CO-*anti*	**TS-a**	-1657.545650		0.512296			0.439156	0.448329	0.455337	**1.7E+03**	**5.4E+02**	**1.9E+02**
**TS_{Add} + MeOH**		-1773.137359	**7.16**	0.563891	**7.40**	**0**	0.467974	0.479398	0.488180	**13.06**	**12.53**	**12.10**
Cpl Adduct CO-*anti*	**8a-a**	-1657.568998		0.515057			0.442143	0.451263	0.458232			
**4a + MeOH**		-1773.160707	**-7.49**	0.566652	**-5.52**	**0**	0.470961	0.482332	0.491075	**0.29**	**-0.28**	**-0.73**

All energies are in kcal mol^{-1}. ΔE^{0K} include ZPE, ΔG are Gibbs free energies.

Pictures and Cartesian coordinates.

4’

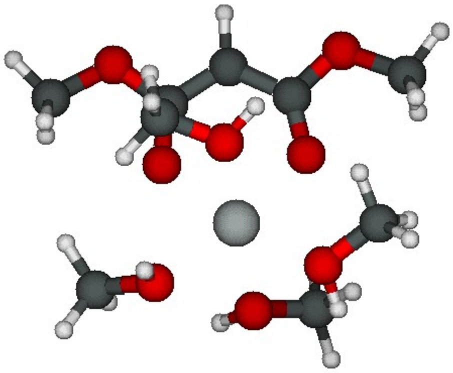

1	6	0	0.012972	0.465832	-0.121901
2	1	0	0.030437	0.986938	0.832011
3	6	0	1.243059	0.097436	-0.711299
4	6	0	-1.239208	0.155429	-0.698550
5	8	0	2.317297	0.491129	0.035286
6	8	0	1.444229	-0.505752	-1.775448
7	6	0	3.603389	0.212125	-0.498291
8	1	0	4.326907	0.584651	0.235415
9	1	0	3.748669	-0.867154	-0.643066
10	1	0	3.753823	0.729860	-1.456278
11	8	0	-2.287313	0.578192	0.068913
12	8	0	-1.475919	-0.426732	-1.766778
13	6	0	-3.591474	0.338214	-0.440631
14	1	0	-4.289499	0.721010	0.312224
15	1	0	-3.748017	0.869436	-1.389689
16	1	0	-3.766890	-0.735538	-0.592734
17	11	0	-0.017029	-0.246715	-3.509114
18	8	0	-0.267132	-2.469565	-4.109806
19	6	0	-1.532941	-2.999503	-3.747356
20	1	0	-1.786197	-3.880798	-4.356300
21	1	0	-1.565217	-3.275006	-2.682848
22	1	0	-2.283941	-2.219647	-3.928548
23	1	0	0.416827	-3.114977	-3.898428
24	8	0	0.141564	2.049720	-3.165603
25	6	0	1.425405	2.549489	-2.828624
26	1	0	1.564242	3.577416	-3.198346
27	1	0	1.590794	2.538479	-1.739811
28	1	0	2.171549	1.899710	-3.304638
29	1	0	-0.524556	2.507454	-2.639909
30	8	0	1.681723	-0.040227	-5.056568
31	6	0	2.896567	-0.675515	-4.692726
32	1	0	3.433310	-0.109446	-3.915692
33	1	0	2.645732	-1.665999	-4.291403
34	1	0	3.556346	-0.806894	-5.563879
35	1	0	1.872281	0.844995	-5.387976
36	8	0	-1.726785	0.179064	-4.996547
37	6	0	-2.882671	0.788826	-4.445873
38	1	0	-3.497057	0.063229	-3.890984
39	1	0	-2.544953	1.569259	-3.751432
40	1	0	-3.498577	1.258608	-5.228097
41	1	0	-1.993351	-0.537823	-5.583733

5a-s

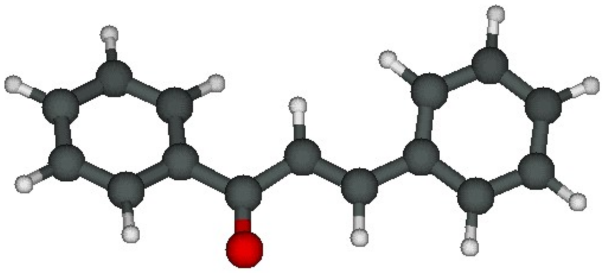

1	6	0	0.090008	-0.158565	-0.080886
2	6	0	0.170914	-0.127985	1.262319
3	1	0	0.983749	-0.118218	-0.703073
4	6	0	1.392796	-0.037756	2.067650
5	1	0	-0.764324	-0.179908	1.829732
6	6	0	-1.230606	-0.231125	-0.744456
7	8	0	-2.272876	-0.127735	-0.108345
8	6	0	-1.282339	-0.421448	-2.228645
9	6	0	2.673320	0.091401	1.499928
10	6	0	3.797959	0.171819	2.313691
11	6	0	3.666755	0.125023	3.705274
12	6	0	2.402470	-0.000750	4.280166
13	6	0	1.274192	-0.080247	3.466280
14	1	0	2.791199	0.133427	0.415587
15	1	0	4.786374	0.273266	1.861952
16	1	0	4.553169	0.188046	4.339327
17	1	0	2.294009	-0.036271	5.365618
18	1	0	0.281806	-0.178026	3.912455
19	6	0	-2.485525	-0.138691	-2.892363
20	6	0	-2.587756	-0.314259	-4.268392
21	6	0	-1.491954	-0.788588	-4.994292
22	6	0	-0.295299	-1.081670	-4.340492
23	6	0	-0.185606	-0.892889	-2.963371
24	1	0	-3.336398	0.228848	-2.316496
25	1	0	-3.523755	-0.081945	-4.779290
26	1	0	-1.572728	-0.930944	-6.073685
27	1	0	0.559135	-1.459473	-4.904406
28	1	0	0.754339	-1.136685	-2.467689

5a-a

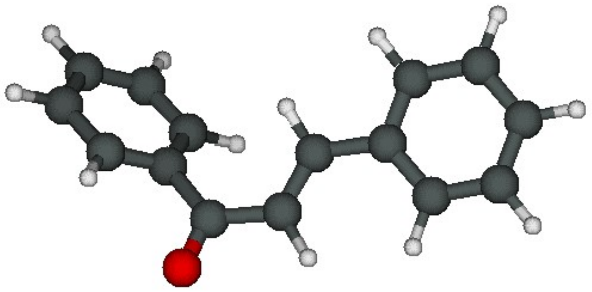

1	6	0	-0.056342	0.168531	0.017013
2	6	0	0.169827	-0.257893	1.274321
3	1	0	0.733361	0.625438	-0.585076
4	6	0	1.433354	-0.145706	2.014178
5	1	0	-0.636701	-0.761433	1.816161
6	6	0	-1.318555	-0.048196	-0.712638

7	8	0	-1.318487	-0.019364	-1.938634
8	6	0	-2.598040	-0.315779	0.017307
9	6	0	2.572452	0.488357	1.486860
10	6	0	3.742314	0.567280	2.234484
11	6	0	3.797663	0.016378	3.518569
12	6	0	2.674223	-0.613861	4.052825
13	6	0	1.500801	-0.693351	3.305283
14	1	0	2.544921	0.924723	0.486866
15	1	0	4.619807	1.062076	1.814372
16	1	0	4.718709	0.080692	4.101011
17	1	0	2.710954	-1.044543	5.055033
18	1	0	0.618654	-1.185710	3.721163
19	6	0	-3.509795	-1.215149	-0.552368
20	6	0	-4.729691	-1.463263	0.070460
21	6	0	-5.058105	-0.794900	1.252373
22	6	0	-4.163551	0.118019	1.811635
23	6	0	-2.931282	0.353555	1.202651
24	1	0	-3.246594	-1.725954	-1.480825
25	1	0	-5.429286	-2.177287	-0.367561
26	1	0	-6.018094	-0.983766	1.736762
27	1	0	-4.426152	0.651924	2.726567
28	1	0	-2.239665	1.077835	1.636610

TS-s (ω = i 449 cm^{-1})

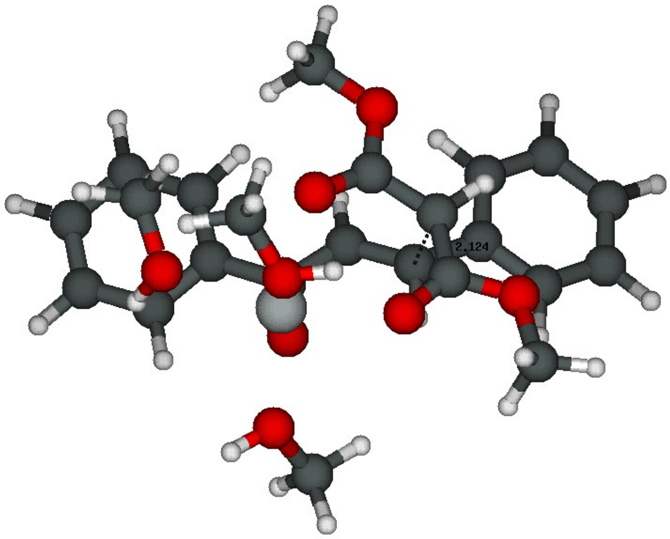

1	6	0	-0.260961	-0.043380	0.045998
2	1	0	-0.325121	0.066618	1.126379
3	6	0	1.077698	-0.086372	-0.493058
4	6	0	-1.340635	0.566293	-0.701857
5	8	0	1.986061	-0.380332	0.450798
6	8	0	1.408640	0.017630	-1.665839
7	6	0	3.330991	-0.548973	0.006345
8	1	0	3.914938	-0.808549	0.895174
9	1	0	3.395984	-1.355946	-0.735905
10	1	0	3.710497	0.383467	-0.432863
11	8	0	-2.391317	0.838899	0.089430
12	8	0	-1.393364	0.768786	-1.909261
13	6	0	-3.571323	1.320571	-0.549352
14	1	0	-4.310883	1.461232	0.245381
15	1	0	-3.375750	2.276304	-1.052763

16	1	0	-3.940237	0.588319	-1.279703
17	11	0	0.043578	-0.084037	-3.499067
18	8	0	-0.447473	-2.261396	-2.951479
19	6	0	0.378261	-2.833068	-2.188659
20	6	0	0.277449	-2.812279	-0.779825
21	6	0	1.525133	-3.569730	-2.835978
22	1	0	1.022489	-3.322297	-0.172183
23	6	0	-0.766930	-2.096335	-0.157010
24	6	0	1.453288	-3.849532	-4.208433
25	6	0	2.519742	-4.454202	-4.872096
26	6	0	3.679370	-4.790696	-4.170498
27	6	0	3.756548	-4.531750	-2.801292
28	6	0	2.688204	-3.928292	-2.137935
29	1	0	0.547314	-3.580712	-4.755263
30	1	0	2.446106	-4.663354	-5.941131
31	1	0	4.518777	-5.258299	-4.688661
32	1	0	4.658614	-4.793051	-2.244829
33	1	0	2.782080	-3.716857	-1.072385
34	6	0	-1.145775	-2.354693	1.255628
35	1	0	-1.616640	-1.866761	-0.806869
36	6	0	-2.482754	-2.192964	1.646485
37	6	0	-2.872438	-2.427595	2.965210
38	6	0	-1.927846	-2.819047	3.914127
39	6	0	-0.592258	-2.977435	3.535477
40	6	0	-0.202817	-2.748872	2.217616
41	1	0	-3.222522	-1.883934	0.903687
42	1	0	-3.918648	-2.304015	3.251578
43	1	0	-2.229977	-3.001432	4.947133
44	1	0	0.152268	-3.281610	4.273730
45	1	0	0.845947	-2.867448	1.937157
46	8	0	-1.682631	-0.117858	-5.015435
47	6	0	-2.790832	-0.931352	-4.663113
48	1	0	-3.717644	-0.565393	-5.130055
49	1	0	-2.629744	-1.980660	-4.952672
50	1	0	-2.903857	-0.882317	-3.572834
51	1	0	-1.582398	-0.115077	-5.973878
52	8	0	1.909004	-0.683072	-4.762014
53	6	0	3.222473	-0.775330	-4.232841
54	1	0	3.967963	-0.883881	-5.035668
55	1	0	3.429248	0.153045	-3.686016
56	1	0	3.317641	-1.620948	-3.533566
57	1	0	1.717372	-1.494387	-5.248444
58	8	0	0.470625	2.165280	-3.928016
59	6	0	1.823076	2.591964	-3.938690
60	1	0	1.897609	3.683342	-4.061121
61	1	0	2.347134	2.299133	-3.015459
62	1	0	2.313939	2.111078	-4.794429
63	1	0	0.011940	2.575880	-3.184186

8a-s

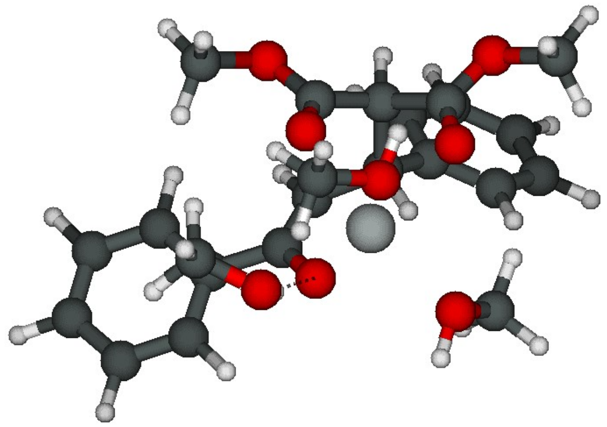

1	6	0	-0.264517	-0.023552	0.363884
2	1	0	-0.344349	-0.156424	1.447681
3	6	0	1.202891	0.058894	0.003349
4	6	0	-1.022033	1.210218	-0.063569
5	8	0	1.964843	-0.483075	0.937141
6	8	0	1.647958	0.530098	-1.020217
7	6	0	3.359726	-0.596578	0.634413
8	1	0	3.814553	-1.105120	1.489432
9	1	0	3.495019	-1.188260	-0.280733
10	1	0	3.800316	0.399727	0.502952
11	8	0	-1.772353	1.711379	0.899232
12	8	0	-0.998801	1.657497	-1.192131
13	6	0	-2.609239	2.821563	0.551855
14	1	0	-3.158773	3.080439	1.461275
15	1	0	-1.993139	3.667095	0.222215
16	1	0	-3.303693	2.529571	-0.245998
17	11	0	0.350725	0.737335	-2.922906
18	8	0	0.375532	-1.595372	-2.897195
19	6	0	0.678273	-2.396318	-1.909880
20	6	0	0.080893	-2.345344	-0.680457
21	6	0	1.747690	-3.417558	-2.190177
22	1	0	0.355000	-3.054249	0.101405
23	6	0	-0.918399	-1.265184	-0.379872
24	6	0	1.999600	-3.798921	-3.516122
25	6	0	2.995147	-4.729172	-3.816793
26	6	0	3.768307	-5.284017	-2.796534
27	6	0	3.536098	-4.901966	-1.473378
28	6	0	2.536921	-3.977463	-1.172975
29	1	0	1.400838	-3.361429	-4.317040
30	1	0	3.169454	-5.018943	-4.855058
31	1	0	4.552691	-6.006375	-3.030547
32	1	0	4.144411	-5.320087	-0.668763
33	1	0	2.382573	-3.672753	-0.135771
34	6	0	-2.135170	-1.714710	0.404857
35	1	0	-1.270169	-0.883608	-1.348534
36	6	0	-3.421199	-1.416154	-0.058100
37	6	0	-4.549489	-1.803877	0.669398
38	6	0	-4.402811	-2.494854	1.871022
39	6	0	-3.122328	-2.794598	2.343374
40	6	0	-1.998421	-2.405627	1.617012
41	1	0	-3.538745	-0.876968	-1.001869
42	1	0	-5.545851	-1.565025	0.292214
43	1	0	-5.282845	-2.799550	2.440639

44	1	0	-2.999209	-3.333853	3.284778
45	1	0	-1.000194	-2.637627	1.998024
46	8	0	-1.490371	0.715185	-4.285778
47	6	0	-2.628466	0.098011	-3.700931
48	1	0	-3.527062	0.256286	-4.315703
49	1	0	-2.471925	-0.982720	-3.559961
50	1	0	-2.787167	0.569154	-2.722455
51	1	0	-1.359749	0.363393	-5.174387
52	8	0	2.166560	-0.339786	-4.209532
53	6	0	3.480663	-0.546621	-3.738456
54	1	0	3.901462	-1.498514	-4.106300
55	1	0	4.121848	0.269018	-4.102937
56	1	0	3.525944	-0.550463	-2.635579
57	1	0	1.554674	-0.998222	-3.762215
58	8	0	1.210092	2.863335	-3.124624
59	6	0	2.597859	2.901475	-3.424298
60	1	0	2.975961	3.934609	-3.428673
61	1	0	3.181307	2.307216	-2.703740
62	1	0	2.730922	2.476143	-4.426803
63	1	0	1.055423	3.312441	-2.284363

TS-a (ω = i 407 cm^{-1})

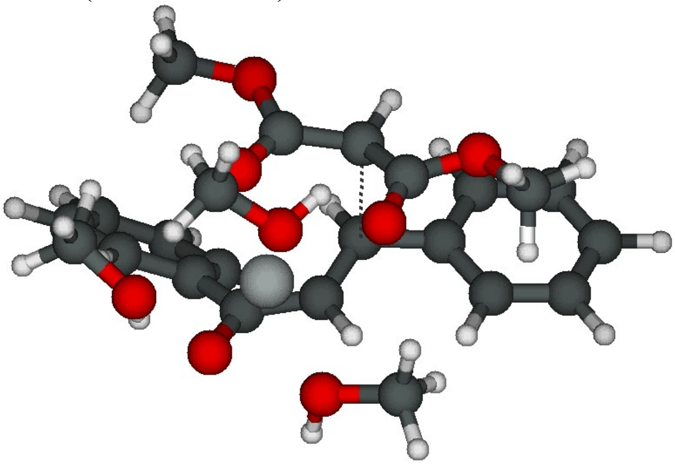

1	6	0	0.083738	-0.013619	-0.049289
2	1	0	0.136555	-0.037430	1.038147
3	6	0	1.355240	0.004929	-0.732526
4	6	0	-1.044090	0.690924	-0.611533
5	8	0	2.379920	-0.255329	0.097424
6	8	0	1.538448	0.166955	-1.934115
7	6	0	3.689618	-0.229747	-0.464795
8	1	0	4.379356	-0.420933	0.363641
9	1	0	3.799906	-1.008133	-1.230318
10	1	0	3.897406	0.754596	-0.905562
11	8	0	-1.936811	1.062145	0.320238
12	8	0	-1.255561	0.890943	-1.803383
13	6	0	-3.139856	1.665458	-0.149091
14	1	0	-3.761228	1.834896	0.736313
15	1	0	-2.921667	2.621861	-0.643414
16	1	0	-3.661008	1.001653	-0.852005
17	11	0	-0.030464	0.117232	-3.611275
18	8	0	0.482360	-2.290429	-3.999826
19	6	0	0.504990	-2.519869	-2.755831
20	6	0	-0.576244	-2.145784	-1.922352

21	6	0	1.707126	-3.243448	-2.213432
22	1	0	-1.470647	-1.818534	-2.460571
23	6	0	-0.527533	-2.000306	-0.523482
24	6	0	2.964968	-2.981952	-2.773704
25	6	0	4.095097	-3.669289	-2.335022
26	6	0	3.978544	-4.642174	-1.340289
27	6	0	2.726626	-4.928976	-0.794615
28	6	0	1.596661	-4.235728	-1.228481
29	1	0	3.055261	-2.225672	-3.555367
30	1	0	5.070312	-3.445014	-2.771375
31	1	0	4.861999	-5.185322	-0.999260
32	1	0	2.625702	-5.704859	-0.033462
33	1	0	0.616940	-4.487248	-0.817930
34	6	0	-1.761056	-2.022029	0.308858
35	1	0	0.347637	-2.395435	-0.003527
36	6	0	-3.038053	-1.831206	-0.237776
37	6	0	-4.165131	-1.815564	0.584236
38	6	0	-4.035678	-1.999116	1.961287
39	6	0	-2.769363	-2.205050	2.513978
40	6	0	-1.643738	-2.212794	1.693995
41	1	0	-3.162055	-1.685179	-1.312223
42	1	0	-5.151766	-1.660768	0.143002
43	1	0	-4.919276	-1.988123	2.602217
44	1	0	-2.658884	-2.358175	3.589191
45	1	0	-0.651561	-2.360340	2.128613
46	8	0	-1.960298	-0.309289	-4.770579
47	6	0	-3.186580	0.159470	-4.232514
48	1	0	-4.006856	0.052255	-4.958185
49	1	0	-3.451593	-0.377137	-3.307875
50	1	0	-3.060958	1.224056	-3.998399
51	1	0	-2.047104	-1.243259	-4.996335
52	8	0	1.767567	-0.142881	-5.130980
53	6	0	3.137036	0.195129	-5.003252
54	1	0	3.777853	-0.526187	-5.533802
55	1	0	3.287601	1.184468	-5.454366
56	1	0	3.449521	0.242023	-3.947030
57	1	0	1.633732	-1.078614	-4.900837
58	8	0	-0.124086	2.411077	-3.974918
59	6	0	1.139219	2.975194	-4.288606
60	1	0	1.130768	4.069090	-4.167804
61	1	0	1.937948	2.550698	-3.659879
62	1	0	1.352406	2.739974	-5.338881
63	1	0	-0.362158	2.631344	-3.064580

8a-a

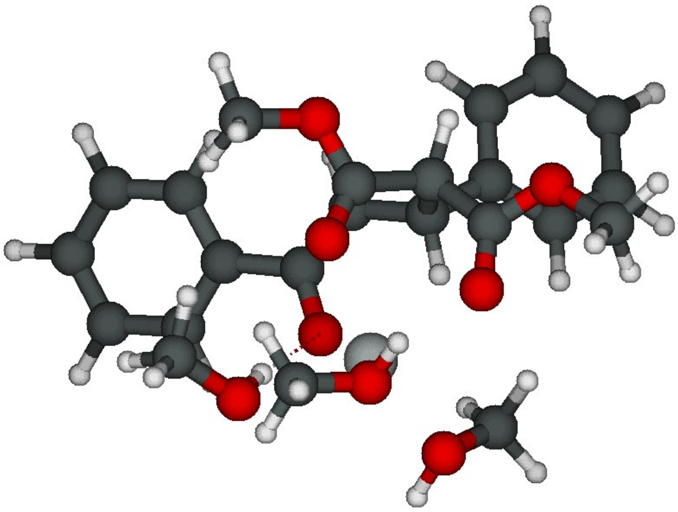

1	6	0	-0.264517	-0.023552	0.363884
2	1	0	-0.344349	-0.156424	1.447681
3	6	0	1.202891	0.058894	0.003349
4	6	0	-1.022033	1.210218	-0.063569
5	8	0	1.964843	-0.483075	0.937141
6	8	0	1.647958	0.530098	-1.020217
7	6	0	3.359726	-0.596578	0.634413
8	1	0	3.814553	-1.105120	1.489432
9	1	0	3.495019	-1.188260	-0.280733
10	1	0	3.800316	0.399727	0.502952
11	8	0	-1.772353	1.711379	0.899232
12	8	0	-0.998801	1.657497	-1.192131
13	6	0	-2.609239	2.821563	0.551855
14	1	0	-3.158773	3.080439	1.461275
15	1	0	-1.993139	3.667095	0.222215
16	1	0	-3.303693	2.529571	-0.245998
17	11	0	0.350725	0.737335	-2.922906
18	8	0	0.375532	-1.595372	-2.897195
19	6	0	0.678273	-2.396318	-1.909880
20	6	0	0.080893	-2.345344	-0.680457
21	6	0	1.747690	-3.417558	-2.190177
22	1	0	0.355000	-3.054249	0.101405
23	6	0	-0.918399	-1.265184	-0.379872
24	6	0	1.999600	-3.798921	-3.516122
25	6	0	2.995147	-4.729172	-3.816793
26	6	0	3.768307	-5.284017	-2.796534
27	6	0	3.536098	-4.901966	-1.473378
28	6	0	2.536921	-3.977463	-1.172975
29	1	0	1.400838	-3.361429	-4.317040
30	1	0	3.169454	-5.018943	-4.855058
31	1	0	4.552691	-6.006375	-3.030547
32	1	0	4.144411	-5.320087	-0.668763
33	1	0	2.382573	-3.672753	-0.135771
34	6	0	-2.135170	-1.714710	0.404857
35	1	0	-1.270169	-0.883608	-1.348534
36	6	0	-3.421199	-1.416154	-0.058100
37	6	0	-4.549489	-1.803877	0.669398
38	6	0	-4.402811	-2.494854	1.871022
39	6	0	-3.122328	-2.794598	2.343374
40	6	0	-1.998421	-2.405627	1.617012

41	1	0	-3.538745	-0.876968	-1.001869
42	1	0	-5.545851	-1.565025	0.292214
43	1	0	-5.282845	-2.799550	2.440639
44	1	0	-2.999209	-3.333853	3.284778
45	1	0	-1.000194	-2.637627	1.998024
46	8	0	-1.490371	0.715185	-4.285778
47	6	0	-2.628466	0.098011	-3.700931
48	1	0	-3.527062	0.256286	-4.315703
49	1	0	-2.471925	-0.982720	-3.559961
50	1	0	-2.787167	0.569154	-2.722455
51	1	0	-1.359749	0.363393	-5.174387
52	8	0	2.166560	-0.339786	-4.209532
53	6	0	3.480663	-0.546621	-3.738456
54	1	0	3.901462	-1.498514	-4.106300
55	1	0	4.121848	0.269018	-4.102937
56	1	0	3.525944	-0.550463	-2.635579
57	1	0	1.554674	-0.998222	-3.762215
58	8	0	1.210092	2.863335	-3.124624
59	6	0	2.597859	2.901475	-3.424298
60	1	0	2.975961	3.934609	-3.428673
61	1	0	3.181307	2.307216	-2.703740
62	1	0	2.730922	2.476143	-4.426803
63	1	0	1.055423	3.312441	-2.284363

Schemes, table, pictures and Cartesian coordinates. The catalyzed reaction.

In the computational study (Scheme S-2 and Table S-2) we start with the complex between the catalyzer and the sodium malonate with one explicit molecule of methanol, **9**. This one bound with a molecule of calcone in its (CO-*syn* **5a-s** conformation (choice based on the uncatalyzed reaction) releasing the last molecule of methanol. Two transition structures can follow: **TS-R** that yields the (R)-adduct **10a-R** and **TS-S** that yields the (S)-adduct **10a-S**. The reaction leading to the (R) adduct is slightly kinetically favored: ΔG_{att} = 11.6 *versus* 12.0 kcal mol^{-1} at -20°C. The calculated rate constants are, respectively, 1.0 • 10^4 *versus* 4.3 • 10^3 that lead to an ee of 37% in favor of the product **(R)-6a**. The reaction terminates after protonation of the adduct **10**:

10a + MeOH ⟶ **6a** + MeONa

Scheme S-2. The mechanism of the catalyzed reaction.

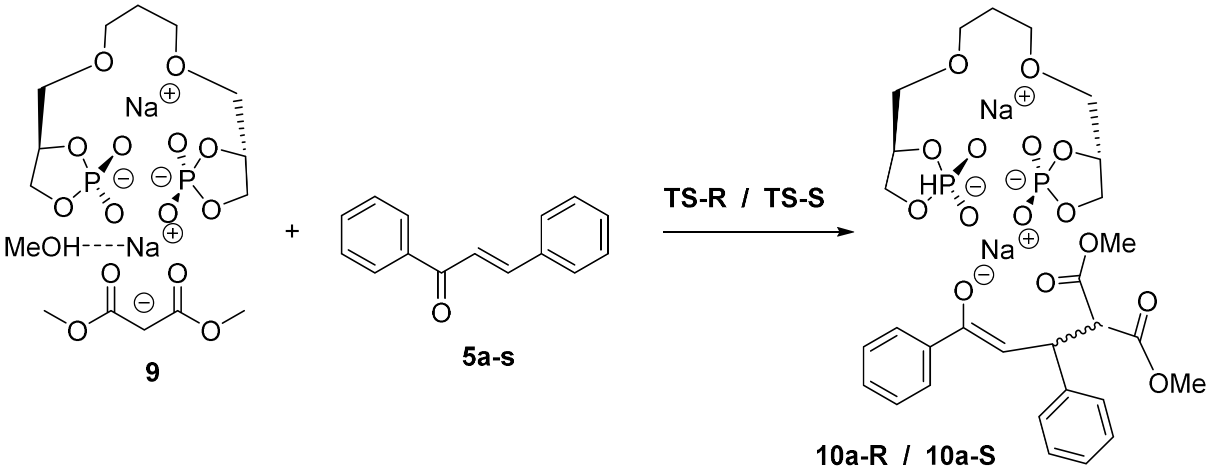

Table S-2. Absolute and relative energies for the catalyzed reaction.

**Catalyzed - Calcone**		E DZ(D) /au	ΔE	ZPE/au	ΔE^{0K}	Δn	δG^{298K} /au	δG^{273K} /au	δG^{253K} /au	ΔG^{298K}	ΔG^{273K}	ΔG^{253K}
$[Cat^{--} Na^{+} Na^{+} Mal^{-}]^{-}$ MeOH	**9**	-2721.870481		0.464746	**0.93**		0.391371	0.400630	0.407692	**0.56**	**0.59**	**0.61**
Calcone *s-cis*	**5a-s**	-653.303691		0.226788			0.184329	0.188973	0.192562			
**9 + 2a-s**		-3375.174172	**0.00**	0.691534	**0.00**	**0**	0.575700	0.589603	0.600254	**0.00**	**0.00**	**0.00**
Methanol		-115.591709		0.051595			0.028818	0.031069	0.032843			
TS_{Add} (1,4) **R**	**TS-R**	-3259.569582		0.639335			0.553734	0.564706	0.573047	**1.3E+05**	**3.5E+04**	**1.0E+04**
**TS_{Add} + MeOH**		-3375.161291	**8.08**	0.690930	**7.70**	**0**	0.582552	0.595775	0.605890	**12.38**	**11.96**	**11.62**
Add. **R** 9 + Calcone CO-*syn*	**10a-R**	-3259.586471		0.641843			0.554175	0.565330	0.573816			
**10a-R + MeOH**		-3375.178180	**-2.52**	0.693438	**-1.32**	**0**	0.582993	0.596399	0.606659	**2.06**	**1.75**	**1.50**
TS_{Add} (1,4) **S**	**TS-S**	-3259.569044		0.639620			0.553871	0.564854	0.573203	**6.2E+04**	**1.6E+04**	**4.3E+03**
**TS_{Add} + MeOH**		-3375.160753	**8.42**	0.691215	**8.22**	**0**	0.582689	0.595923	0.606046	**12.81**	**12.39**	**12.05**
Add. **S** 5 + Calcone CO-*syn*	**10a-S**	-3259.588891		0.641881			0.553889	0.565079	0.573593			
**10a-S + MeOH**		-3375.180600	**-4.03**	0.693476	**-2.82**	**0**	0.582707	0.596148	0.606436	**0.36**	**0.07**	**-0.15**

All energies are in kcal mol^{-1}. ΔE^{0K} include ZPE, ΔG are Gibbs free energies.

Pictures and Cartesian coordinates.

9

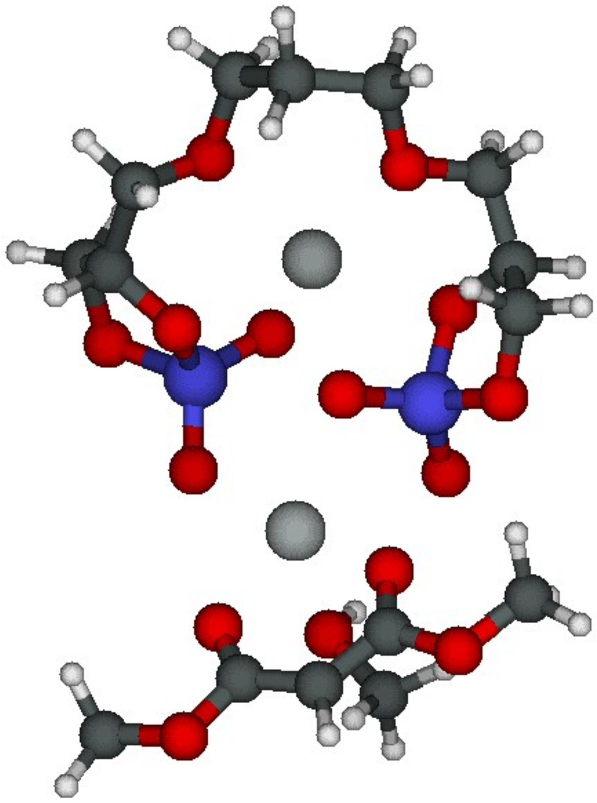

1	8	0	0.414111	0.399803	0.165080
2	15	0	0.332328	0.298249	1.662485
3	8	0	1.596354	0.119747	2.476252
4	8	0	-0.728391	-0.883868	2.138456
5	8	0	-0.578034	1.520770	2.268075
6	6	0	-1.728820	-0.393430	3.040084
7	6	0	-1.300462	1.038642	3.403901
8	1	0	-2.691996	-0.378531	2.510806
9	6	0	-1.840500	-1.329070	4.223863
10	1	0	-2.165724	1.689024	3.570779
11	1	0	-0.649215	1.048029	4.290512
12	11	0	1.235589	-1.410012	-1.004020
13	11	0	1.077086	-1.996181	3.295617
14	8	0	1.316015	-2.647207	1.038545
15	15	0	2.822140	-2.831015	1.063396
16	8	0	3.656832	-1.992294	0.141865
17	8	0	3.280853	-2.684335	2.651817
18	8	0	3.232372	-4.412313	0.960229
19	6	0	3.637530	-3.946468	3.234875
20	6	0	3.124999	-5.010028	2.254486
21	1	0	4.733140	-3.997101	3.309128
22	6	0	3.052777	-4.057138	4.625738
23	1	0	2.075203	-5.269399	2.459558
24	1	0	3.743822	-5.913478	2.276416
25	8	0	-0.569358	-1.473105	4.827796
26	1	0	-2.560359	-0.904506	4.944873
27	1	0	-2.222429	-2.305394	3.883434
28	8	0	1.665139	-3.782497	4.593033
29	1	0	3.234698	-5.082403	4.992339

30	1	0	3.557816	-3.350300	5.304378
31	6	0	-0.584288	-2.241086	6.028324
32	6	0	-0.438915	-3.743485	5.795174
33	1	0	0.248260	-1.870887	6.645500
34	1	0	-1.521754	-2.029491	6.565978
35	6	0	0.989844	-4.247445	5.754952
36	1	0	1.532656	-3.899890	6.651994
37	1	0	1.003662	-5.350572	5.753853
38	1	0	-0.933016	-4.270516	6.624571
39	1	0	-0.971733	-4.035455	4.875547
40	6	0	0.518502	-3.491811	-3.906190
41	1	0	0.361914	-4.069380	-4.813684
42	6	0	1.479773	-3.937836	-2.971832
43	8	0	2.129700	-5.067560	-3.382272
44	8	0	1.782271	-3.439276	-1.880349
45	6	0	3.095962	-5.602520	-2.488379
46	1	0	3.537603	-6.466712	-2.996998
47	1	0	3.879607	-4.866142	-2.262993
48	1	0	2.625463	-5.928025	-1.550527
49	6	0	-0.251416	-2.325931	-3.702339
50	8	0	-1.130292	-2.092570	-4.721830
51	8	0	-0.212319	-1.528643	-2.753603
52	6	0	-1.939548	-0.930377	-4.613228
53	1	0	-2.569590	-0.911026	-5.509508
54	1	0	-2.574450	-0.972412	-3.717669
55	1	0	-1.323365	-0.021679	-4.576710
56	8	0	2.674945	-0.140322	-2.306533
57	6	0	3.236062	-0.925685	-3.346694
58	1	0	3.810205	-0.304488	-4.051104
59	1	0	3.890289	-1.716661	-2.948259
60	1	0	2.405612	-1.396662	-3.889126
61	1	0	3.385942	0.271909	-1.801849

TS-R (ω = i 445 cm^{-1})

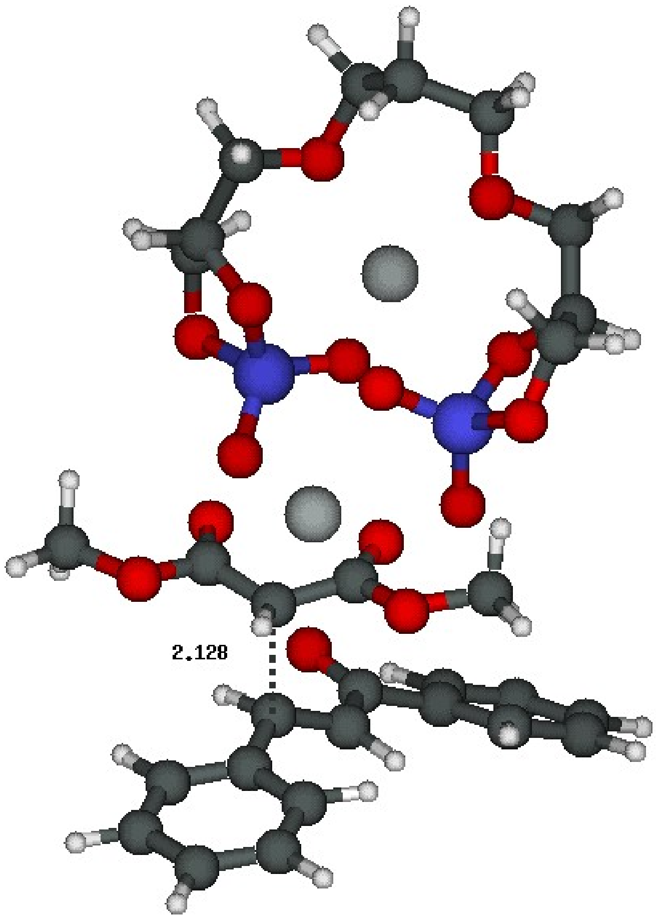

1	8	0	-0.012695	-0.031902	-0.012052
2	15	0	-0.023023	-0.024723	1.490942
3	8	0	1.283962	-0.014043	2.254227
4	8	0	-0.936779	-1.265968	2.103456
5	8	0	-1.028270	1.144172	2.050851
6	6	0	-1.934527	-0.808714	3.025420
7	6	0	-1.638995	0.683116	3.258762
8	1	0	-2.919894	-0.930432	2.553885
9	6	0	-1.890410	-1.656627	4.278210
10	1	0	-2.557425	1.255408	3.427531
11	1	0	-0.948612	0.827245	4.103083
12	11	0	1.068950	-1.753468	-1.082567
13	11	0	1.023786	-2.097125	3.244882
14	8	0	1.252562	-2.889840	1.049533
15	15	0	2.768843	-2.859677	1.005871
16	8	0	3.421288	-1.970838	-0.012400
17	8	0	3.279354	-2.539525	2.551423
18	8	0	3.397935	-4.370180	0.976673
19	6	0	3.820970	-3.700784	3.197650
20	6	0	3.423167	-4.887571	2.309555
21	1	0	4.915243	-3.601220	3.227887
22	6	0	3.303936	-3.791118	4.616712
23	1	0	2.427424	-5.271431	2.579165
24	1	0	4.160458	-5.696006	2.358520
25	8	0	-0.583456	-1.627005	4.819515
26	1	0	-2.614735	-1.251951	5.006255
27	1	0	-2.184767	-2.689505	4.029911
28	8	0	1.892670	-3.692473	4.627195

29	1	0	3.626636	-4.758983	5.038097
30	1	0	3.739080	-2.983333	5.227742
31	6	0	-0.455935	-2.304104	6.067244
32	6	0	-0.150627	-3.794660	5.927044
33	1	0	0.355864	-1.800279	6.613401
34	1	0	-1.385960	-2.159943	6.638706
35	6	0	1.323033	-4.141538	5.850308
36	1	0	1.859915	-3.671133	6.693201
37	1	0	1.460925	-5.233520	5.924863
38	1	0	-0.547427	-4.312229	6.812788
39	1	0	-0.685315	-4.208033	5.056285
40	6	0	0.282813	-4.172046	-3.599957
41	1	0	-0.003675	-4.957862	-4.295565
42	6	0	1.426296	-4.463280	-2.768668
43	8	0	2.159226	-5.475788	-3.262391
44	8	0	1.800302	-3.843193	-1.784771
45	6	0	3.353023	-5.798922	-2.551202
46	1	0	3.858779	-6.570590	-3.140408
47	1	0	3.998035	-4.916266	-2.448868
48	1	0	3.108175	-6.188346	-1.553896
49	6	0	-0.809646	-3.370560	-3.089700
50	8	0	-1.912238	-3.505098	-3.847030
51	8	0	-0.790406	-2.593469	-2.143151
52	6	0	-3.023442	-2.676012	-3.514914
53	1	0	-3.805277	-2.908730	-4.244999
54	1	0	-3.380104	-2.896417	-2.500371
55	1	0	-2.747159	-1.615765	-3.585011
56	8	0	1.906789	-0.868933	-3.010882
57	6	0	2.802741	-1.550872	-3.579634
58	6	0	2.527624	-2.528076	-4.563165
59	6	0	1.194883	-2.793564	-4.940532
60	1	0	3.344001	-3.092836	-5.010141
61	6	0	0.872851	-3.502505	-6.205102
62	1	0	0.480428	-2.005994	-4.684218
63	6	0	-0.316165	-3.189570	-6.879531
64	6	0	-0.652921	-3.838241	-8.067617
65	6	0	0.191280	-4.816485	-8.593475
66	6	0	1.375898	-5.138447	-7.926254
67	6	0	1.716319	-4.486922	-6.742646
68	1	0	-0.978783	-2.425148	-6.465884
69	1	0	-1.579058	-3.578017	-8.583859
70	1	0	-0.071239	-5.327478	-9.521763
71	1	0	2.040062	-5.904289	-8.331566
72	1	0	2.640847	-4.753787	-6.226261
73	6	0	4.233695	-1.284876	-3.186847
74	6	0	5.260678	-2.215640	-3.401106
75	6	0	6.560600	-1.947710	-2.972660
76	6	0	6.856265	-0.742050	-2.334676
77	6	0	5.841728	0.193498	-2.121899
78	6	0	4.540223	-0.080463	-2.537847
79	1	0	5.047843	-3.169522	-3.885253
80	1	0	7.346142	-2.688153	-3.135095
81	1	0	7.874704	-0.533395	-2.001284
82	1	0	6.064422	1.138855	-1.623411
83	1	0	3.743389	0.642807	-2.358363

10a-R

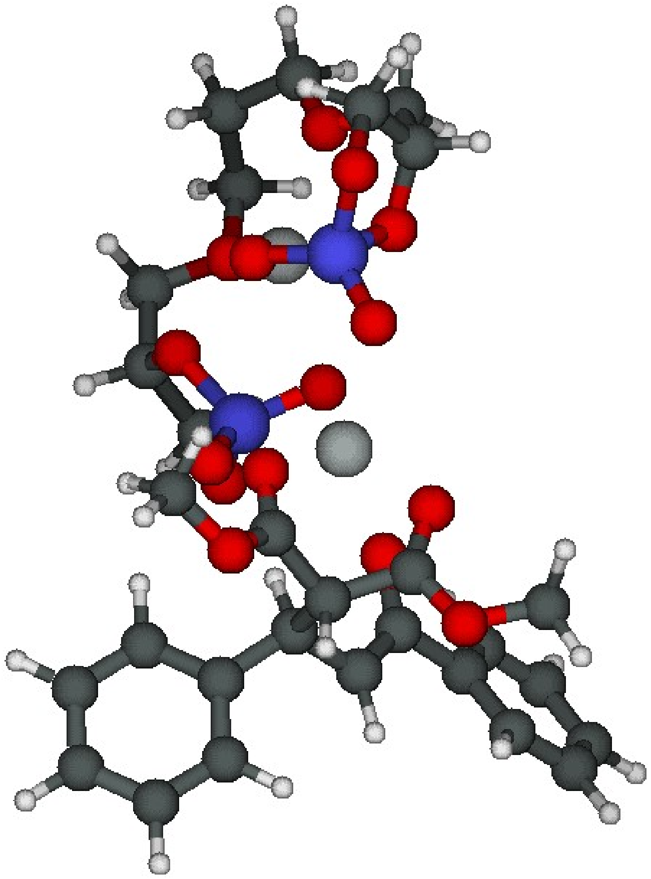

1	8	0	0.898026	0.712352	-0.115303
2	15	0	0.866067	0.442504	1.362518
3	8	0	2.073999	-0.237874	1.977620
4	8	0	-0.466846	-0.426816	1.820740
5	8	0	0.432534	1.782204	2.195986
6	6	0	-1.257708	0.251114	2.807044
7	6	0	-0.383070	1.409729	3.310359
8	1	0	-2.162027	0.640819	2.318783
9	6	0	-1.669210	-0.731237	3.880905
10	1	0	-0.986768	2.274296	3.606066
11	1	0	0.255651	1.094654	4.148442
12	11	0	3.232769	-0.025080	-0.251628
13	11	0	0.911799	-2.106374	2.810629
14	8	0	1.066237	-2.760070	0.548213
15	15	0	2.537984	-3.103324	0.602337
16	8	0	3.525686	-2.308509	-0.204876
17	8	0	2.966051	-3.158073	2.206410
18	8	0	2.764127	-4.710178	0.365339
19	6	0	3.074391	-4.503373	2.692836
20	6	0	2.482450	-5.392382	1.589553
21	1	0	4.139992	-4.735100	2.831027
22	6	0	2.372798	-4.629698	4.028129
23	1	0	1.394515	-5.509131	1.711271
24	1	0	2.961471	-6.376944	1.562217
25	8	0	-0.513678	-1.329191	4.436766
26	1	0	-2.231283	-0.188458	4.660359
27	1	0	-2.329984	-1.497051	3.442479
28	8	0	1.069751	-4.085147	3.942961
29	1	0	2.328881	-5.700492	4.293138
30	1	0	2.945884	-4.101586	4.807752

31	6	0	-0.786249	-2.216940	5.518736
32	6	0	-1.027193	-3.659326	5.075969
33	1	0	0.083251	-2.166468	6.191550
34	1	0	-1.660217	-1.837067	6.069973
35	6	0	0.223050	-4.513416	5.002033
36	1	0	0.773841	-4.442747	5.956588
37	1	0	-0.043261	-5.571487	4.838924
38	1	0	-1.694514	-4.140705	5.805797
39	1	0	-1.557689	-3.671398	4.109555
40	6	0	4.039886	1.680482	-3.065393
41	1	0	4.385031	2.213120	-3.957931
42	6	0	5.244825	1.154497	-2.316868
43	8	0	6.362740	1.754492	-2.690291
44	8	0	5.205996	0.305794	-1.453938
45	6	0	7.537159	1.435676	-1.936240
46	1	0	8.335298	2.067245	-2.337251
47	1	0	7.369679	1.655658	-0.873634
48	1	0	7.789524	0.375213	-2.060478
49	6	0	3.096680	0.565049	-3.448978
50	8	0	2.794868	0.560971	-4.736110
51	8	0	2.616867	-0.206503	-2.646732
52	6	0	1.820063	-0.398432	-5.161041
53	1	0	1.692066	-0.242772	-6.236125
54	1	0	2.180277	-1.415014	-4.961098
55	1	0	0.873346	-0.226325	-4.633336
56	8	0	3.821437	2.023386	0.627346
57	6	0	4.382567	3.045637	0.064766
58	6	0	4.154150	3.460344	-1.226507
59	6	0	3.245424	2.668181	-2.119231
60	1	0	4.663075	4.339712	-1.622837
61	6	0	2.289727	3.471261	-2.980842
62	1	0	2.629785	2.033791	-1.466499
63	6	0	0.946190	3.087325	-3.073217
64	6	0	0.059417	3.785151	-3.896759
65	6	0	0.507559	4.875737	-4.641105
66	6	0	1.847335	5.263023	-4.559687
67	6	0	2.731311	4.564444	-3.738382
68	1	0	0.593089	2.234130	-2.487116
69	1	0	-0.985845	3.474758	-3.952971
70	1	0	-0.183823	5.423846	-5.284128
71	1	0	2.206268	6.114913	-5.140912
72	1	0	3.779196	4.871057	-3.688106
73	6	0	5.345130	3.839562	0.916768
74	6	0	6.432204	4.539655	0.370226
75	6	0	7.305674	5.255486	1.188560
76	6	0	7.110945	5.284689	2.570835
77	6	0	6.040378	4.583424	3.127166
78	6	0	5.172684	3.860665	2.308071
79	1	0	6.609967	4.508118	-0.706895
80	1	0	8.149963	5.786594	0.743913
81	1	0	7.795035	5.844946	3.211323
82	1	0	5.880862	4.596103	4.207464
83	1	0	4.339960	3.307692	2.746264

TS-S (ω = i 446 cm[-1])

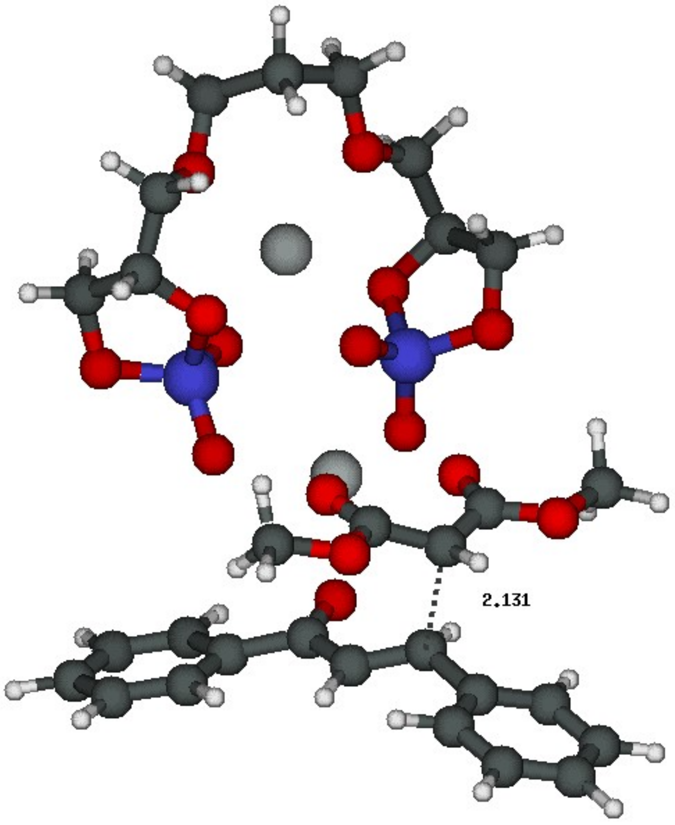

1	8	0	0.246082	0.097951	-0.039827
2	15	0	0.155968	0.063668	1.459961
3	8	0	1.421491	0.015167	2.289220
4	8	0	-0.820543	-1.168747	1.988525
5	8	0	-0.845693	1.243941	2.001850
6	6	0	-1.856602	-0.709813	2.865989
7	6	0	-1.535014	0.765793	3.159807
8	1	0	-2.816492	-0.790115	2.336578
9	6	0	-1.905986	-1.593370	4.093650
10	1	0	-2.445907	1.358705	3.294883
11	1	0	-0.889422	0.865956	4.044968
12	11	0	1.339700	-1.643426	-1.079387
13	11	0	1.051843	-2.086241	3.215515
14	8	0	1.372239	-2.845747	1.015661
15	15	0	2.888762	-2.842706	1.056501
16	8	0	3.607064	-1.942525	0.093899
17	8	0	3.320605	-2.570294	2.633784
18	8	0	3.492973	-4.362583	1.021164
19	6	0	3.801137	-3.759196	3.279747
20	6	0	3.432936	-4.914841	2.339066
21	1	0	4.893387	-3.682596	3.375459
22	6	0	3.200590	-3.874997	4.663866
23	1	0	2.416748	-5.286159	2.541043
24	1	0	4.151766	-5.738279	2.407688
25	8	0	-0.632146	-1.613545	4.709012
26	1	0	-2.660831	-1.190425	4.790944
27	1	0	-2.211113	-2.610804	3.798940
28	8	0	1.793679	-3.742469	4.595548
29	1	0	3.476410	-4.861604	5.075197
30	1	0	3.617574	-3.094899	5.321711
31	6	0	-0.594764	-2.330277	5.940428

32	6	0	-0.323724	-3.824463	5.771742
33	1	0	0.198466	-1.866977	6.546446
34	1	0	-1.551424	-2.175198	6.463118
35	6	0	1.141834	-4.211873	5.769042
36	1	0	1.641263	-3.783066	6.655963
37	1	0	1.245374	-5.309151	5.816331
38	1	0	-0.785708	-4.356012	6.616620
39	1	0	-0.818038	-4.196114	4.859320
40	6	0	0.501256	-3.947897	-3.674912
41	1	0	0.205187	-4.699653	-4.403299
42	6	0	1.655087	-4.285933	-2.868479
43	8	0	2.349198	-5.307682	-3.400403
44	8	0	2.051265	-3.717140	-1.860047
45	6	0	3.563946	-5.663166	-2.743261
46	1	0	3.999242	-6.478844	-3.329400
47	1	0	4.252467	-4.808310	-2.715814
48	1	0	3.361690	-6.002759	-1.718893
49	6	0	-0.578038	-3.166926	-3.119331
50	8	0	-1.679347	-3.229400	-3.885823
51	8	0	-0.539160	-2.436026	-2.139324
52	6	0	-2.777954	-2.409568	-3.492002
53	1	0	-3.551656	-2.551464	-4.253453
54	1	0	-3.156620	-2.721169	-2.509529
55	1	0	-2.478937	-1.353837	-3.449860
56	8	0	1.904023	-0.557601	-2.991613
57	6	0	0.979721	-0.342020	-3.821648
58	6	0	0.638507	-1.249105	-4.850934
59	6	0	1.329849	-2.472778	-4.970424
60	1	0	-0.178999	-1.019873	-5.531520
61	6	0	1.288418	-3.267966	-6.223928
62	1	0	2.295721	-2.506988	-4.458264
63	6	0	2.403111	-4.041411	-6.578832
64	6	0	2.398483	-4.803543	-7.747073
65	6	0	1.274085	-4.810580	-8.572862
66	6	0	0.156638	-4.047352	-8.225101
67	6	0	0.162448	-3.280649	-7.061849
68	1	0	3.283031	-4.038768	-5.930552
69	1	0	3.277352	-5.394429	-8.012206
70	1	0	1.267064	-5.408402	-9.486226
71	1	0	-0.727361	-4.049945	-8.865799
72	1	0	-0.721135	-2.695950	-6.797353
73	6	0	0.227144	0.960230	-3.698052
74	6	0	-0.967905	1.222170	-4.385792
75	6	0	-1.634691	2.434443	-4.213252
76	6	0	-1.117206	3.406710	-3.355725
77	6	0	0.071577	3.157460	-2.668059
78	6	0	0.734100	1.942428	-2.835157
79	1	0	-1.398846	0.475950	-5.053847
80	1	0	-2.566551	2.618818	-4.751169
81	1	0	-1.640466	4.355664	-3.222892
82	1	0	0.484013	3.911526	-1.994886
83	1	0	1.659227	1.742204	-2.293549

10a-S

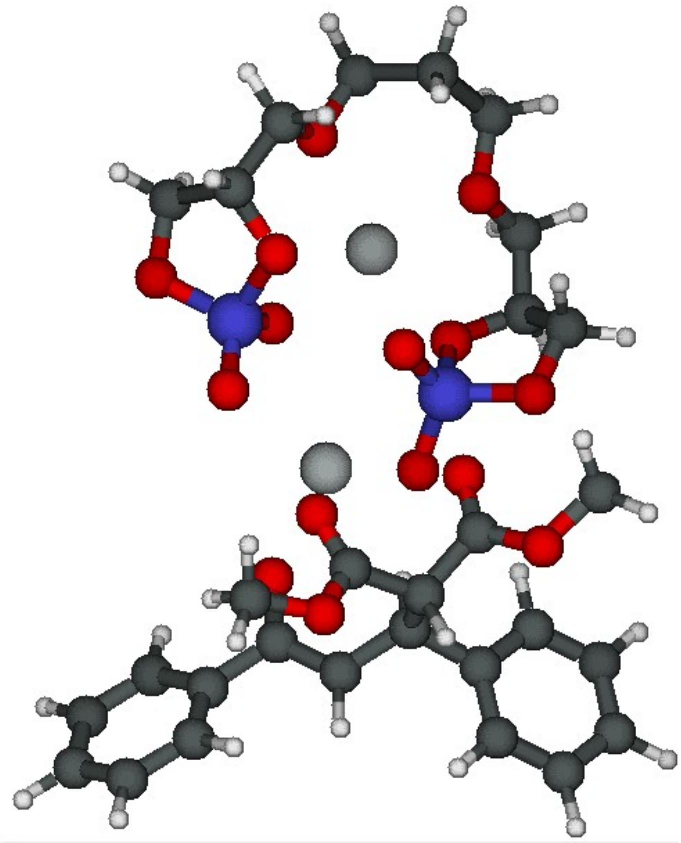

1	8	0	-0.670079	-0.202284	0.389913
2	15	0	-0.244135	-0.199641	1.831311
3	8	0	1.228666	-0.218969	2.179941
4	8	0	-0.967194	-1.420148	2.689892
5	8	0	-1.019238	0.991372	2.649740
6	6	0	-1.649952	-0.941304	3.855689
7	6	0	-1.273031	0.544931	3.983781
8	1	0	-2.731197	-1.045718	3.688141
9	6	0	-1.260269	-1.784941	5.049775
10	1	0	-2.095952	1.135699	4.400074
11	1	0	-0.370214	0.677398	4.598041
12	11	0	0.374888	-1.615786	-1.078282
13	11	0	1.226946	-2.295733	3.218512
14	8	0	0.775270	-3.074842	1.038191
15	15	0	2.215198	-3.180611	0.579122
16	8	0	2.620789	-2.371273	-0.621444
17	8	0	3.157511	-2.867978	1.906397
18	8	0	2.707585	-4.734319	0.436905
19	6	0	3.812361	-4.044803	2.402151
20	6	0	3.105623	-5.223732	1.720296
21	1	0	4.868251	-4.008636	2.098906
22	6	0	3.741559	-4.074834	3.912550
23	1	0	2.218380	-5.540920	2.289068
24	1	0	3.781190	-6.073847	1.577293
25	8	0	0.147433	-1.774374	5.190154
26	1	0	-1.737489	-1.364101	5.951750
27	1	0	-1.629497	-2.813794	4.907404
28	8	0	2.402226	-3.913048	4.339080
29	1	0	4.140452	-5.045458	4.254663
30	1	0	4.368452	-3.272548	4.335086
31	6	0	0.617212	-2.427532	6.366396

32	6	0	0.833742	-3.928646	6.187166
33	1	0	1.564184	-1.937041	6.637834
34	1	0	-0.102569	-2.242822	7.178942
35	6	0	2.210116	-4.319808	5.688281
36	1	0	2.983451	-3.848755	6.320879
37	1	0	2.339760	-5.413658	5.749069
38	1	0	0.706334	-4.414586	7.165687
39	1	0	0.056263	-4.344852	5.526087
40	6	0	0.030106	-3.621746	-3.929924
41	1	0	-0.156710	-4.127719	-4.883384
42	6	0	0.288779	-4.630876	-2.837366
43	8	0	0.411664	-5.867319	-3.294004
44	8	0	0.443148	-4.328535	-1.675393
45	6	0	0.798975	-6.862767	-2.339977
46	1	0	0.864691	-7.803382	-2.894331
47	1	0	1.774049	-6.604245	-1.906827
48	1	0	0.046130	-6.937705	-1.545470
49	6	0	-1.147822	-2.730776	-3.604587
50	8	0	-1.946296	-2.558743	-4.644223
51	8	0	-1.335957	-2.198976	-2.532502
52	6	0	-3.008148	-1.612329	-4.480646
53	1	0	-3.511611	-1.551666	-5.449885
54	1	0	-3.705987	-1.957913	-3.707764
55	1	0	-2.596395	-0.634275	-4.198907
56	8	0	0.933215	-0.166893	-2.738646
57	6	0	0.807576	-0.272403	-4.022513
58	6	0	1.019999	-1.422973	-4.745544
59	6	0	1.317968	-2.714929	-4.045073
60	1	0	0.898318	-1.422045	-5.829319
61	6	0	2.433822	-3.563255	-4.626391
62	1	0	1.608390	-2.470745	-3.013369
63	6	0	3.347459	-4.189720	-3.768577
64	6	0	4.346151	-5.023878	-4.275813
65	6	0	4.442700	-5.243456	-5.649756
66	6	0	3.534495	-4.625681	-6.512553
67	6	0	2.536686	-3.794373	-6.004232
68	1	0	3.275156	-4.014141	-2.690961
69	1	0	5.052808	-5.499537	-3.592745
70	1	0	5.223665	-5.893477	-6.049101
71	1	0	3.603348	-4.793063	-7.589391
72	1	0	1.827370	-3.322148	-6.688283
73	6	0	0.408958	0.981394	-4.766384
74	6	0	-0.317949	0.943413	-5.966905
75	6	0	-0.676431	2.120447	-6.623351
76	6	0	-0.318000	3.360560	-6.091266
77	6	0	0.392575	3.411915	-4.891413
78	6	0	0.742872	2.233693	-4.231655
79	1	0	-0.625797	-0.018035	-6.383497
80	1	0	-1.248565	2.068565	-7.552111
81	1	0	-0.599088	4.281975	-6.605200
82	1	0	0.673135	4.376480	-4.463007
83	1	0	1.291714	2.277337	-3.289232
